# Causal association of inflammatory bowel disease with sarcoidosis and the mediating role of primary biliary cholangitis

**DOI:** 10.3389/fimmu.2024.1448724

**Published:** 2024-09-03

**Authors:** Jiazhi Yi, Shuyun Wu, Hongxia He

**Affiliations:** ^1^ Department of Gastroenterology, The Affiliated Hospital of Zunyi Medical University, Zunyi, China; ^2^ Department of Gastroenterology, The Third Affiliated Hospital of Sun Yat-Sen University, Guangzhou, China; ^3^ Department of Respiratory and Critical Care Medicine, The Affiliated Hospital of Zunyi Medical University, Zunyi, China

**Keywords:** inflammatory bowel disease, sarcoidosis, mediating role, primary biliary cholangitis, Mendelian randomization

## Abstract

**Objectives:**

Previous observational epidemiological studies have identified a potential association between inflammatory bowel disease (IBD) and sarcoidosis. Nonetheless, the precise biological mechanisms underlying this association remain unclear. Therefore, we adopted a Mendelian randomization (MR) approach to investigate the causal relationship between IBD with genetic susceptibility to sarcoidosis, as well as to explore the potential mediating role.

**Methods:**

The genetic associations were obtained from publicly available genome-wide association studies (GWASs) of European ancestry. The IBD dataset has 31,665 cases and 33,977 controls, consisting of 13,768 individuals with ulcerative colitis (UC) and 17,897 individuals with Crohn’s disease (CD). The genetic associations of sarcoidosis with 4,854 cases and 446,523 controls. A bidirectional causality between IBD and sarcoidosis was implemented to be determined by a two-sample MR approach. The inverse variance weighted (IVW) method was utilized as the main statistical method, and a series of sensitivity analyses were performed to detect heterogeneity and horizontal pleiotropy. A two-step MR approach was used to investigate whether the mediating pathway from IBD to sarcoidosis was mediated by PBC.

**Results:**

The forward MR analysis indicated that genetic predisposition to IBD was significantly linked to an increased risk of sarcoidosis (OR = 1.088, 95% CI: 1.023–1.158, *p_IBD-sar_* = 7.498e-03). Similar causal associations were observed in CD (OR = 1.082, 95% CI: 1.028–1.138, *p_CD-sar_* = 2.397e-03) and UC (OR = 1.079, 95% CI: 1.006–1.158, *p_UC-sar_* = 0.034). Reverse MR analysis revealed that genetic susceptibility to sarcoidosis was correlated with an augmented risk of CD (OR = 1.306, 95% CI: 1.110–1.537, *p_sar-CD_* = 1.290e-03) but not IBD or UC. The mediation analysis via two-step MR showed that the causal influence of IBD and CD on sarcoidosis effects was partly mediated by PBC, and the mediating effect was 0.018 (95% CI: 0.005–0.031, *p* = 7.596e-03) with a mediated proportion of 21.397% in IBD, and 0.014 (95% CI: 0.004–0.024, *p* = 7.800e-03) with a mediated proportion of 17.737% in CD.

**Conclusions:**

The MR analysis provided evidence substantiating the causal effect of IBD (CD and UC) on an increased risk of sarcoidosis, with PBC playing a mediating role in IBD and CD. However, sarcoidosis only enhances the risk of developing CD, but not IBD or UC. These findings illuminate the etiology of sarcoidosis and contribute to the management of IBD patients.

## 1 Introduction

Inflammatory bowel disease (IBD), encompassing Crohn’s disease (CD) and ulcerative colitis (UC), is a chronic and nonspecific disorder of the gastrointestinal tract (1–3). Despite significant progress in understanding IBD, the comprehension of etiological factors and the complex pathogenetic process remains incomplete (4). Sarcoidosis is a multisystem granulomatous disease that can affect any body organ at any age. This disease is highly heterogeneous and has an unpredictable clinical course (5–7). The etiology of sarcoidosis remains unknown; it is plausible that the disease is an immunological disorder par excellence, caused by one or several antigen exposures, where different genes induce an altered immune response after contact with specific antigenic stimuli that initiate and possibly perpetuate the granulomatous process (6).

The relationship between IBD and sarcoidosis has recently sparked people’s curiosity. Although both have traditionally been considered separate entities, IBD and sarcoidosis share similar clinical and histopathological manifestations (8). The potential pathological changes in each disease give rise to the formation of granulomas (9, 10) and result in clinical overlap of common organs (8, 11, 12). Mitchell et al. first demonstrated nearly 50% positivity of Kveim testing in CD patients, indicating a shared mechanism between CD and sarcoidosis (13). Other observational studies have shown that an elevated ratio of CD4/CD8 lymphocytes in bronchoalveolar lavage fluid was present in both CD and sarcoidosis (14). Additionally, similar responses to mycobacterial infection were observed in CD (15) and sarcoidosis tissues (16). The coexistence of UC and sarcoidosis has also been previously reported. Moreover, several studies have suggested their immunogenicity and pathogenicity associations (17, 18). Halling et al. confirmed a significantly increased risk of sarcoidosis in patients diagnosed with UC or (and) CD (19). However, apart from these observational studies, limited knowledge exists regarding the association between IBD and sarcoidosis.

Observational studies have indicated that the incidence of autoimmune liver disease primary biliary cholangitis (PBC) is higher in IBD patients than in healthy individuals (20). IBD and PBC share certain metabolites that are causally associated with both, such as isovalerylcarnitine, and may have analogous pathogenesis (21, 22). On the other hand, clinical features of overlap between PBC and morphologically proven sarcoidosis lesions have been demonstrated (23–28). However, the cause-and-effect relationship of IBD on PBC, and the causal link between PBC and sarcoidosis, are not widely known. Moreover, whether the causal effect of IBD on sarcoidosis is mediated by the role of PBC remains unclear.

Mendelian randomization (MR) is a method frequently employed for exploring causal links between risk factors and outcomes by utilizing single-nucleotide polymorphisms (SNPs) as instrumental variables (IVs) (29–32). Here, we employed a two-sample bidirectional MR analysis to assess the potential causal relationship of IBD, including CD and UC, with the risk for sarcoidosis, and whether PBC can act as a mediator in this connection.

## 2 Methods and materials

This study was conducted following the reporting guideline of the Strengthening the Reporting of Observational Studies in Epidemiology (https://www.strobe-mr.org/).

### 2.1 Study design and data source

We used a two-sample MR design: a genetic IV analysis based on summary-level data with SNPs as instruments for the risk factor. For causal estimates from MR studies to be valid, three assumptions must be adhered to (33): (1) the genetic variants are highly associated with the exposure, (2) the genetic variants are not associated with any potential confounder of the exposure–outcome association, and (3) the variants exclusively affect the outcome through the exposure. We first performed a two-sample bidirectional MR to investigate the causal relationship of IBD, UC, and CD with sarcoidosis risk. The IVs, for IBD encompassing CD and UC subtypes, were extracted from a GWAS: the International IBD Genetics Consortium (IIBDGC) by Liu et al., including 31,665 cases and 33,977 controls of European ancestry, with 13,768 UC and 17,897 CD cases (34). The genetic association data of sarcoidosis were obtained from a GWAS that consists of 4,854 cases and 446,523 controls (https://r11.finngen.fi/pheno/D3_SARCOIDOSIS) in the European population. We obtained genetic association data of PBC from a GWAS that consists of 8,021 European cases and 16,489 European controls (35) (**Supplementary Table 1**). If there are significant causal associations between IBD (including CD and UC) and PBC and both of them have causal relations on sarcoidosis, a two-step MR analysis was performed to investigate whether the mediating pathway from IBD to sarcoidosis was mediated by PBC. All GWAS summary data are publicly available and therefore no additional ethical approval or informed consent was required. All summary statistics used were GWAS analyses and no sample overlap was observed.

### 2.2 Genetic instrumental variant selection

Genetic IVs were selected. Identified SNPs at each significance threshold (*p <* 5×10^−8^) were clumped for independence using PLINK clumping in the TwoSampleMR tool (36). Since there were too few SNPs with *p*-values less than 5×10^−8^ for sarcoidosis, we extended the threshold to 5×10^−6^ to select eligible genetic IVs, which had been applied to previous MR research (37). A strict cutoff of *R* ^2^ *<* 0.001 and a window of 10,000 kb were used for clumping with the 1000 Genomes European data as the reference panel. The proportions of trait variance explained by the identified SNPs were calculated using the following formula: *R* ^2 =^ 2 × β^2^ × MAF × (1−MAF)/(2 × MAF × (1 − MAF) × β^2 +^ 2 × MAF × (1 − MAF) × N × se(β)^2^) (38). In addition, we evaluated instrument strength using the *F* statistic, where *F* = (*R* ^2^ × (*N* − *k* − 1))/*k*(1 − *R* ^2^), to test the significant association of the genetic instruments with the exposure (39), and SNPs with *F <* 10 were excluded, as an *F ≥* 10 indicates a relatively low risk of weak instrument bias in MR analysis (40). To avoid potential confounding, we investigated each instrument SNP in the PhenoScanner GWAS database (41) to assess any previous associations (*p <* 5×10^−8^) with plausible confounders (including obesity, celiac disease, psychological stress, physical activity, trunk fat percentage, waist circumference, alcohol intake frequency, BMI, hypothyroidism, psoriasis, rheumatoid arthritis, and education), which have been reported to affect IBD (42–52), PBC (53–56), and sarcoidosis (57–59). To meet the assumption that requires instruments to be associated with the outcome only through exposure, we excluded SNPs highly linked to the outcome. The effects of SNPs on exposure and outcome were then harmonized to ensure that the β values were signed to the same alleles. After data harmonization, we removed palindromic SNPs with intermediate allele frequencies (*>*0.42) and outlier pleiotropic SNPs via the heterogeneity test (modified *Q* statistics) using RadialMR (Version 1.0) with the *p*-value threshold of 0.05 (60). The remaining SNPs were used to perform MR analysis.

### 2.3 Statistical analysis

The inverse variance weighted (IVW) method was used as the main approach in this MR analysis, which provides accurate estimates in the absence of heterogeneity and directional pleiotropy between the exposure and outcome (61–63). The heterogeneity of the IVW model was assessed by Cochran’s *Q* test. If Cochran’s *Q* test suggested significant heterogeneity (*p <* 0.05), we turned from the fixed IVW model to the random-effects model. In addition, the MR Egger method was performed to estimate the causal effect, with the capability of identifying and accounting for any directional pleiotropy (64, 65). The MR Pleiotropy RESidual Sum and Outlier (MR-PRESSO) method was used to evaluate horizontal pleiotropy (66). If horizontal pleiotropy exists, horizontal pleiotropy was corrected by removing the outlier and determining whether there are substantial variations in the causal effects before and after the outlier removal. Moreover, the MR-Egger regression intercept term was used to assess the possible presence of horizontal pleiotropy, where deviation from zero (*p <* 0.05) indicates directional pleiotropy. All statistical analyses were performed in R 4.3.1 with the package TwoSampleMR (version 0.5.7) and MRPRESSO (Version 1.0).

## 3 Results

### 3.1 The causal effect of IBD, CD, and UC on sarcoidosis

**Figure 1:**
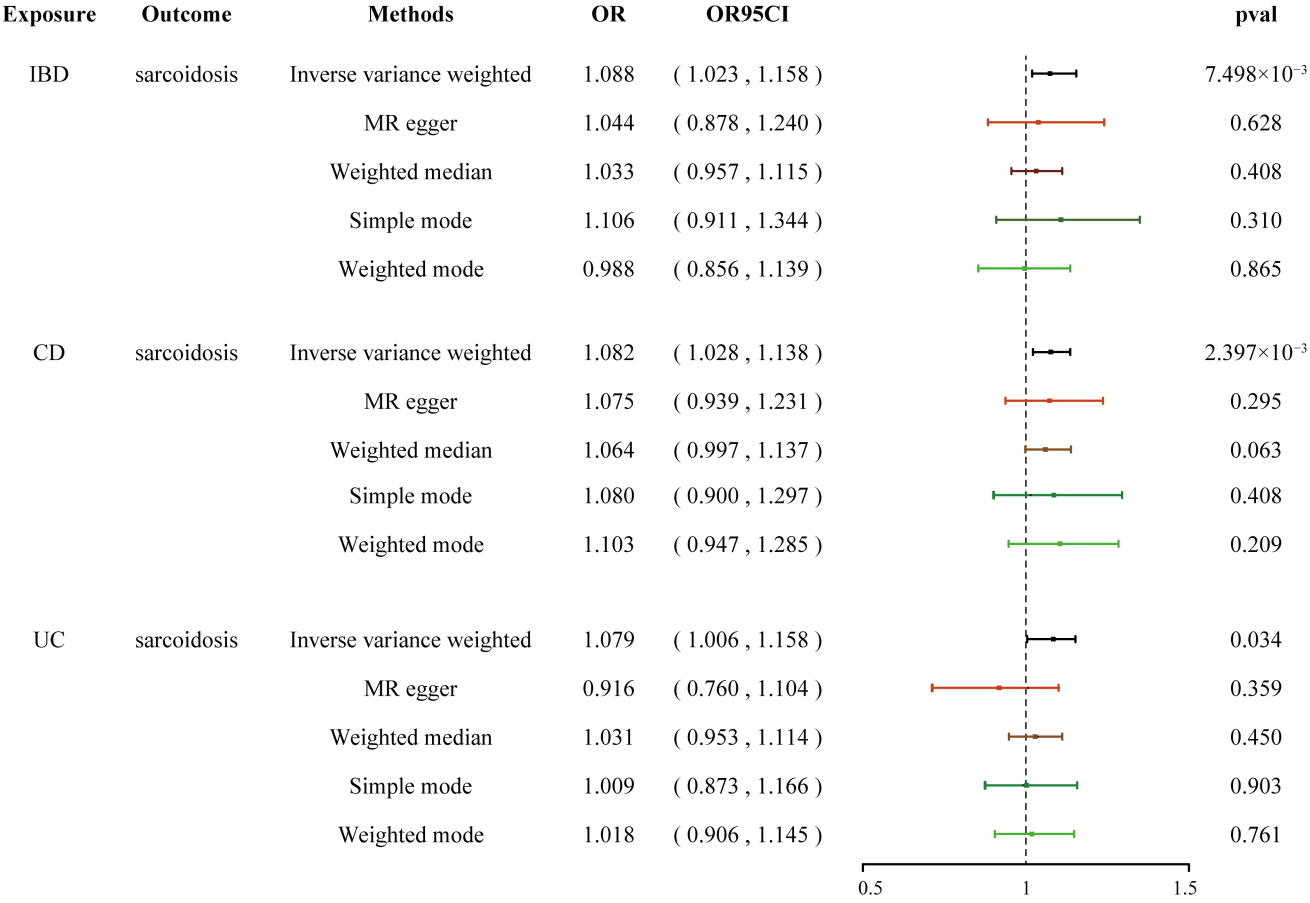
Forest plots shows the causal estimates given as OR and 95% confidence intervals for the effect of IBD, CD, and UC on sarcoidosis. IBD, inflammatory bowel disease; CD, Crohn’s disease; UC, ulcerative colitis; OR, odds ratio.

The quantity and specific characteristics of SNPs selected for IBD, CD, UC phenotype, and sarcoidosis are shown in **Supplementary Tables 2**-**6**. Based on the aforementioned selection criteria, the linkage disequilibrium test was initially conducted to select SNPs associated with IBD (encompassing CD and UC) and sarcoidosis at first. After eliminating confounders and undergoing quality control (**Supplementary Table 17**), a total of 109, 102, and 64 SNPs related to sarcoidosis in association with IBD, CD, and UC, respectively, were obtained. Cochran’s *Q* statistics test identified statistical heterogeneity using IVW and MR-Egger (IVW IBD, CD, and UC for sarcoidosis, *p* = 6.295e-10, 4.064e-09, and 2.584e-06, respectively), as listed in **Table 1**; therefore, analyses were conducted using the IVW with the multiplicative random-effects model for IBD, CD, and UC (67). We observed a significantly causal association using the IVW method between IBD (CD and UC) and sarcoidosis (OR = 1.088, 95% CI: 1.023–1.158, *p_IBD-sar_* = 7.498e-03; OR = 1.082, 95% CI: 1.028-1.138, *p_CD-sar_* = 2.397e-03; OR = 1.079, 95% CI: 1.006-1.158, *p_UC-sar_* = 0.034) (**Figure 1**). The sensitivity analysis indicated no remarkable horizontal pleiotropy (**Table 1**). Additionally, the leave-one-out analysis showed that the genetically predicted causal associations between IBD (CD and UC) and sarcoidosis were not driven by a single SNP. Funnel plot, scatter plot, and forest plot of the MR are presented (**Supplementary Figures 1**-**3**). Thus, a positive link between genetic susceptibility of IBD (CD and UC) and risk of sarcoidosis was discovered in MR. Together, IBD with its subtypes and sarcoidosis are causally associated with MR estimation results, and all sensitivity analyses as qualitative control support the idea that the causal association between IBD (UC and CD) and sarcoidosis has weak bias.

**Table 1:** Heterogeneity and pleiotropy analysis of IBD (CD and UC) and sarcoidosis using different analytical methods.

Exposure traits	Outcome traits	MR methods	Cochran’s *Q* statistic	Heterogeneity *p*-value	Pleiotropy *p*-value	MR-PRESSO global outlier test	
						RSSOBs *p*-value	*p*-value
**IBD**	**Sarcoidosis**	Inverse variance weighted	222.356	6.295e-10	0.613	327.411	<0.00025
**CD**		Inverse variance weighted	205.469	4.064e-09	0.925	240.536	<0.00025
**UC**		Inverse variance weighted	127.866	2.584e-06	0.068	155.304	<0.00025

MR, Mendelian randomization; CD, Crohn’s disease; MR-PRESSO, MR pleiotropy residual sum and outlier; IBD, inflammatory bowel disease; UC, ulcerative colitis.

### 3.2 The causal effect of sarcoidosis on IBD, CD, and UC

We extended the threshold to 5×10^−6^ to select eligible IVs (37) linked to sarcoidosis and IBD (encompassing CD and UC). The *F*-statistics for the genetic instruments were consistent with an absence of weak instrument bias (**Supplementary Table 7**). After eliminating confounders and undergoing quality control (**Supplementary Table 18**), a total of two, two, and five SNPs were obtained related to IBD, CD, and UC in association with sarcoidosis (**Supplementary Tables 8**-**10**). Cochran’s *Q* test showed no evidence for heterogeneity of IBD (*p_IBD_* = 0.051), CD (*p_CD_* = 0.683), and UC (*p_UC_* = 0.083) (**Supplementary Table 20**); therefore, analyses were conducted using the IVW method with the multiplicative fixed-effects model. The results showed that sarcoidosis had a suggestive causal effect on CD (OR = 1.306, 95% CI: 1.110–1.537, *p_sar-CD_* = 1.290e-03). However, we found no evidence of causal relationships of sarcoidosis with IBD (OR = 1.013, 95% CI: 0.796–1.289, *p_sar-IBD_* = 0.919) and UC (OR = 1.104, 95% CI: 0.970–1.257, *p_sar-CD_* = 0.132) (**Supplementary Figure 4**). The forest, funnel, and scatter plots of IBD and its sub-phenotypes are presented in **Supplementary Figures 5**-**7**. Thus, there is a positive link between genetic susceptibility of sarcoidosis on the risk of CD, but not IBD and UC.

### 3.3 Mediation analysis

**Figure 2:**
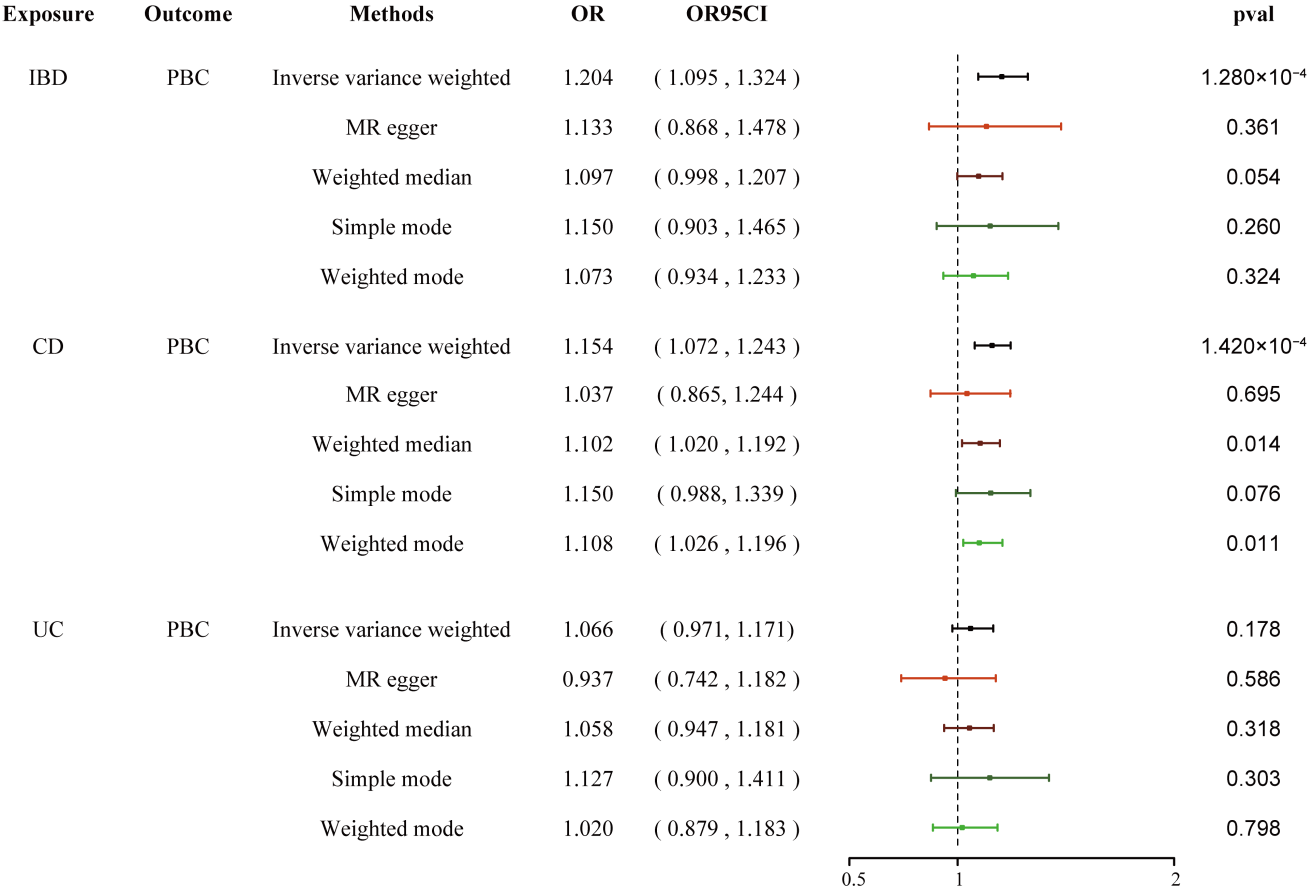
Causal estimates given as OR and 95% confidence intervals for the effect of IBD, CD, and UC on PBC. IBD, inflammatory bowel disease; CD, Crohn’s disease; UC, ulcerative colitis; PBC, primary biliary cholangitis; OR, odds ratio.

**Figure 3:**
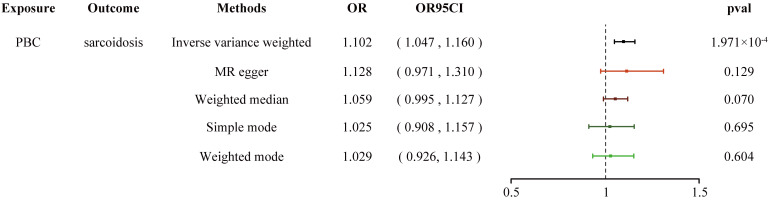
Forest plot shows the causal estimates given as OR and 95% confidence intervals for the effect of PBC on sarcoidosis. PBC, primary biliary cholangitis; OR, odds ratio.

**Figure 4:**
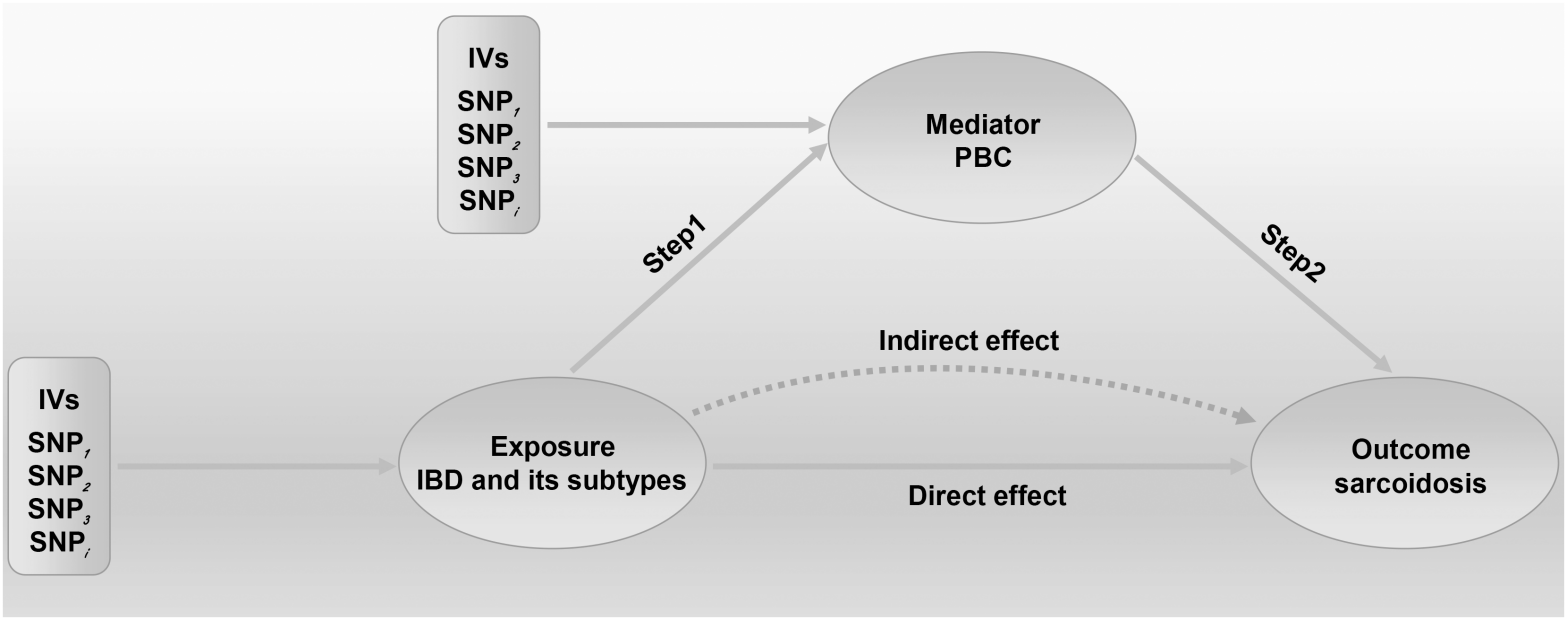
Two-step Mendelian randomization (MR) analysis framework shows the mediation analysis of the effect of IBD and CD on sarcoidosis via PBC. We extended the threshold to 5×10^−8^ or 5×10^−6^ to select eligible IVs in this MR study. Step 1 estimated the causal effect of the exposure on the potential mediator, and step 2 assessed the causal effect of the mediator on sarcoidosis risk. “Total effect” indicates the effect of IBD (CD) on sarcoidosis, “Direct effect A” indicates the effect of IBD (CD) on PBC, “Direct effect B” indicates the effect of PBC on sarcoidosis, and “mediation effect” indicates the effect of IBD (CD) on sarcoidosis through PBC. Total effect, Direct effect A, and Direct effect B were derived by IVW; IVs, instrumental variables; IVW, inverse variance weighted; IBD, inflammatory bowel disease; CD, Crohn’s disease; PBC, primary biliary cholangitis.

We conducted a two-step MR analysis to investigate the mediating pathway from IBD to sarcoidosis via PBC. In the first step, genetic instruments for IBD (encompassing CD and UC) were used to estimate the causal effect of the exposure on PBC as the potential mediator. SNPs related to potential confounders and palindromic SNPs with intermediate allele frequencies (>0.42) were removed (**Supplementary Table 19**). Then, summary information on the PBC-related phenotype for the SNPs associated with IBD (CD and UC) is listed in **Supplementary Tables 3** and **11–13**. As listed in **Table 2**, the Cochran’s *Q* statistics test identified statistical heterogeneity using IVW and MR-Egger (IVW IBD, CD, and UC for PBC, *p* = 1.195e-13, 7.371e-11, and 7.160e-05, respectively); therefore, analyses were conducted using the IVW with the multiplicative random-effects model for IBD, CD, and UC. We observed that IBD and CD were causally associated with PBC, and the IVW method showed that genetically predicted IBD (OR = 1.204, 95% CI: 1.095–1.324*, p_IBD-PBC_* = 1.280e-04) and CD (OR = 1.154, 95% CI: 1.072–1.243, *p_CD-PBC_* = 1.420e-04) were significantly associated with an increased risk of PBC. Other methods did not reach statistical significance for the causal association between IBD and PBC. Similarly, excluding MR-Egger (OR = 1.037, 95% CI: 0.865–1.244, *p* = 0.695) and the Simple model (OR = 1.150, 95% CI: 0.988–1.339, *p* = 0.076), other methods supported the nominally significant causal association between CD and PBC (weighted median OR = 1.102, 95% CI: 1.020–1.192, *p* = 0.014; weighted model OR = 1.108, 95% CI: 1.026–1.196, *p* = 0.011) (**Figure 2**). However, MR analysis as a result of the IVW model implied that genetic susceptibility to UC had no causal association with PBC (OR = 1.066, 95% CI: 0.971–1.171, *p_UC-PBC_* = 0.178), and the weighted median, the weighted mode, the Simple model, and MR-Egger yielded similar patterns of effects (**Figure 2**). The leave-one-out analysis, funnel plot, scatter plot, and forest plot of the MR of IBD, CD, and UC on PBC risk are presented in **Supplementary Figures 8**-**10**. In the second step, we assessed the causal effect of the mediator on sarcoidosis risk using genetic instruments for the PBC-related phenotype, and to avoid potential confounding, any previous associations with plausible confounders (that is celiac disease) were removed (**Supplementary Table 21**). Since the Cochran’s *Q* statistics test identified a statistical heterogeneity (IVW PBC for sarcoidosis, *p* = 0.015) (**Table 3**), the Forest plot showed causal evidence for the effect of PBC on sarcoidosis by using the IVW method with the multiplicative random-effects model (OR = 1.102, 95% CI: 1.047–1.160, *p_PBC-Sar_* = 1.971e-04). However, other methods did not reach statistical significance for the causal association (**Figure 3**, **Supplementary Figure 11**). Then, given that significant causal effects of IBD and CD on PBC and sarcoidosis were found in the abovementioned analyses, the two-step MR analysis was performed to estimate the mediation effects of PBC between IBD/CD and sarcoidosis. We estimated the indirect effect of IBD and CD on sarcoidosis via PBC and obtained the mediation effect of PBC was 0.018 (95% CI: 0.005–0.031; *p* = 7.596e-03) in IBD and 0.014 (95% CI: 0.004–0.024; *p* = 7.800e-03) in CD, with a mediated proportion of 21.397% and 17.737%, respectively (**Figure 4**, **Table 4**).

**Table 2:** Heterogeneity and pleiotropy analysis of IBD, CD, and UC with PBC using different analytical methods.

Exposure traits	Outcome traits	MR methods	Cochran’s *Q* statistic	Heterogeneity *p*-value	Pleiotropy *p*-value	MR-PRESSO global outlier test	
						RSSOBs	*p*-value
**IBD**	**PBC**	Inverse variance weighted	187.974	1.195e-13	0.632	356.081	<0.00025
**CD**		Inverse variance weighted	161.868	7.371e-11	0.211	346.558	<0.00025
**UC**		Inverse variance weighted	81.834	7.160e-05	0.242	219.944	<0.00025

MR, Mendelian randomization; CD, Crohn’s disease; MR-PRESSO, MR pleiotropy residual sum and outlier; IBD, inflammatory bowel disease; PBC, primary biliary cholangitis; UC, ulcerative colitis.

**Table 3:** Heterogeneity and pleiotropy analysis of PBC and sarcoidosis using different analytical methods.

Exposure traits	Outcome traits	MR methods	Cochran’s *Q* statistic	Heterogeneity *p*-value	Pleiotropy *p*-value	MR-PRESSO global outlier test	
						RSSOBs	*p*-value
**PBC**	**Sarcoidosis**	Inverse variance weighted	41.458	0.015	0.754	114.982	<0.00025

MR, Mendelian randomization; CD, Crohn’s disease; MR-PRESSO, MR pleiotropy residual sum and outlier; IBD, inflammatory bowel disease; PBC, primary biliary cholangitis; UC, ulcerative colitis.

**Table 4:** The mediation effect of IBD and CD on Sarcoidosis via PBC.

Mediator	Total effect	Direct effect A	Direct effect B	Mediation effect		Mediated proportion (%)
	β (95% CI)	β (95% CI)	β (95% CI)	β (95% CI)	*p*	
**PBC**	0.085 (0.023–0.146)	0.186 (0.091–0.281)	0.097 (0.046–0.149)	0.018 (0.005–0.031)	7.596e-03	21.397
**PBC**	0.100 (0.028–0.130)	0.144 (0.070–0.218)	0.097 (0.046–0.149)	0.014 (0.004–0.024)	7.800e-03	17.737

“Total effect” indicates the effect of IBD (CD) on sarcoidosis, “direct effect A” indicates the effect of IBD (CD) on PBC, “direct effect B” indicates the effect of PBC on sarcoidosis, and “mediation effect” indicates the effect of IBD (CD) on sarcoidosis through PBC. Total effect, direct effect A, and direct effect B were derived by IVW; mediation effect was derived by using the delta method. All statistical tests were two-sided. *p* < 0.05 was considered significant. CD, Crohn’s disease; IBD, inflammatory bowel disease; PBC, primary biliary cholangitis.

## 4 Discussion

In this MR analysis, we found a causal relationship between IBD and its two subtypes with sarcoidosis. Furthermore, we conducted a mediation analysis and demonstrated for the first time that the effect of IBD on sarcoidosis risk was partially mediated by PBC.

The etiology factors and the complex pathogenetic process of IBD are not yet fully understood. It has been suggested that IBD emerges in genetically susceptible individuals from a convergence of genetic risk, environmental factors, and gut microbiota (1–4). So far, several susceptible genes have been identified, many of which are clarified in other immune-mediated inflammatory diseases (IMIDs). However, apart from the extraintestinal manifestations, little is known about the association between IBD and other IMIDs. Because sarcoidosis is an IMID of unknown cause, clinicians approach it as a systemic disease with extra-pulmonary organ system manifestations including skin, lymph nodes, and the central nervous system (5–7). Evidence from observational case reports and retrospective study suggests that IBD and sarcoidosis often co-occur (8, 13, 14, 17–19), some of which indicated that IBD is linked to an increased risk of sarcoidosis (19). The GWAS has found that the HLA loci associated with IBD were also associated with sarcoidosis (68), which might affect the onset of sarcoidosis caused by IBD. However, given that there are only a few scattered reports rather than evidence from RCTs, the different study designs and the varying validities of diagnoses, population sizes, and confounders, for instance, ethnicity and economic and social status, all make the findings of these studies difficult to interpret. Moreover, it is difficult to distinguish whether it is the chronic inflammatory disease activity itself or the treatment regimen, including immunosuppressive drugs and biological agents, that increases a patient’s risk of sarcoidosis. Furthermore, it is difficult to distinguish whether it is the chronic inflammatory disease activity itself or the treatment regimen, including immunosuppressive drugs and biological agents, that increases a patient’s risk of sarcoidosis. We performed MR analysis to assess the evidence of causal association of IBD, CD, and UC with sarcoidosis. We found that the risks of sarcoidosis were elevated by 8.8%, 8.2%, and 7.9% for IBD, CD, and UC, respectively. Halling et al. (19) reported that IBD is linked to an increased risk of sarcoidosis, which is consistent with our estimate in terms of direction and magnitude. Moreover, there is a positive link between genetic susceptibility of sarcoidosis and the risk of CD; however, we found no evidence of causal relationships of sarcoidosis with IBD and UC.

Previous research indicates that the prevalence of any IMIDs is higher in IBD patients than in the general population (69). Most IMIDs are considered to be Th1 mediated, and the presence of Th17 cells in IBD and their ability to induce a Th1 response might explain this (70–73). Cumali Efe’s study has specifically analyzed the frequency of sarcoidosis in patients with PBC (74). Observational studies have shown that the frequency of PBC is higher in IBD patients (19); both share the same metabolites (21) and have an analogical pathogenesis causally associated with each other (22). The pathogenesis of PBC resembles those of CD and UC, which involves the pathogenicity and synergistic action of Th1 and Th17 cells (75–77). Moreover, some lines of evidence suggest that there is a shared genetic component between IBD and PBC (78, 79). We demonstrated that the promotion effect of IBD on sarcoidosis risk was partially mediated by PBC, which was partially in line with Zhang et al.’s recent study (80). These two simultaneous occurrences of uncommon disorders are all considered to be T cell-mediated diseases, suggesting that a common pathway might contribute to the granuloma formation in both diseases and influence each other.

Admittedly, there are limitations and restrictions in the current investigation. One of the constraints in this study is that we were compelled to rely solely on GWASs conducted in persons of European ancestry to estimate the causal effects due to the absence of extensive GWASs conducted in non-European ancestries. Thus, caution should be exercised when generalizing our findings to other ethnic groups. However, population stratification was not a potential bias in our study since European ancestry was predominant in all datasets. Then, regrettably, we must acknowledge the limitation linked to the use of the IEU GWAS database, which does not allow for subgroup analysis based on gender as conditions of IMIDs are more commonly found in female than in male patients. Therefore, we were unable to provide gender-adjusted ORs. Finally, the underlying mechanisms to determine the association of the causal pathway are needed.

In conclusion, this study demonstrated that IBD and its two subtypes CD and UC had an impact on sarcoidosis, which was partially mediated by PBC. If IBD truly increases the risk of sarcoidosis, promoting mucosal healing as a promising therapeutic strategy in IBD might help prevent PBC and sarcoidosis among individuals at elevated risk. This is of great significance in preventing their detrimental clinical process and restricting their psychological influence, and *vice versa*.

## Data Availability

The original contributions presented in the study are included in the article/**Supplementary Material**. Further inquiries can be directed to the corresponding author.
