# Supplementary material for: Causal association of inflammatory bowel disease with sarcoidosis and the mediating role of primary biliary cholangitis
Source: Front Immunol. 2024 Sep 3;15:1448724. doi: 10.3389/fimmu.2024.1448724 (PMC11406174; doi:10.3389/fimmu.2024.1448724)
Supplement: Supplementary file 1 [file DataSheet1.pdf]

Casual association of inflammatory bowel disease with sarcoidosis and the mediating role of primary biliary cholangitis

Supplementary Table 1. GWAS summary statistics: source and description

Phenotypes	Consortium	Sample size (cases)	Download
IBD	IIBDGC	65,642	https://gwas.mrcieu.ac.uk/datasets/ieu-a-294/
CD	IIBDGC	51,874	https://gwas.mrcieu.ac.uk/datasets/ieu-a-12/
UC	IIBDGC	47,745	https://gwas.mrcieu.ac.uk/datasets/ieu-a-970/
PBC	NA	24,510	https://gwas.mrcieu.ac.uk/datasets/ebi-a-GCST90061440/
Sarcoidosis	NA	451,377	https://r11.finngen.fi/pheno/D3_SARCOIDOSIS

IBD: Inflammatory bowel disease; UC: Ulcerative colitis; CD: Crohn's disease; PBC: Primary biliary cholangitis; IIBDGC: International Inflammatory Bowel Disease Genetics Consortium.

Supplementary Table 2. Summary information on IBD, CD and UC SNPs used as genetic instruments for the Mendelian randomization analyses

SNP	A1	A2	EAF	Bata	SE	N	*P*-value	R^2	F
**134 SNPs for IBD**									
rs7523442	T	C	0.5356	0.124537	0.0099006	65642	2.76E-36	0.002405	158.2194
rs34856868	A	G	0.03159	-0.195275	0.0340546	65642	9.80E-09	0.000501	32.87975
rs2488397	C	G	0.2037	0.09881	0.0121644	65642	4.55E-16	0.001004	65.97922
rs72634258	C	T	0.1757	-0.126877	0.0139965	65642	1.25E-19	0.00125	82.17007
rs2974935	T	G	0.4948	0.0687256	0.0100715	65642	8.87E-12	0.000709	46.56242
rs12411259	A	G	0.2401	0.0669054	0.0115117	65642	6.18E-09	0.000514	33.77771
rs35730213	C	G	0.2807	-0.159624	0.0113656	65642	8.33E-45	0.002996	197.2416
rs3024493	A	C	0.1572	0.196922	0.0131753	65642	1.65E-50	0.003392	223.3851
rs12103	C	T	0.8166	-0.0867354	0.0130755	65642	3.28E-11	0.00067	44.00104
rs7547569	C	T	0.06675	-0.6472	0.0232516	65642	1.65E-170	0.011665	774.7438
rs2297559	A	G	0.6822	0.0741659	0.0110454	65642	1.88E-11	0.000686	45.08504
rs6588248	G	T	0.5297	0.0819867	0.0099185	65642	1.38E-16	0.00104	68.32531
rs10800309	G	A	0.6578	-0.132111	0.010405	65642	6.15E-37	0.00245	161.2058
rs13407913	G	A	0.4306	0.0917072	0.0098818	65642	1.69E-20	0.00131	86.12329
rs78487399	C	G	0.90326	-0.132093	0.0163913	65642	7.71E-16	0.000988	64.94109
rs1517352	C	A	0.6048	0.0778816	0.0102944	65642	3.87E-14	0.000871	57.23404
rs6708373	G	A	0.5277	0.134178	0.0099341	65642	1.43E-41	0.002772	182.4284
rs7608910	G	A	0.3909	0.126444	0.0100484	65642	2.60E-36	0.002406	158.3395
rs11691685	G	A	0.08024	-0.122467	0.0187977	65642	7.27E-11	0.000646	42.44392
rs6745185	G	T	0.7389	0.0698267	0.0115253	65642	1.37E-09	0.000559	36.70501
rs780094	C	T	0.6051	-0.0783055	0.0099643	65642	3.88E-15	0.00094	61.75629
rs1420098	C	T	0.3877	-0.0952951	0.0102776	65642	1.83E-20	0.001308	85.96952
rs72924296	G	A	0.2696	-0.0638392	0.0112629	65642	1.44E-08	0.000489	32.12633
rs6740462	A	C	0.7378	0.0799597	0.0116057	65642	5.59E-12	0.000723	47.46641
rs1990760	T	C	0.6085	-0.0671151	0.0107006	65642	3.56E-10	0.000599	39.33788
rs11677953	A	G	0.3969	0.0790564	0.0100145	65642	2.92E-15	0.000948	62.31639
rs35256947	C	T	0.2594	0.0821647	0.0113176	65642	3.87E-13	0.000802	52.70461
rs9836291	A	G	0.2878	0.160867	0.0105244	65642	9.61E-53	0.003547	233.6286
rs11713774	C	T	0.1427	0.0942858	0.0142704	65642	3.92E-11	0.000665	43.65229
rs4692386	C	T	0.593	0.0579752	0.0101746	65642	1.21E-08	0.000494	32.46658
rs7657746	G	A	0.244	-0.0868542	0.0118004	65642	1.83E-13	0.000825	54.17201
rs13107612	T	C	0.2969	0.0732561	0.0108741	65642	1.62E-11	0.000691	45.38243
rs974801	G	A	0.3799	-0.0727718	0.0101382	65642	7.07E-13	0.000784	51.52183
rs3776414	G	T	0.3756	0.0773716	0.0101613	65642	2.65E-14	0.000882	57.97642
rs7711427	C	A	0.613	0.174764	0.0101797	65642	4.63E-66	0.00447	294.7276
rs4703855	T	C	0.2998	-0.0710611	0.0109034	65642	7.16E-11	0.000647	42.47435
rs36048684	A	T	0.1107	-0.0941465	0.0159649	65642	3.70E-09	0.000529	34.77465
rs4976646	C	T	0.3415	0.0730113	0.0104794	65642	3.23E-12	0.000739	48.53937
rs79980175	C	A	0.136	-0.0953162	0.0148313	65642	1.30E-10	0.000629	41.30111
rs34804116	A	C	0.3867	-0.0574631	0.0104322	65642	3.62E-08	0.000462	30.33982
rs181826	A	C	0.6266	0.0820444	0.0104471	65642	4.05E-15	0.000939	61.67273
rs1363907	A	G	0.4212	0.0815026	0.0104086	65642	4.87E-15	0.000933	61.31194
rs272882	T	G	0.6733	0.166117	0.0108875	65642	1.47E-52	0.003534	232.7869
rs71593329	G	T	0.1998	-0.0978042	0.0126731	65642	1.19E-14	0.000907	59.55744

rs56167332	A	C	0.3375	0.155855	0.0104967	65642	7.17E-50	0.003347	220.4564
rs6456426	A	C	0.4984	-0.0643405	0.0099028	65642	8.18E-11	0.000643	42.21236
rs9264942	C	T	0.3529	0.094692	0.0107773	65642	1.55E-18	0.001175	77.19575
rs9273363	A	C	0.2748	-0.193129	0.0120075	65642	3.30E-58	0.003926	258.6882
rs1847472	A	C	0.3421	-0.0672805	0.0108961	65642	6.63E-10	0.000581	38.12616
rs11758694	A	T	0.09938	0.107782	0.0160257	65642	1.75E-11	0.000689	45.23194
rs13204742	T	G	0.1267	0.0916208	0.0147604	65642	5.39E-10	0.000587	38.5282
rs1267499	C	T	0.81	0.0821443	0.0125131	65642	5.22E-11	0.000656	43.0935
rs769177	T	C	0.02548	0.260903	0.0285603	65642	6.53E-20	0.00127	83.44866
rs6933404	C	T	0.2108	0.0957518	0.0122643	65642	5.84E-15	0.000928	60.95301
rs62434177	A	G	0.03236	-0.179105	0.031375	65642	1.14E-08	0.000496	32.58628
rs9457247	T	C	0.5398	0.089151	0.0102086	65642	2.48E-18	0.00116	76.26176
rs7773324	A	G	0.6002	0.061818	0.0106192	65642	5.84E-09	0.000516	33.887
rs2328546	C	T	0.8014	0.0940162	0.0126938	65642	1.30E-13	0.000835	54.85407
rs11152949	G	A	0.3195	0.105057	0.0106717	65642	7.25E-23	0.001474	96.91021
rs3801835	T	C	0.3447	0.064107	0.0106005	65642	1.47E-09	0.000557	36.57169
rs11768997	T	G	0.1346	0.155265	0.0180166	65642	6.82E-18	0.00113	74.26569
rs6466198	T	A	0.386	0.0841312	0.0102451	65642	2.18E-16	0.001026	67.43239
rs2538470	G	A	0.6378	-0.0675599	0.0101646	65642	3.00E-11	0.000673	44.17578
rs1182188	C	T	0.2989	-0.0659028	0.0108086	65642	1.08E-09	0.000566	37.1754
rs12718244	A	G	0.4081	0.0761709	0.0100437	65642	3.35E-14	0.000875	57.51452
rs2395022	C	A	0.95885	-0.181635	0.0233963	65642	8.27E-15	0.000917	60.26865
rs7011507	A	G	0.1233	-0.0846011	0.0150825	65642	2.03E-08	0.000479	31.46242
rs7015630	C	T	0.2657	-0.0627799	0.0113167	65642	2.90E-08	0.000469	30.77432
rs10956252	G	C	0.6189	0.0834795	0.0101714	65642	2.26E-16	0.001025	67.35735
rs6651252	C	T	0.13	-0.0908484	0.014833	65642	9.08E-10	0.000571	37.51141
rs10758669	A	C	0.6504	-0.148762	0.0102138	65642	4.70E-48	0.003221	212.1271
rs7848647	C	T	0.6746	0.13239	0.0106897	65642	3.16E-35	0.002331	153.3791
rs4743820	T	C	0.7019	0.0639523	0.0108526	65642	3.80E-09	0.000529	34.72414
rs11793497	G	A	0.423	0.156206	0.0100484	65642	1.71E-54	0.003668	241.6509
rs34779708	G	T	0.3512	0.106679	0.0102408	65642	2.07E-25	0.00165	108.5118
rs11185982	C	T	0.1517	-0.077882	0.0137863	65642	1.61E-08	0.000486	31.91284
rs2497318	T	C	0.4504	-0.0635464	0.0098974	65642	1.36E-10	0.000628	41.22217
rs2050392	A	G	0.6003	0.0691178	0.0102921	65642	1.87E-11	0.000687	45.09814
rs1250566	A	G	0.3161	-0.100894	0.0110038	65642	4.77E-20	0.001279	84.06826
rs2274351	T	C	0.5373	0.0604993	0.0104442	65642	6.93E-09	0.000511	33.55344
rs12722515	A	C	0.1627	-0.0989022	0.0142955	65642	4.57E-12	0.000729	47.86301
rs2153283	A	C	0.217	-0.0859637	0.0127458	65642	1.54E-11	0.000692	45.48653
rs10761659	G	A	0.5399	0.153811	0.0100347	65642	4.97E-53	0.003566	234.9377
rs2688608	T	G	0.557	0.062403	0.0098864	65642	2.75E-10	0.000607	39.84002
rs6584281	G	A	0.5188	-0.164639	0.0099286	65642	9.36E-62	0.004171	274.9637
rs12796489	A	C	0.02286	-0.760367	0.0432241	65642	2.87E-69	0.004692	309.4435
rs11230563	T	C	0.348	-0.081194	0.0105845	65642	1.71E-14	0.000896	58.84288
rs11236797	A	C	0.4444	0.150864	0.009967	65642	9.32E-52	0.003478	229.103
rs559928	C	T	0.8128	0.094388	0.0129645	65642	3.33E-13	0.000807	53.00402
rs648541	G	A	0.3409	-0.0648616	0.0106716	65642	1.22E-09	0.000562	36.94052
rs10878302	A	T	0.92855	0.112429	0.0192561	65642	5.26E-09	0.000519	34.08841
rs1388585	A	G	0.98081	-0.30489	0.031707	65642	6.85E-22	0.001407	92.4619
rs12318183	A	C	0.3854	0.109531	0.01008	65642	1.67E-27	0.001796	118.0701

rs3184504	C	T	0.5074	-0.0600317	0.0098921	65642	1.29E-09	0.000561	36.82733
rs941823	C	T	0.7509	0.0830172	0.0115361	65642	6.19E-13	0.000788	51.78511
rs9557207	G	A	0.2231	-0.0878448	0.0120782	65642	3.52E-13	0.000805	52.89498
rs12585310	A	G	0.3136	0.0706485	0.0107636	65642	5.25E-11	0.000656	43.08018
rs6561151	A	G	0.2235	0.1	0.0118661	65642	3.53E-17	0.001081	71.01838
rs1569328	T	C	0.1702	-0.0809722	0.0136766	65642	3.21E-09	0.000534	35.05116
rs10142466	G	A	0.5065	-0.0580054	0.010146	65642	1.08E-08	0.000498	32.6839
rs55808324	A	G	0.09318	0.141213	0.016844	65642	5.13E-17	0.00107	70.28225
rs17293632	T	C	0.2364	0.107165	0.0116106	65642	2.71E-20	0.001296	85.18897
rs17651741	A	G	0.191	0.0702476	0.0126505	65642	2.81E-08	0.00047	30.83432
rs6500315	G	A	0.7753	0.0766082	0.0118778	65642	1.12E-10	0.000633	41.59731
rs62037363	C	T	0.3911	0.0987549	0.0102618	65642	6.36E-22	0.001409	92.60981
rs367569	T	C	0.2891	-0.0958166	0.011275	65642	1.93E-17	0.001099	72.21629
rs7194886	T	C	0.4357	-0.126026	0.0100135	65642	2.53E-36	0.002407	158.3927
rs2270395	T	C	0.7616	0.0778411	0.0118554	65642	5.17E-11	0.000656	43.10943
rs11641016	G	C	0.1973	-0.111288	0.0133908	65642	9.51E-17	0.001051	69.06697
rs3853824	C	T	0.6385	0.064031	0.0104099	65642	7.70E-10	0.000576	37.8333
rs4795397	G	A	0.4713	0.138343	0.0099666	65642	8.30E-44	0.002927	192.6665
rs744166	G	A	0.4204	-0.100017	0.0102076	65642	1.14E-22	0.00146	96.00351
rs9889296	A	G	0.2723	-0.104999	0.0112854	65642	1.35E-20	0.001317	86.56118
rs1292053	G	A	0.442	0.0701035	0.0098294	65642	9.89E-13	0.000774	50.86461
rs17780256	C	A	0.1927	-0.083427	0.0125693	65642	3.19E-11	0.000671	44.05324
rs7240004	G	A	0.3795	-0.0665215	0.0102898	65642	1.01E-10	0.000636	41.79237
rs67643815	T	G	0.5342	-0.0629092	0.0101798	65642	6.42E-10	0.000581	38.18885
rs2847278	T	C	0.841	-0.144528	0.0132236	65642	8.33E-28	0.001816	119.4514
rs35164067	A	G	0.2039	-0.11754	0.0127318	65642	2.66E-20	0.001297	85.22726
rs7253253	T	G	0.95432	-0.134424	0.02313	65642	6.19E-09	0.000514	33.77452
rs2024092	A	G	0.2162	0.106813	0.0121076	65642	1.12E-18	0.001184	77.82495
rs17694108	A	G	0.2797	0.0857629	0.0111165	65642	1.21E-14	0.000906	59.51817
rs516246	T	C	0.4652	0.0755599	0.0101797	65642	1.15E-13	0.000839	55.0934
rs6111031	T	C	0.1591	-0.264091	0.0147557	65642	1.23E-71	0.004856	320.3128
rs913678	C	T	0.3293	-0.0691643	0.0105418	65642	5.35E-11	0.000655	43.04485
rs6058869	T	C	0.3991	0.0556615	0.0100037	65642	2.63E-08	0.000471	30.95817
rs6074022	T	C	0.7497	-0.0742587	0.0114338	65642	8.32E-11	0.000642	42.17939
rs6062496	A	G	0.5694	0.123216	0.0102312	65642	2.11E-33	0.002205	145.0333
rs259964	G	A	0.5414	-0.0674584	0.0098349	65642	6.93E-12	0.000716	47.04569
rs2836883	A	G	0.2728	-0.168413	0.0115451	65642	3.38E-48	0.003231	212.7858
rs1297258	T	C	0.4249	-0.114524	0.0100654	65642	5.38E-30	0.001968	129.4547
rs8127691	C	T	0.6132	-0.114259	0.0100818	65642	8.98E-30	0.001953	128.4374
rs140143	T	G	0.3897	-0.133073	0.0114182	65642	2.18E-31	0.002065	135.8224
rs2143178	C	T	0.1658	-0.176684	0.0137017	65642	4.80E-38	0.002527	166.277
**122 SNPs for CD**									
rs17129991	T	C	0.02246	-0.284461	0.0450901	51874	2.81E-10	0.000767	39.79847
rs17391694	T	C	0.1226	-0.119051	0.0199954	51874	2.62E-09	0.000683	35.44779
rs2974935	T	G	0.4948	0.0755507	0.0121937	51874	5.80E-10	0.000739	38.38745
rs10800309	G	A	0.6578	-0.0904086	0.0126389	51874	8.48E-13	0.000985	51.16632
rs10798069	T	G	0.4925	-0.070387	0.0119822	51874	4.25E-09	0.000665	34.50603
rs6679677	A	C	0.09755	-0.185152	0.0220556	51874	4.67E-17	0.001357	70.46968
rs2641348	G	A	0.1077	-0.121336	0.0198422	51874	9.65E-10	0.00072	37.39237

rs12411259	A	G	0.2401	0.134364	0.0137443	51874	1.43E-22	0.001839	95.5661
rs3024505	A	G	0.1573	0.165311	0.0159634	51874	3.95E-25	0.002063	107.2349
rs6702421	T	C	0.2255	0.110194	0.0141395	51874	6.53E-15	0.001169	60.73388
rs7517847	G	T	0.4352	-0.335814	0.0124758	51874	1.38E-159	0.013775	724.5094
rs35730213	C	G	0.2807	-0.151105	0.0138184	51874	7.84E-28	0.0023	119.5709
rs36016881	G	A	0.1754	-0.108961	0.0170379	51874	1.60E-10	0.000788	40.89717
rs77981966	T	C	0.07271	0.182656	0.0222451	51874	2.19E-16	0.001298	67.41902
rs13001325	T	C	0.3757	-0.122687	0.0125708	51874	1.68E-22	0.001833	95.2477
rs11691685	G	A	0.08024	-0.157687	0.0233162	51874	1.35E-11	0.000881	45.73614
rs1517352	C	A	0.6048	0.0800097	0.0124517	51874	1.31E-10	0.000795	41.2868
rs6738394	A	G	0.4519	0.0777406	0.011991	51874	8.98E-11	0.00081	42.03086
rs12694846	G	A	0.2593	0.115366	0.0136242	51874	2.50E-17	0.00138	71.69963
rs7608910	G	A	0.3909	0.120609	0.0121405	51874	2.95E-23	0.001899	98.68918
rs6738490	C	T	0.5274	0.226164	0.0120892	51874	4.26E-78	0.006702	349.9734
rs13407913	G	A	0.4306	0.114508	0.0119519	51874	9.64E-22	0.001766	91.78697
rs780094	C	T	0.6051	-0.116483	0.0120611	51874	4.56E-22	0.001795	93.2684
rs35320439	C	T	0.3104	0.0840555	0.0137546	51874	9.89E-10	0.000719	37.34389
rs6740462	A	C	0.7378	0.099737	0.0141387	51874	1.74E-12	0.000958	49.7596
rs11713774	C	T	0.1427	0.132662	0.0171654	51874	1.09E-14	0.00115	59.72669
rs3197999	A	G	0.2812	0.155114	0.0128775	51874	2.05E-33	0.002789	145.0849
rs7438704	G	A	0.6435	0.0839019	0.0126601	51874	3.42E-11	0.000846	43.91901
rs34592089	A	G	0.05297	0.199406	0.0249741	51874	1.41E-15	0.001227	63.74997
rs6827756	C	T	0.6251	-0.0789645	0.0125632	51874	3.27E-10	0.000761	39.50449
rs1363907	A	G	0.4212	0.102575	0.0125984	51874	3.89E-16	0.001276	66.28809
rs3776414	G	T	0.3756	0.0888353	0.012297	51874	5.04E-13	0.001005	52.18625
rs7711427	C	A	0.613	0.24803	0.0124713	51874	5.17E-88	0.007567	395.5198
rs79980175	C	A	0.136	-0.134258	0.0182147	51874	1.70E-13	0.001046	54.32748
rs71624119	A	G	0.2422	-0.0923221	0.0149485	51874	6.57E-10	0.000735	38.14164
rs11167518	A	C	0.08143	0.279727	0.0198222	51874	3.21E-45	0.003824	199.1353
rs4703855	T	C	0.2998	-0.073261	0.0132248	51874	3.03E-08	0.000591	30.68674
rs17622378	G	A	0.4186	0.190197	0.0120781	51874	7.17E-56	0.004758	247.9667
rs34804116	A	C	0.3867	-0.0939086	0.0126747	51874	1.27E-13	0.001057	54.89321
rs181826	A	C	0.6266	0.0995948	0.0127047	51874	4.53E-15	0.001183	61.45083
rs17388425	G	C	0.1598	-0.157253	0.0172142	51874	6.54E-20	0.001606	83.44637
rs56163845	G	A	0.3096	-0.091908	0.0134853	51874	9.40E-12	0.000895	46.44818
rs9264942	C	T	0.3529	0.150794	0.0128298	51874	6.78E-32	0.002656	138.1375
rs9494844	A	C	0.2519	-0.088944	0.0142376	51874	4.18E-10	0.000752	39.02501
rs7773324	A	G	0.6002	0.078744	0.012909	51874	1.06E-09	0.000717	37.20771
rs1847472	A	C	0.3421	-0.0853854	0.0132299	51874	1.09E-10	0.000802	41.65214
rs9457247	T	C	0.5398	0.123663	0.0124045	51874	2.08E-23	0.001912	99.38121
rs6908425	C	T	0.7843	0.103988	0.0150464	51874	4.81E-12	0.00092	47.76222
rs6456426	A	C	0.4984	-0.0991795	0.0119962	51874	1.37E-16	0.001316	68.35018
rs11152949	G	A	0.3195	0.133849	0.0128549	51874	2.18E-25	0.002086	108.4117
rs438475	A	G	0.1322	0.159076	0.0172814	51874	3.42E-20	0.001631	84.72952
rs1267501	C	T	0.8106	0.0871787	0.0151984	51874	9.69E-09	0.000634	32.90094
rs3129871	C	A	0.6601	-0.087922	0.0130803	51874	1.80E-11	0.00087	45.17966
rs9491892	G	T	0.1496	0.137869	0.0163762	51874	3.80E-17	0.001364	70.87454
rs212388	T	C	0.6043	-0.102377	0.0124319	51874	1.80E-16	0.001306	67.81301
rs3801810	A	G	0.2337	0.105079	0.01402	51874	6.63E-14	0.001082	56.1719

rs1456896	T	C	0.6894	0.0977081	0.0131392	51874	1.03E-13	0.001065	55.29763
rs11768997	T	G	0.1346	0.226326	0.0208449	51874	1.83E-27	0.002267	117.8834
rs2395022	C	A	0.95885	-0.177152	0.0281553	51874	3.13E-10	0.000763	39.58722
rs2538470	G	A	0.6378	-0.0749643	0.0122847	51874	1.05E-09	0.000717	37.23601
rs7786444	T	C	0.1163	0.112085	0.0183382	51874	9.83E-10	0.00072	37.35638
rs7015630	C	T	0.2657	-0.0842498	0.0137523	51874	9.00E-10	0.000723	37.52929
rs6651252	C	T	0.13	-0.149029	0.0183017	51874	3.86E-16	0.001277	66.30434
rs10956252	G	C	0.6189	0.118641	0.0123641	51874	8.34E-22	0.001772	92.07205
rs7848647	C	T	0.6746	0.141308	0.0129961	51874	1.55E-27	0.002274	118.2199
rs10758669	A	C	0.6504	-0.14975	0.012299	51874	4.19E-34	0.00285	148.2441
rs11793497	G	A	0.423	0.168922	0.0121801	51874	9.80E-44	0.003694	192.3331
rs34779708	G	T	0.3512	0.134457	0.0123872	51874	1.90E-27	0.002266	117.8159
rs1250573	A	G	0.3155	-0.141693	0.0134477	51874	5.86E-26	0.002136	111.0156
rs7085798	A	C	0.5176	-0.174348	0.0120372	51874	1.53E-47	0.004028	209.781
rs61839660	T	C	0.08983	0.148159	0.0203346	51874	3.19E-13	0.001022	53.08454
rs303429	T	C	0.5997	0.0762756	0.0124275	51874	8.38E-10	0.000726	37.66925
rs11185982	C	T	0.1517	-0.109139	0.0168972	51874	1.05E-10	0.000804	41.71706
rs2153283	A	C	0.217	-0.108804	0.0155227	51874	2.39E-12	0.000946	49.12906
rs10995271	C	G	0.6098	-0.187653	0.012242	51874	4.92E-53	0.004509	234.9576
rs2227551	T	G	0.7288	0.0994456	0.0137489	51874	4.72E-13	0.001008	52.31407
rs12796489	A	C	0.02286	-0.79177	0.0526931	51874	4.96E-51	0.004334	225.774
rs559928	C	T	0.8128	0.099124	0.0158237	51874	3.75E-10	0.000756	39.23962
rs11236797	A	C	0.4444	0.180737	0.0120572	51874	8.54E-51	0.004313	224.6904
rs34787213	T	C	0.1401	-0.14973	0.0183059	51874	2.85E-16	0.001288	66.89886
rs3184504	C	T	0.5074	-0.067651	0.0119969	51874	1.71E-08	0.000613	31.79755
rs7969592	G	A	0.4746	-0.0731898	0.011991	51874	1.04E-09	0.000718	37.25406
rs10878302	A	T	0.92855	0.15677	0.023765	51874	4.20E-11	0.000838	43.51445
rs28999107	T	G	0.4387	0.0856594	0.0126539	51874	1.29E-11	0.000883	45.82311
rs76906269	G	A	0.01883	0.394341	0.0370279	51874	1.75E-26	0.002182	113.4146
rs9554587	G	A	0.2242	-0.0951561	0.0146501	51874	8.29E-11	0.000813	42.18668
rs9594766	A	G	0.5294	-0.0735547	0.0121434	51874	1.39E-09	0.000707	36.68796
rs1927681	A	T	0.443	0.0887872	0.0121244	51874	2.42E-13	0.001033	53.62453
rs915286	A	G	0.5488	0.066645	0.0119709	51874	2.59E-08	0.000597	30.99308
rs6561151	A	G	0.2235	0.147148	0.0142318	51874	4.68E-25	0.002057	106.8987
rs11159833	T	C	0.08681	0.155227	0.0207601	51874	7.59E-14	0.001077	55.90606
rs1569328	T	C	0.1702	-0.109215	0.0167191	51874	6.47E-11	0.000822	42.66994
rs72727394	T	C	0.2007	0.103233	0.0149661	51874	5.28E-12	0.000916	47.57766
rs17293632	T	C	0.2364	0.128384	0.0139602	51874	3.70E-20	0.001628	84.57106
rs6500315	G	A	0.7753	0.145539	0.0146056	51874	2.18E-23	0.00191	99.28948
rs1646019	T	C	0.303	-0.111379	0.013383	51874	8.62E-17	0.001333	69.26007
rs2270395	T	C	0.7616	0.124328	0.0144786	51874	8.93E-18	0.001419	73.73404
rs11117431	G	A	0.1978	-0.14927	0.0164404	51874	1.09E-19	0.001587	82.43347
rs7194886	T	C	0.4357	-0.226986	0.0121749	51874	1.42E-77	0.006656	347.5767
rs26528	C	T	0.4576	0.119845	0.0122466	51874	1.29E-22	0.001843	95.76174
rs9889296	A	G	0.2723	-0.143097	0.0137815	51874	2.96E-25	0.002074	107.8081
rs4795397	G	A	0.4713	0.132338	0.0120311	51874	3.84E-28	0.002327	120.9878
rs12949918	C	T	0.4185	-0.104177	0.0123587	51874	3.47E-17	0.001368	71.05283
rs1292053	G	A	0.442	0.0911854	0.0118917	51874	1.75E-14	0.001132	58.79576
rs3853824	C	T	0.6385	0.0813612	0.0126283	51874	1.17E-10	0.0008	41.50765

rs2847293	T	A	0.8404	-0.166911	0.0158476	51874	6.14E-26	0.002134	110.9242
rs7236492	T	C	0.1537	-0.0996611	0.017342	51874	9.09E-09	0.000636	33.0245
rs35164067	A	G	0.2039	-0.143373	0.015563	51874	3.19E-20	0.001633	84.86554
rs17694108	A	G	0.2797	0.0795895	0.0134523	51874	3.29E-09	0.000674	35.00271
rs640466	C	T	0.3739	-0.0758092	0.0124963	51874	1.31E-09	0.000709	36.80139
rs2024092	A	G	0.2162	0.147664	0.0143378	51874	7.13E-25	0.002041	106.0639
rs516246	T	C	0.4652	0.114576	0.0123121	51874	1.33E-20	0.001667	86.59769
rs6111031	T	C	0.1591	-0.282366	0.018121	51874	9.61E-55	0.004659	242.7972
rs6074022	T	C	0.7497	-0.0963211	0.0137745	51874	2.70E-12	0.000942	48.89612
rs259964	G	A	0.5414	-0.0713342	0.0119064	51874	2.08E-09	0.000691	35.89367
rs6062496	A	G	0.5694	0.120078	0.0124099	51874	3.82E-22	0.001802	93.62107
rs1297258	T	C	0.4249	-0.127246	0.0122169	51874	2.11E-25	0.002087	108.48
rs2284553	G	A	0.5904	0.103192	0.0123247	51874	5.63E-17	0.00135	70.10074
rs8127691	C	T	0.6132	-0.123357	0.0121887	51874	4.48E-24	0.001971	102.4227
rs2413583	T	C	0.1654	-0.210407	0.0168361	51874	7.72E-36	0.003002	156.1783
rs140143	T	G	0.3897	-0.126945	0.013562	51874	7.95E-21	0.001686	87.61268
rs727563	T	C	0.7973	-0.0921252	0.014461	51874	1.88E-10	0.000782	40.58294
**88 SNPs for UC**									
rs12132349	A	T	0.2809	-0.166942	0.0143783	47745	3.64E-31	0.002816	134.8025
rs3024493	A	C	0.1572	0.226278	0.0163471	47745	1.42E-43	0.003997	191.5954
rs111830527	A	G	0.05254	-0.192324	0.0292804	47745	5.09E-11	0.000903	43.14145
rs7547569	C	T	0.06675	-0.495701	0.0291648	47745	8.71E-65	0.006014	288.8706
rs12103	C	T	0.8166	-0.0995588	0.0162944	47745	9.96E-10	0.000781	37.33055
rs10910092	G	A	0.4676	-0.0863506	0.0127821	47745	1.42E-11	0.000955	45.63606
rs35223180	T	G	0.1791	-0.141003	0.0175766	47745	1.04E-15	0.001346	64.353
rs6426833	A	G	0.536	0.232387	0.0125836	47745	3.77E-76	0.007092	341.0324
rs4656958	G	A	0.6821	0.0824158	0.0138702	47745	2.82E-09	0.000739	35.30509
rs1801274	G	A	0.4958	-0.170896	0.012653	47745	1.43E-41	0.003806	182.4142
rs16841904	T	C	0.2028	0.0860225	0.015305	47745	1.90E-08	0.000661	31.58925
rs7608910	G	A	0.3909	0.127099	0.0126851	47745	1.25E-23	0.002098	100.3872
rs1990760	T	C	0.6085	-0.0855732	0.0134145	47745	1.78E-10	0.000852	40.69194
rs13430791	A	G	0.1202	0.105886	0.0186614	47745	1.39E-08	0.000674	32.19367
rs9941524	G	A	0.4559	0.0977156	0.0127881	47745	2.15E-14	0.001221	58.3845
rs10460566	A	G	0.7611	-0.0817673	0.0144699	47745	1.60E-08	0.000668	31.93082
rs4973341	T	C	0.6627	0.073473	0.0131405	47745	2.25E-08	0.000654	31.26177
rs10185424	G	T	0.5396	-0.0965995	0.0125621	47745	1.47E-14	0.001237	59.12989
rs1517352	C	A	0.6048	0.0777839	0.0129859	47745	2.10E-09	0.000751	35.87708
rs4676410	A	G	0.2038	0.142022	0.0157426	47745	1.85E-19	0.001702	81.38421
rs11676348	T	C	0.4763	0.0744362	0.0124232	47745	2.08E-09	0.000751	35.89907
rs9836291	A	G	0.2878	0.170257	0.0132458	47745	8.20E-38	0.003448	165.2096
rs13136827	C	T	0.1622	-0.111814	0.0176452	47745	2.35E-10	0.00084	40.15328
rs3774937	C	T	0.3257	0.099276	0.0131621	47745	4.61E-14	0.00119	56.88791
rs56167332	A	C	0.3375	0.141368	0.0131737	47745	7.27E-27	0.002406	115.1512
rs272882	T	G	0.6733	0.145856	0.0138588	47745	6.67E-26	0.002315	110.759
rs36070529	A	G	0.1999	-0.091704	0.0160211	47745	1.04E-08	0.000686	32.76225
rs4976646	C	T	0.3415	0.0787748	0.0132165	47745	2.52E-09	0.000744	35.52413
rs3776414	G	T	0.3756	0.0704798	0.0128464	47745	4.10E-08	0.00063	30.09874
rs7711427	C	A	0.613	0.088938	0.0127983	47745	3.67E-12	0.00101	48.28942
rs7738430	C	T	0.02624	0.367964	0.0340763	47745	3.51E-27	0.002436	116.5971

rs4947328	G	A	0.02366	0.239455	0.038129	47745	3.38E-10	0.000825	39.43836
rs9271858	G	A	0.511	0.122709	0.0129368	47745	2.42E-21	0.001881	89.96652
rs4712520	C	T	0.8182	0.0929892	0.0166206	47745	2.21E-08	0.000655	31.30066
rs2516440	A	G	0.3222	-0.0997745	0.0137762	47745	4.40E-13	0.001097	52.452
rs9271255	T	C	0.732	-0.284959	0.013809	47745	1.31E-94	0.00884	425.8161
rs34659678	T	C	0.05734	0.209947	0.0250948	47745	5.95E-17	0.001464	69.98963
rs6920220	A	G	0.2086	0.146936	0.015222	47745	4.78E-22	0.001948	93.17401
rs1077773	A	G	0.5238	0.0721282	0.012398	47745	5.96E-09	0.000708	33.84455
rs12718244	A	G	0.4081	0.0717762	0.0126545	47745	1.41E-08	0.000673	32.17013
rs1182188	C	T	0.2989	-0.107615	0.0137507	47745	5.03E-15	0.001281	61.24601
rs2395022	C	A	0.95885	-0.183896	0.029166	47745	2.88E-10	0.000832	39.75325
rs4728142	A	G	0.439	0.0969671	0.0126655	47745	1.92E-14	0.001226	58.61192
rs76546301	A	G	0.01822	0.26496	0.0410174	47745	1.05E-10	0.000873	41.72595
rs6466198	T	A	0.386	0.133923	0.0128457	47745	1.90E-25	0.002271	108.6867
rs13255292	T	C	0.3284	-0.075463	0.0137225	47745	3.82E-08	0.000633	30.24012
rs4366152	C	T	0.68	0.120371	0.0135814	47745	7.79E-19	0.001643	78.54824
rs10870077	G	C	0.5722	-0.135741	0.0126242	47745	5.77E-27	0.002416	115.6102
rs10758669	A	C	0.6504	-0.143218	0.0128828	47745	1.04E-28	0.002582	123.5824
rs4743820	T	C	0.7019	0.0809228	0.0137572	47745	4.05E-09	0.000724	34.599
rs10748783	A	C	0.5237	-0.164756	0.0126394	47745	7.73E-39	0.003546	169.907
rs2274351	T	C	0.5373	0.0710979	0.0130334	47745	4.90E-08	0.000623	29.75637
rs2497318	T	C	0.4504	-0.0714375	0.0125164	47745	1.15E-08	0.000682	32.57433
rs4747886	T	C	0.4081	0.0738479	0.0128701	47745	9.58E-09	0.000689	32.92261
rs59418206	A	G	0.3508	0.0735513	0.012978	47745	1.45E-08	0.000672	32.11789
rs10761659	G	A	0.5399	0.117315	0.0126238	47745	1.50E-20	0.001806	86.35922
rs12796489	A	C	0.02286	-0.67649	0.0559623	47745	1.22E-33	0.003051	146.1213
rs61893460	A	G	0.4447	0.121119	0.0125424	47745	4.60E-22	0.001949	93.24919
rs11229555	T	G	0.2515	-0.0823351	0.0144507	47745	1.21E-08	0.000679	32.46191
rs11230563	T	C	0.348	-0.0750666	0.0133556	47745	1.90E-08	0.000661	31.58992
rs661054	G	A	0.3408	-0.124858	0.0135529	47745	3.18E-20	0.001774	84.8692
rs483905	A	G	0.289	0.0849927	0.0135107	47745	3.16E-10	0.000828	39.57212
rs12318183	A	C	0.3854	0.162178	0.0126604	47745	1.44E-37	0.003425	164.0857
rs76904798	T	C	0.1368	0.104624	0.0176013	47745	2.78E-09	0.000739	35.33092
rs941823	C	T	0.7509	0.108669	0.0146906	47745	1.39E-13	0.001145	54.71597
rs1927681	A	T	0.443	-0.47866	0.0126594	47745	1.00E-200	0.029073	1429.585
rs55808324	A	G	0.09318	0.127209	0.021035	47745	1.47E-09	0.000765	36.57063
rs7404095	C	T	0.5796	0.0717686	0.0126826	47745	1.52E-08	0.00067	32.02095
rs79045992	A	G	0.1033	0.118093	0.02083	47745	1.43E-08	0.000673	32.14042
rs11150589	C	T	0.5268	-0.0798521	0.0127057	47745	3.28E-10	0.000827	39.49638
rs11641184	A	C	0.4762	0.0780232	0.0124939	47745	4.24E-10	0.000816	38.99719
rs4795397	G	A	0.4713	0.139853	0.012577	47745	1.01E-28	0.002583	123.6435
rs9891119	C	A	0.3536	-0.0895467	0.0133101	47745	1.72E-11	0.000947	45.2604
rs17780256	C	A	0.1927	-0.115389	0.0160316	47745	6.13E-13	0.001084	51.80324
rs7240004	G	A	0.3795	-0.0823655	0.0130179	47745	2.50E-10	0.000838	40.03046
rs8096327	G	A	0.3839	0.093806	0.0127914	47745	2.24E-13	0.001125	53.77828
rs17694108	A	G	0.2797	0.0958444	0.0139396	47745	6.17E-12	0.000989	47.27316
rs12720356	C	A	0.08572	0.153279	0.0227683	47745	1.67E-11	0.000948	45.31958
rs11083840	G	T	0.4025	0.0691316	0.0125262	47745	3.41E-08	0.000638	30.45765
rs6111031	T	C	0.1591	-0.260863	0.0190685	47745	1.33E-42	0.003904	187.1431

rs6062496	A	G	0.5694	0.113898	0.0128769	47745	9.14E-19	0.001636	78.23325
rs913678	C	T	0.3293	-0.0757579	0.013301	47745	1.23E-08	0.000679	32.43912
rs4812833	A	G	0.5188	0.103346	0.0125571	47745	1.87E-16	0.001417	67.73146
rs1297256	T	C	0.4249	-0.10106	0.0127351	47745	2.10E-15	0.001317	62.97029
rs4456788	A	G	0.6106	-0.102758	0.0127344	47745	7.07E-16	0.001362	65.11126
rs2836883	A	G	0.2728	-0.227134	0.0147424	47745	1.47E-53	0.004947	237.3612
rs9611131	C	T	0.1477	-0.142696	0.0181548	47745	3.84E-15	0.001292	61.77638
rs140143	T	G	0.3897	-0.129716	0.0143011	47745	1.19E-19	0.00172	82.26783

SNP: single nucleotide polymorphism; A1: effect allele; A2: baseline allele; EAF: effect allele frequency; SE: standard error. N refers to the sample size of the initial GWAS from which the genetic variants were selected. All statistical tests were two-sided. A *P*-value$<5\times10^{-8}$ was considered genome-wide significant.

Supplementary Table 3. Potential confounders of IBD, CD and UC SNPs under the condition of $P<5\times10^{-8}$ in the PhenoScanner database

Exposure	Excluded SNP	Trait
IBD	rs13407913[ab]	Body mass index, Body fat percentage, Trunk fat percentage, Whole body fat mass, Trunk fat mass, Waist circumference, Body mass index adjusted for physical activity, Overweight, Body mass index in physically active individuals, Obesity class 1, Obesity class 2, Waist circumference adjusted for smoking, Waist circumference in non-smokers
	rs9836291[ab]	Years of educational attainment in males, Age completed full time education, Job involves heavy manual or physical work, Job involves mainly walking or standing, Years of educational attainment in females, Qualifications: A levels or as levels or equivalent, Years of educational attainment, Qualifications: college or university degree, Body mass index, Whole body fat mass, Body fat percentage, Trunk fat mass, Trunk fat percentage
	rs9273363[ab]	Self-reported malabsorption or coeliac disease, Body mass index, Body mass index males
	rs1182188[a]	Trunk fat mass, Whole body fat mass, Trunk fat percentage, Waist circumference, Body fat percentage
	rs3184504[ab]	Celiac disease, Body mass index
	rs62037363[ab]	Years of educational attainment, Qualifications: college or university degree, Trunk fat mass, Trunk fat percentage, Whole body fat mass, Body fat percentage, Waist circumference, Body mass index, Alcohol intake frequency, Types of physical activity in last 4 weeks: light diy, Body mass index females
	rs1267499[a]	Qualifications: college or university degree, Years of educational attainment
	rs2488397[a]	Worrier or anxious feelings
	rs7657746[a]	Self-reported malabsorption or coeliac disease
	rs9264942[a]	Self-reported malabsorption or coeliac disease
	rs6933404[ab]	Celiac disease, Self-reported malabsorption or coeliac disease, Rheumatoid arthritis and celiac disease
	rs780094[ab]	Alcohol intake frequency, Fasting glucose related traits interaction with BMI, log Fasting insulin adjusted for BMI, Fasting blood glucose in high BMI subjects, Alcohol intake versus 10 years previously, Fasting insulin related traits interaction with BMI, Fasting insulin in high BMI subjects,
	rs516246[b]	Alcohol intake frequency
CD	rs17391694[ab]	Body mass index, Trunk fat mass, Trunk fat percentage, Whole body fat mass, Waist circumference, Body fat percentage
	rs13407913[ab]	Body fat percentage, Body mass index, Trunk fat mass, Overweight, Obesity with early age of onset age 2, Obesity class 1, Trunk fat percentage, Waist circumference, Body mass index adjusted for smoking
	rs3197999[ab]	Years of educational attainment in males, Age completed full time education, Fed-up feelings, Job involves heavy manual or physical work, Job involves mainly walking or standing, Years of educational attainment in females, Years of educational attainment, Qualifications: A levels or as levels or equivalent, Qualifications: college or university degree, Body fat percentage, Body mass index, Trunk fat mass, Trunk fat percentage, Whole body fat mass
	rs3184504[ab]	Celiac disease, Body mass index

	rs26528[ab]	Qualifications: college or university degree, Alcohol intake frequency, Types of physical activity in last 4 weeks: light diy, Trunk fat mass, Whole body fat mass, Body fat percentage, Waist circumference, Body mass index
	rs727563[a]	Trunk fat percentage
	rs1267501[a]	Years of educational attainment, Qualifications: college or university degree
	rs780094[ab]	Alcohol intake frequency, Alcohol intake versus 10 years previously, Fasting insulin in high BMI subjects
	rs6702421[a]	Worrier or anxious feelings
	rs9264942[a]	Self-reported malabsorption or celiac disease
	rs3129871[a]	Self-reported malabsorption or celiac disease
	rs212388[ab]	Celiac disease and Crohn's disease, Rheumatoid arthritis and celiac disease
	rs516246[b]	Alcohol intake frequency
**UC**	rs9836291[ab]	Years of educational attainment in males, Age completed full time education, Job involves heavy manual or physical work, Job involves mainly walking or standing, Years of educational attainment in females, Qualifications: A levels or as levels or equivalent, Years of educational attainment, Qualifications: college or university degree, Body mass index, Whole body fat mass, Body fat percentage, Trunk fat mass, Trunk fat percentage
	rs16841904[a]	Worrier or anxious feelings
	rs13136827[a]	Self-reported malabsorption or celiac disease
	rs9271858[a]	Self-reported malabsorption or celiac disease
	rs2516440[a]	Self-reported malabsorption or celiac disease
	rs9271255[a]	Self-reported malabsorption or celiac disease
	rs6920220[a]	Self-reported malabsorption or celiac disease
	rs1182188[ab]	Weight, Trunk fat mass, Whole body fat mass, Trunk fat percentage, Body fat percentage, Psoriasis
	rs7547569[ab]	Psoriasis, Chronic inflammatory diseases (psoriasis)
	rs1990760[ab]	Self-reported hypothyroidism or myxoedema, Psoriasis
	rs1517352[a]	Self-reported hypothyroidism or myxoedema
	rs4947328[a]	Self-reported hypothyroidism or myxoedema
	rs10748783[a]	Self-reported hypothyroidism or myxoedema
	rs12132349[a]	Celiac disease
	rs12720356[ab]	Psoriasis, Chronic inflammatory diseases (psoriasis)

a and b were removed in the MR analysis for [a]sarcoidosis and [b]PBC during heterogeneity test via RadialMR. SNP: single nucleotide polymorphism. All statistical tests were two-sided. A *P*-value<5×10^{-8} with a potential confounder in the PhenoScanner database was considered genome-wide significant and removed.

Supplementary Table 4. Summary information on Sarcoidosis for the 127 genome-wide significant SNPs associated with IBD

SNP	A1	A2	EAF	Bata	SE	*P*-value
rs72634258	T	C	0.253841	-0.0252371	0.0235889	0.284677
rs7523442	C	T	0.491249	0.0257035	0.0205049	0.210014
rs6588248	T	G	0.498475	-0.0918018	0.0204743	7.33432E-06
rs7547569	T	C	0.0474504	-0.286745	0.0531163	6.72249E-08
rs34856868	G	A	0.0340674	-0.0360062	0.0576575	0.532309
rs2974935	G	T	0.544626	0.00869105	0.0206153	0.67333
rs2297559	G	A	0.680388	0.0131554	0.0219387	0.548744
rs10800309	A	G	0.629822	-0.00935336	0.0212499	0.659821
rs12411259	G	A	0.282303	0.0394107	0.0228176	0.0841298
rs2488397[a]	G	C	0.145891	0.0940615	0.0282473	0.00086868
rs35730213	G	C	0.212473	0.00898135	0.0250124	0.719538
rs3024493	C	A	0.156093	-0.0248295	0.0284559	0.382902
rs13407913[a]	A	G	0.410035	0.0513769	0.0208275	0.0136333
rs780094[a]	T	C	0.644411	-0.0582246	0.0212915	0.00624482
rs78487399	G	C	0.0553268	0.113393	0.0431395	0.00857571
rs7608910	A	G	0.369714	0.093601	0.0209682	8.04656E-06
rs6740462	C	A	0.626115	-0.0103019	0.0211868	0.626794
rs1420098	T	C	0.401392	-0.0345248	0.0208927	0.0984351
rs11691685	A	G	0.0787185	-0.02804	0.03794	0.45987
rs1990760	C	T	0.585137	0.0132563	0.0208369	0.524649
rs1517352	A	C	0.62365	0.0140059	0.0211286	0.507403
rs72924296	A	G	0.226497	-0.0108003	0.0244471	0.658647
rs11677953	G	A	0.306726	-0.00359212	0.0221994	0.871454
rs35256947	T	C	0.216231	0.0633169	0.0244693	0.00966451
rs6708373	A	G	0.438871	0.0392214	0.0206564	0.057597
rs6745185	T	G	0.833743	-0.0243655	0.0274377	0.374525
rs11713774	T	C	0.117482	0.0332698	0.0318016	0.295483
rs9836291[a]	G	A	0.413161	0.00119989	0.0209189	0.954259
rs4692386	T	C	0.621342	0.01249	0.0211842	0.555465
rs13107612	C	T	0.309152	-0.00323981	0.0222074	0.884009
rs974801	A	G	0.383284	0.0281392	0.0210842	0.182005
rs7657746[a]	A	G	0.140089	-0.0954323	0.0300289	0.00148286
rs3776414	T	G	0.29826	0.0323532	0.0223885	0.148435
rs4703855	C	T	0.36576	0.011228	0.021338	0.598752
rs34804116	C	A	0.365608	0.0133404	0.0212336	0.529828
rs1363907	G	A	0.378083	0.00570123	0.0211388	0.787388
rs272882	G	T	0.585109	-0.0153076	0.0208738	0.463349
rs181826	C	A	0.657353	0.0530202	0.0215854	0.0140375
rs71593329	T	G	0.152101	-0.0270761	0.0284104	0.340572
rs36048684	T	A	0.0701232	-0.0675043	0.0402203	0.093276
rs56167332	C	A	0.30515	0.0856818	0.0219464	9.45649E-05
rs4976646	T	C	0.359918	0.0328268	0.0213436	0.124044
rs7773324	G	A	0.553953	-0.0185245	0.0206371	0.369384

rs1267499[a]	T	C	0.887695	0.0287843	0.0322747	0.372471
rs2328546	T	C	0.756043	0.0532844	0.0239246	0.0259352
rs6456426	C	A	0.486929	-0.0105562	0.0204814	0.606268
rs9264942[a]	T	C	0.27498	-0.0379179	0.0229484	0.0984714
rs769177	C	T	0.0563046	-0.140679	0.0472334	0.00289781
rs1847472	C	A	0.24978	0.0361919	0.0236157	0.125391
rs11152949	A	G	0.246566	-0.00678753	0.0237244	0.774802
rs13204742	G	T	0.130052	0.0295191	0.0306572	0.33561
rs6933404[a]	T	C	0.189299	0.0618163	0.0260372	0.0175894
rs62434177	G	A	0.057578	-0.0189229	0.0448614	0.673164
rs9457247	C	T	0.488381	0.00283502	0.0204887	0.889948
rs1182188[a]	T	C	0.360459	0.0161908	0.0214097	0.449508
rs3801835	C	T	0.277308	-0.0216159	0.0229378	0.346002
rs12718244	G	A	0.293313	-0.00873503	0.0225034	0.697893
rs11768997	G	A	0.0562431	-0.0071103	0.0449008	0.874177
rs2395022	A	C	0.955469	-0.0380235	0.0495538	0.442892
rs6466198	A	T	0.630074	0.0111178	0.0212232	0.60038
rs2538470	A	G	0.61892	0.00860245	0.0211454	0.684138
rs7011507	G	A	0.084219	0.0161019	0.0367879	0.661607
rs7015630	T	C	0.270477	0.0399391	0.0231077	0.0839189
rs10956252	C	G	0.723903	-0.0207116	0.022924	0.366267
rs6651252	T	C	0.115412	-0.0821336	0.0323592	0.0111429
rs10758669	C	A	0.6416	-0.0772559	0.0211201	0.000254249
rs4743820	C	T	0.66656	-0.0479502	0.0217761	0.0276681
rs7848647	T	C	0.694117	0.0104167	0.0222615	0.639839
rs11793497	A	G	0.416202	-0.0272465	0.0207524	0.189206
rs12722515	C	A	0.143429	-0.0641295	0.0291331	0.0277172
rs2050392	G	A	0.587746	-0.0012052	0.0208438	0.953892
rs34779708	T	G	0.372102	0.0436218	0.0212422	0.0400203
rs2153283	C	A	0.236578	-0.0018904	0.0240728	0.937408
rs10761659	A	G	0.528353	0.0109078	0.0205239	0.595095
rs2688608	G	T	0.469377	0.0232299	0.0205723	0.258822
rs1250566	G	A	0.409241	0.0405127	0.0209417	0.0530457
rs11185982	T	C	0.122799	0.0236395	0.0314037	0.451593
rs2497318	C	T	0.450475	-0.0551671	0.0205816	0.00735343
rs6584281	A	G	0.529029	0.0413662	0.0205251	0.0438632
rs2274351	C	T	0.523351	-0.0100064	0.0205272	0.625925
rs12796489	C	A	0.00421381	-0.0247948	0.157071	0.874569
rs11230563	C	T	0.24804	-0.069153	0.0240595	0.00404977
rs559928	T	C	0.802538	0.0330168	0.025811	0.200835
rs11236797	C	A	0.405782	-0.00894698	0.0208638	0.668049
rs648541	A	G	0.392963	0.026332	0.0209721	0.20927
rs1388585	G	A	0.973263	-0.0884624	0.0641707	0.168034
rs10878302	T	A	0.0603244	-0.0269581	0.0429973	0.530678
rs12318183	C	A	0.322888	-0.0356938	0.0219106	0.103298
rs12585310	G	A	0.321298	0.00694239	0.0220261	0.752619
rs941823	T	C	0.729023	-0.0220711	0.0230607	0.338524
rs6561151	G	A	0.200043	-0.00966587	0.0256975	0.706812
rs9557207	A	G	0.131925	0.0524336	0.0300063	0.0805638

rs10142466	A	G	0.486983	-0.0332962	0.0204879	0.104129
rs1569328	C	T	0.178519	0.0292579	0.0268599	0.27603
rs55808324	G	A	0.0826126	0.0659241	0.0370788	0.0754119
rs17651741	G	A	0.198664	0.0288	0.025684	0.26215
rs17293632	C	T	0.261846	0.0199891	0.0233657	0.39228
rs367569	C	T	0.27441	-0.104303	0.0233577	7.98914E-06
rs62037363[a]	T	C	0.413081	-0.0679348	0.0208469	0.00111908
rs6500315	A	G	0.849647	0.0112494	0.028768	0.695769
rs7194886	C	T	0.487727	0.0228479	0.0204949	0.264934
rs2270395	C	T	0.69346	-0.00457393	0.0223517	0.837858
rs11641016	C	G	0.264115	-0.0204216	0.0233723	0.382253
rs9889296	G	A	0.336495	0.0285301	0.021707	0.188737
rs4795397	A	G	0.517572	-0.0511614	0.0205032	0.0125855
rs744166	A	G	0.419712	-0.063561	0.0207313	0.0021698
rs3853824	T	C	0.559289	-0.0305274	0.0206823	0.139939
rs1292053	A	G	0.433906	0.0138366	0.0206806	0.503455
rs17780256	A	C	0.170424	0.00674682	0.0272982	0.80479
rs2847278	C	T	0.853676	-0.0502102	0.0289595	0.0829526
rs7240004	A	G	0.37865	0.038344	0.0211959	0.0704466
rs67643815	G	T	0.585139	-0.076594	0.0206839	0.000213
rs2024092	G	A	0.232631	0.0223001	0.0244183	0.361109
rs35164067	G	A	0.17645	-0.0460427	0.0268474	0.0863495
rs7253253	G	T	0.941531	0.100393	0.044266	0.0233319
rs17694108	G	A	0.348264	-0.000175051	0.0215292	0.993513
rs516246[a]	C	T	0.37512	0.0584841	0.020961	0.00526866
rs6111031	C	T	0.124411	-0.00756905	0.0311614	0.808084
rs6058869	C	T	0.40135	0.0161551	0.0208863	0.439241
rs6074022	C	T	0.726719	-0.0326017	0.0230176	0.156663
rs913678	T	C	0.348491	-0.0740415	0.0216964	0.000643398
rs259964	A	G	0.578445	-0.0649199	0.0206535	0.00167063
rs6062496	G	A	0.645495	-0.0203656	0.0214128	0.341556
rs1297258	C	T	0.405233	-0.0406093	0.0208308	0.0512377
rs2836883	G	A	0.246985	-0.0026507	0.0238081	0.91135
rs140143	G	C	0.150237	-0.0488054	0.0287418	0.0894953
rs2143178	T	C	0.117446	-0.0131582	0.03186	0.679607

a SNPs associated with potential confounders. SNP: single nucleotide polymorphism; A1: effect allele; A2: baseline allele; EAF: effect allele frequency; SE: standard error. All statistical tests were two-sided. SNPs rs9273363 was not available in the outcome GWAS, thus, rs9273363 was removed in the MR analysis. A *P*-value$<5\times10^{-8}$ (rs3184504 and rs6933404) was considered genome-wide significant and should be excluded to meet the assumption that requires instruments to be associated with the outcome only through exposure.

Supplementary Table 5. Summary information on Sarcoidosis for the 117 genome-wide significant SNPs associated with CD

SNP	A1	A2	EAF	Bata	SE	*P*-value
rs36016881	A	G	0.265397	-0.035848	0.0232588	0.123253
rs7517847	T	G	0.44154	-0.0625886	0.0206484	0.00243618
rs17129991	C	T	0.0182656	-0.186012	0.0773909	0.0162372
rs17391694[a]	C	T	0.132355	0.0553794	0.0302198	0.0668683
rs6679677	C	A	0.146169	-0.00128218	0.0290191	0.964758
rs2641348	A	G	0.149085	-0.015185	0.0288397	0.59852
rs2974935	G	T	0.544626	0.00869105	0.0206153	0.67333
rs10800309	A	G	0.629822	-0.00935336	0.0212499	0.659821
rs12411259	G	A	0.282303	0.0394107	0.0228176	0.0841298
rs10798069	G	T	0.530256	-0.000772789	0.0205608	0.970018
rs6702421[a]	C	T	0.16827	0.0848688	0.0267579	0.0015153
rs35730213	G	C	0.212473	0.00898135	0.0250124	0.719538
rs3024505	G	A	0.157284	-0.0226129	0.0283441	0.424987
rs13407913[a]	A	G	0.410035	0.0513769	0.0208275	0.0136333
rs780094[a]	T	C	0.644411	-0.0582246	0.0212915	0.00624482
rs77981966	C	T	0.0330623	0.15281	0.0541652	0.00478487
rs7608910	A	G	0.369714	0.093601	0.0209682	8.04656E-06
rs6740462	C	A	0.626115	-0.0103019	0.0211868	0.626794
rs13001325	C	T	0.329679	-0.0307445	0.0217306	0.157127
rs11691685	A	G	0.0787185	-0.02804	0.03794	0.45987
rs1517352	A	C	0.62365	0.0140059	0.0211286	0.507403
rs6738394	G	A	0.376725	-0.00812782	0.021164	0.700948
rs12694846	A	G	0.217101	0.0648617	0.0243899	0.00782889
rs6738490	T	C	0.438941	0.0399108	0.020659	0.053374
rs35320439	T	C	0.235959	0.0353648	0.0242738	0.145141
rs11713774	T	C	0.117482	0.0332698	0.0318016	0.295483
rs3197999[a]	G	A	0.392858	-0.00200798	0.0210772	0.924102
rs7438704	A	G	0.56905	-0.0182585	0.0206354	0.376258
rs34592089	G	A	0.00794512	0.0548863	0.11378	0.629529
rs6827756	T	C	0.532076	-0.00493087	0.0205252	0.810148
rs3776414	T	G	0.29826	0.0323532	0.0223885	0.148435
rs71624119	G	A	0.168286	-0.0439528	0.0274763	0.109673
rs4703855	C	T	0.36576	0.011228	0.021338	0.598752
rs34804116	C	A	0.365608	0.0133404	0.0212336	0.529828
rs1363907	G	A	0.378083	0.00570123	0.0211388	0.787388
rs17622378	A	G	0.317469	0.0118112	0.0220056	0.591449
rs181826	C	A	0.657353	0.0530202	0.0215854	0.0140375
rs11167518	C	A	0.0844714	-0.0406134	0.0368775	0.270764
rs17388425	C	G	0.849542	-0.0213713	0.0285596	0.454276
rs56163845	A	G	0.41353	-0.0465939	0.0208929	0.0257389
rs7773324	G	A	0.553953	-0.0185245	0.0206371	0.369384
rs1267501[a]	T	C	0.887082	0.0318277	0.0321303	0.32189
rs6908425	T	C	0.73881	0.0636747	0.0235352	0.00681993
rs6456426	C	A	0.486929	-0.0105562	0.0204814	0.606268
rs9264942[a]	T	C	0.27498	-0.0379179	0.0229484	0.0984714

rs438475	G	A	0.0843806	0.0735636	0.0367984	0.0455974
rs1847472	C	A	0.24978	0.0361919	0.0236157	0.125391
rs11152949	A	G	0.246566	-0.00678753	0.0237244	0.774802
rs9491892	T	G	0.149791	0.0424176	0.0289207	0.142462
rs9494844	C	A	0.328924	0.0178417	0.0218067	0.413257
rs212388[a]	C	T	0.662954	0.0154069	0.0216232	0.476146
rs9457247	C	T	0.488381	0.00283502	0.0204887	0.889948
rs3801810	G	A	0.187239	-0.0362966	0.0262402	0.16659
rs7786444	C	T	0.0713945	-0.0231724	0.0397512	0.559937
rs1456896	C	T	0.71207	0.0779274	0.0228176	0.000637294
rs11768997	G	A	0.0562431	-0.0071103	0.0449008	0.874177
rs2395022	A	C	0.955469	-0.0380235	0.0495538	0.442892
rs2538470	A	G	0.61892	0.00860245	0.0211454	0.684138
rs7015630	T	C	0.270477	0.0399391	0.0231077	0.0839189
rs10956252	C	G	0.723903	-0.0207116	0.022924	0.366267
rs6651252	T	C	0.115412	-0.0821336	0.0323592	0.0111429
rs10758669	C	A	0.6416	-0.0772559	0.0211201	0.000254249
rs7848647	T	C	0.694117	0.0104167	0.0222615	0.639839
rs11793497	A	G	0.416202	-0.0272465	0.0207524	0.189206
rs61839660	C	T	0.0408556	-0.0657968	0.0513326	0.199922
rs303429	C	T	0.586934	-0.00104185	0.0208018	0.960055
rs34779708	T	G	0.372102	0.0436218	0.0212422	0.0400203
rs2153283	C	A	0.236578	-0.0018904	0.0240728	0.937408
rs10995271	G	C	0.389643	-0.0414	0.0210909	0.0496547
rs2227551	G	T	0.63717	0.00956821	0.0213997	0.65479
rs1250573	G	A	0.365737	0.0288047	0.0213162	0.176597
rs11185982	T	C	0.122799	0.0236395	0.0314037	0.451593
rs7085798	C	A	0.528627	0.0405294	0.020526	0.0483203
rs12796489	C	A	0.00421381	-0.0247948	0.157071	0.874569
rs34787213	C	T	0.0993923	-0.0292689	0.0342724	0.393101
rs559928	T	C	0.802538	0.0330168	0.025811	0.200835
rs11236797	C	A	0.405782	-0.00894698	0.0208638	0.668049
rs28999107	G	T	0.515467	-0.0416211	0.0207666	0.0450454
rs76906269	A	G	0.00674162	0.12068	0.124176	0.331129
rs10878302	T	A	0.0603244	-0.0269581	0.0429973	0.530678
rs7969592	A	G	0.38308	-0.0255783	0.0210647	0.224644
rs1927681	T	A	0.536916	0.0126646	0.0205964	0.538624
rs915286	G	A	0.471872	-0.0114208	0.0205197	0.577817
rs9594766	G	A	0.612976	0.0123493	0.0210667	0.557742
rs6561151	G	A	0.200043	-0.00966587	0.0256975	0.706812
rs9554587	A	G	0.132456	0.0539579	0.0299685	0.0717844
rs1569328	C	T	0.178519	0.0292579	0.0268599	0.27603
rs11159833	C	T	0.0811251	0.0545399	0.0373869	0.144621
rs72727394	C	T	0.205444	0.0203417	0.0253554	0.422401
rs17293632	C	T	0.261846	0.0199891	0.0233657	0.39228
rs1646019	C	T	0.30057	-0.0939674	0.0226258	0.000032799
rs26528[a]	T	C	0.530352	-0.0603676	0.0204491	0.00315624
rs6500315	A	G	0.849647	0.0112494	0.028768	0.695769
rs7194886	C	T	0.487727	0.0228479	0.0204949	0.264934

rs2270395	C	T	0.69346	-0.00457393	0.0223517	0.837858
rs11117431	A	G	0.264145	-0.0206569	0.0233734	0.376817
rs9889296	G	A	0.336495	0.0285301	0.021707	0.188737
rs4795397	A	G	0.517572	-0.0511614	0.0205032	0.0125855
rs12949918	T	C	0.419757	-0.0641049	0.0207301	0.00198573
rs3853824	T	C	0.559289	-0.0305274	0.0206823	0.139939
rs1292053	A	G	0.433906	0.0138366	0.0206806	0.503455
rs2847293	A	T	0.847607	-0.063981	0.0284219	0.0243781
rs7236492	C	T	0.199459	0.0219748	0.025741	0.393277
rs2024092	G	A	0.232631	0.0223001	0.0244183	0.361109
rs35164067	G	A	0.17645	-0.0460427	0.0268474	0.0863495
rs17694108	G	A	0.348264	-0.000175051	0.0215292	0.993513
rs640466	T	C	0.369912	-0.0101108	0.0212014	0.633439
rs516246[a]	C	T	0.37512	0.0584841	0.020961	0.00526866
rs6111031	C	T	0.124411	-0.00756905	0.0311614	0.808084
rs6074022	C	T	0.726719	-0.0326017	0.0230176	0.156663
rs259964	A	G	0.578445	-0.0649199	0.0206535	0.00167063
rs6062496	G	A	0.645495	-0.0203656	0.0214128	0.341556
rs1297258	C	T	0.405233	-0.0406093	0.0208308	0.0512377
rs2284553	A	G	0.629997	0.0624867	0.0213257	0.00338836
rs140143	G	C	0.150237	-0.0488054	0.0287418	0.0894953
rs2413583	C	T	0.117111	-0.0131963	0.0318956	0.679069
rs727563[a]	C	T	0.733931	0.0218008	0.0231607	0.346558

a SNPs associated with potential confounders. SNP: single nucleotide polymorphism; A1: effect allele; A2: baseline allele; EAF: effect allele frequency; SE: standard error. All statistical tests were two-sided. SNPs rs212388 was not available in the outcome GWAS, thus, rs212388 was removed in the MR analysis. A *P*-value$<5\times10^{-8}$ (rs3184504) was considered genome-wide significant and should be excluded to meet the assumption that requires instruments to be associated with the outcome only through exposure.

Supplementary Table 6. Summary information on Sarcoidosis for the 82 genome-wide significant SNPs associated with UC

SNP	A1	A2	EAF	Bata	SE	*P*-value
rs10910092	A	G	0.475457	-0.0216405	0.0205253	0.29173
rs35223180	G	T	0.254942	-0.0261093	0.0235478	0.267527
rs6426833	G	A	0.508795	0.024649	0.0204892	0.228968
rs111830527	G	A	0.0122732	-0.0540017	0.0920266	0.557335
rs7547569[a]	T	C	0.0474504	-0.286745	0.0531163	6.72249E-08
rs4656958	A	G	0.680416	0.0121312	0.0219306	0.580152
rs1801274	A	G	0.50333	-0.0332673	0.0204914	0.104487
rs16841904[a]	C	T	0.145898	0.0940803	0.0282452	0.000865845
rs12132349[a]	T	A	0.212497	0.00891109	0.025002	0.721529
rs3024493	C	A	0.156093	-0.0248295	0.0284559	0.382902
rs10460566	G	A	0.823844	-0.021398	0.0268542	0.425554
rs13430791	G	A	0.0713395	0.090643	0.0395968	0.0220709
rs7608910	A	G	0.369714	0.093601	0.0209682	8.04656E-06
rs10185424	T	G	0.698795	-0.00333254	0.0224641	0.882067
rs1990760[a]	C	T	0.585137	0.0132563	0.0208369	0.524649
rs1517352[a]	A	C	0.62365	0.0140059	0.0211286	0.507403
rs9941524	A	G	0.532034	0.000840695	0.0205124	0.967308
rs11676348	C	T	0.407672	0.000534763	0.0208621	0.97955
rs4973341	C	T	0.770813	-0.0176985	0.0243693	0.467678
rs4676410	G	A	0.270901	-0.00404146	0.0231153	0.861206
rs9836291[a]	G	A	0.413161	0.00119989	0.0209189	0.954259
rs3774937	T	C	0.348267	0.0640988	0.0213293	0.00265406
rs13136827[a]	T	C	0.113844	-0.106946	0.0329988	0.00119149
rs3776414	T	G	0.29826	0.0323532	0.0223885	0.148435
rs272882	G	T	0.585109	-0.0153076	0.0208738	0.463349
rs36070529	G	A	0.152101	-0.0270686	0.0284103	0.340705
rs56167332	C	A	0.30515	0.0856818	0.0219464	9.45649E-05
rs4976646	T	C	0.359918	0.0328268	0.0213436	0.124044
rs4712520	T	C	0.786731	0.0315499	0.0250175	0.207269
rs7738430	T	C	0.0530174	-0.130185	0.0483631	0.00710624
rs4947328[a]	A	G	0.0460554	-0.0982495	0.048676	0.0435452
rs34659678	C	T	0.0632951	-0.0168202	0.0420626	0.689241
rs6920220[a]	G	A	0.189505	0.0601449	0.0259968	0.0206924
rs1182188[a]	T	C	0.360459	0.0161908	0.0214097	0.449508
rs1077773	G	A	0.51037	0.0323666	0.0205516	0.115282
rs12718244	G	A	0.293313	-0.00873503	0.0225034	0.697893
rs76546301	G	A	0.0123415	-0.015855	0.0923911	0.863746
rs2395022	A	C	0.955469	-0.0380235	0.0495538	0.442892
rs6466198	A	T	0.630074	0.0111178	0.0212232	0.60038
rs4728142	G	A	0.420839	0.00983636	0.0207448	0.635387
rs13255292	C	T	0.342858	5.23955E-05	0.0216396	0.998068
rs10758669	C	A	0.6416	-0.0772559	0.0211201	0.000254249
rs4743820	C	T	0.66656	-0.0479502	0.0217761	0.0276681
rs4366152	T	C	0.707323	0.00776521	0.0225359	0.730417
rs10870077	C	G	0.416959	-0.0276532	0.0207443	0.182514

rs4747886	C	T	0.398274	0.0501357	0.0209531	0.0167221
rs59418206	G	A	0.372892	0.0444769	0.0212299	0.0361693
rs10761659	A	G	0.528353	0.0109078	0.0205239	0.595095
rs2497318	C	T	0.450475	-0.0551671	0.0205816	0.00735343
rs10748783[a]	C	A	0.529026	0.0413341	0.0205253	0.0440301
rs2274351	C	T	0.523351	-0.0100064	0.0205272	0.625925
rs12796489	C	A	0.00421381	-0.0247948	0.157071	0.874569
rs11229555	G	T	0.277942	-0.00645766	0.0229703	0.77861
rs11230563	C	T	0.24804	-0.069153	0.0240595	0.00404977
rs61893460	G	A	0.406025	-0.00785821	0.0208685	0.706501
rs483905	G	A	0.214924	0.0125348	0.0249329	0.615144
rs661054	A	G	0.392885	0.0251955	0.02097	0.229556
rs76904798	C	T	0.0904682	-0.0407726	0.0357959	0.25469
rs12318183	C	A	0.322888	-0.0356938	0.0219106	0.103298
rs1927681	T	A	0.536916	0.0126646	0.0205964	0.538624
rs941823	T	C	0.729023	-0.0220711	0.0230607	0.338524
rs55808324	G	A	0.0826126	0.0659241	0.0370788	0.0754119
rs11641184	C	A	0.472815	0.000351132	0.0205464	0.986365
rs7404095	T	C	0.636672	-0.00296497	0.0213758	0.889682
rs11150589	T	C	0.565214	0.057063	0.0208885	0.00629898
rs79045992	G	A	0.080848	0.0712958	0.0376348	0.0581701
rs4795397	A	G	0.517572	-0.0511614	0.0205032	0.0125855
rs9891119	A	C	0.381658	-0.0566161	0.0211169	0.00733838
rs17780256	A	C	0.170424	0.00674682	0.0272982	0.80479
rs8096327	A	G	0.347398	0.0321796	0.0215418	0.135223
rs7240004	A	G	0.37865	0.038344	0.0211959	0.0704466
rs12720356[a]	A	C	0.0818822	-0.0708934	0.0374126	0.0581045
rs17694108	G	A	0.348264	-0.000175051	0.0215292	0.993513
rs11083840	T	G	0.342508	0.0232248	0.0215565	0.281307
rs6111031	C	T	0.124411	-0.00756905	0.0311614	0.808084
rs4812833	G	A	0.589613	0.00380996	0.0209698	0.855828
rs913678	T	C	0.348491	-0.0740415	0.0216964	0.000643398
rs6062496	G	A	0.645495	-0.0203656	0.0214128	0.341556
rs1297256	C	T	0.405216	-0.0396174	0.0208277	0.0571518
rs2836883	G	A	0.246985	-0.0026507	0.0238081	0.91135
rs140143	G	C	0.150237	-0.0488054	0.0287418	0.0894953
rs9611131	T	C	0.11822	-0.0184021	0.0318179	0.563023

a SNPs associated with potential confounders. SNP: single nucleotide polymorphism; A1: effect allele; A2: baseline allele; EAF: effect allele frequency; SE: standard error. All statistical tests were two-sided. SNPs rs9271858 was not available in the outcome GWAS, thus, rs9271858 was removed in the MR analysis. A *P*-value$<5\times10^{-8}$ (rs2516440 and rs9271255) was considered genome-wide significant and should be excluded to meet the assumption that requires instruments to be associated with the outcome only through exposure.

Supplementary Table 7. Summary information on sarcoidosis SNPs used as genetic instruments for the Mendelian randomization analyses

SNP	A1	A2	EAF	Bata	SE	N	*P*-value	R^2	F
**61 SNPs for sarcoidosis**									
rs78343895	C	T	0.00160316	0.921637	0.194997	451377	2.29E-06	4.95E-05	22.33891
rs186757051	C	G	0.00969415	0.499032	0.0918595	451377	5.56E-08	6.54E-05	29.51255
rs2024825	T	C	0.835847	-0.215536	0.0263021	451377	2.51E-16	1.49E-04	67.15168
rs4845339	C	G	0.459054	-0.0982658	0.0205838	451377	1.81E-06	5.05E-05	22.79039
rs190232996	C	T	0.00208706	-1.75538	0.384276	451377	4.92E-06	4.62E-05	20.8667
rs150824635	G	T	0.00889996	0.464269	0.0952494	451377	1.09E-06	5.26E-05	23.75817
rs572145962	C	A	0.0067645	0.500606	0.10634	451377	2.51E-06	4.91E-05	22.16138
rs12062708	A	G	0.03867	0.251698	0.0503108	451377	5.65E-07	5.54E-05	25.02852
rs10181042	C	T	0.39637	0.107709	0.0207472	451377	2.09E-07	5.97E-05	26.95151
rs181926316	C	G	0.0205432	0.394525	0.0721684	451377	4.58E-08	6.62E-05	29.88498
rs182482858	T	C	0.0196618	0.435571	0.065039	451377	2.13E-11	9.94E-05	44.8506
rs11677465	A	G	0.204829	-0.139497	0.0260103	451377	8.18E-08	6.37E-05	28.76319
rs79269824	C	T	0.00181738	0.867959	0.181282	451377	1.69E-06	5.08E-05	22.92383
rs35202091	C	T	0.278896	0.136968	0.0222859	451377	7.95E-10	8.37E-05	37.77252
rs17041517	G	A	0.589938	-0.101765	0.0208296	451377	1.03E-06	5.29E-05	23.86894
rs151201033	G	A	0.0434723	-0.285292	0.0563188	451377	4.07E-07	5.68E-05	25.66082
rs62263866	C	T	0.0335228	-0.290761	0.0626284	451377	3.44E-06	4.77E-05	21.55399
rs4444827	T	C	0.760811	-0.109406	0.0234646	451377	3.12E-06	4.82E-05	21.73972
rs11567997	C	G	0.0978122	0.160892	0.0330875	451377	1.16E-06	5.24E-05	23.64499
rs145903485	C	T	0.00208595	0.826741	0.175677	451377	2.53E-06	4.91E-05	22.14661
rs186833602	A	T	0.00172957	0.937733	0.186643	451377	5.06E-07	5.59E-05	25.24254
rs72836177	T	C	0.145039	-0.138352	0.0299472	451377	3.84E-06	4.73E-05	21.34305
rs9467728	C	T	0.179932	-0.193706	0.0278689	451377	3.64E-12	1.07E-04	48.31084
rs7753218	C	T	0.342681	-0.277885	0.0220953	451377	2.84E-36	3.50E-04	158.17159
rs67497288	G	A	0.463448	-0.484073	0.0214761	451377	1.68E-112	1.12E-03	508.05293
rs9274504	C	T	0.250193	0.21875	0.0227162	451377	5.99E-22	2.05E-04	92.73055
rs1431403	T	C	0.24481	0.307686	0.0224053	451377	6.46E-43	4.18E-04	188.58712
rs12663194	C	A	0.276349	0.127065	0.0224077	451377	1.42E-08	7.12E-05	32.15551
rs542273383	G	C	0.0276595	0.268742	0.0587631	451377	4.80E-06	4.63E-05	20.91509
rs149256681	T	C	0.0376542	0.262043	0.0509555	451377	2.71E-07	5.86E-05	26.44607
rs873973	C	T	0.199055	0.123334	0.0250439	451377	8.45E-07	5.37E-05	24.25268
rs17168021	T	C	0.325133	0.10195	0.0215406	451377	2.21E-06	4.96E-05	22.40046
rs147191825	C	T	0.00366655	-1.25332	0.250215	451377	5.47E-07	5.56E-05	25.08969
rs3133595	C	T	0.679321	0.107459	0.0222588	451377	1.38E-06	5.16E-05	23.30667
rs148811994	T	A	0.0196551	-0.393687	0.0852928	451377	3.92E-06	4.72E-05	21.3047
rs12683452	A	G	0.448557	0.101875	0.020483	451377	6.57E-07	5.48E-05	24.73695
rs12377541	C	T	0.0809262	0.164259	0.0358614	451377	4.64E-06	4.65E-05	20.97983
rs528029164	A	T	0.00127687	-3.37054	0.736302	451377	4.70E-06	4.64E-05	20.95488
rs1079242	A	G	0.560234	-0.239037	0.0204009	451377	1.04E-31	3.04E-04	137.28708
rs74230546	A	C	0.245939	-0.113555	0.0241678	451377	2.62E-06	4.89E-05	22.07681
rs663743	G	A	0.376025	-0.18195	0.02141	451377	1.92E-17	1.60E-04	72.22189
rs12296430	G	C	0.199631	-0.1438	0.0267019	451377	7.23E-08	6.42E-05	29.00224
rs4764279	G	A	0.91718	0.178798	0.0390068	451377	4.57E-06	4.65E-05	21.01081

rs148781286	G	C	0.00584381	-0.98071	0.184335	451377	1.04E-07	6.27E-05	28.30503
rs4766578	T	A	0.57044	-0.150469	0.0204831	451377	2.04E-13	1.20E-04	53.96358
rs7327898	C	T	0.153794	0.12707	0.0274667	451377	3.72E-06	4.74E-05	21.40283
rs9601381	G	A	0.166132	-0.13396	0.0282563	451377	2.13E-06	4.98E-05	22.47594
rs57226083	G	A	0.307207	0.105313	0.0220113	451377	1.71E-06	5.07E-05	22.89131
rs190649695	G	A	0.0108524	0.419306	0.0878751	451377	1.83E-06	5.04E-05	22.76819
rs181956065	T	C	0.0854164	-0.184057	0.0386759	451377	1.95E-06	5.02E-05	22.64758
rs149126014	G	A	0.0187598	0.313676	0.0684872	451377	4.65E-06	4.65E-05	20.97693
rs59199978	A	G	0.206405	-0.122988	0.0258722	451377	2.00E-06	5.01E-05	22.59731
rs3760112	C	T	0.126198	-0.159303	0.0321604	451377	7.29E-07	5.44E-05	24.53596
rs11645302	C	T	0.286408	0.112292	0.0223655	451377	5.15E-07	5.58E-05	25.20801
rs9930621	T	C	0.111782	-0.158238	0.0339329	451377	3.11E-06	4.82E-05	21.74592
rs16969084	G	A	0.00276567	-1.60741	0.314013	451377	3.07E-07	5.80E-05	26.20331
rs8084448	T	C	0.728355	-0.106518	0.0229171	451377	3.35E-06	4.79E-05	21.60353
rs34536443	G	C	0.0302097	-0.405918	0.067444	451377	1.76E-09	8.02E-05	36.2233
rs73109224	A	G	0.0115935	-0.510894	0.110398	451377	3.70E-06	4.74E-05	21.41595
rs1860217	G	C	0.419008	0.12911	0.020608	451377	3.73E-10	8.70E-05	39.25059
rs2157739	G	A	0.131857	0.122132	0.0240737	451377	3.91E-07	5.70E-05	25.73779

SNP: single nucleotide polymorphism; A1: effect allele; A2: baseline allele; EAF: effect allele frequency; SE: standard error. N refers to the sample size of the initial GWAS from which the genetic variants were selected. All statistical tests were two-sided. A *P*-value$<5\times10^{-6}$ was considered genome-wide significant.

Supplementary Table 8. Summary information on IBD for the 3 genome-wide significant SNPs associated with Sarcoidosis

SNP	A1	A2	EAF	Bata	SE	*P*-value
rs12296430	C	G	0.1932	-0.0201468	0.0130758	0.123373
rs12663194	A	C	0.3111	-0.0134985	0.0111516	0.226103

SNP: single nucleotide polymorphism; A1: effect allele; A2: baseline allele; EAF: effect allele frequency; SE: standard error. All statistical tests were two-sided. A *P*-value<5×10^{-8} was considered genome-wide significant and should be excluded to meet the assumption that requires instruments to be associated with the outcome only through exposure.

Supplementary Table 9. Summary information on CD for the 3 genome-wide significant SNPs associated with Sarcoidosis

SNP	A1	A2	EAF	Bata	SE	*P*-value
rs12296430	C	G	0.1932	-0.0343208	0.0156291	0.0280951
rs3760112	T	C	0.09882	-0.048965	0.0205112	0.0169762

SNP: single nucleotide polymorphism; A1: effect allele; A2: baseline allele; EAF: effect allele frequency; SE: standard error. All statistical tests were two-sided. A *P*-value<5×10^{-8} was considered genome-wide significant and should be excluded to meet the assumption that requires instruments to be associated with the outcome only through exposure.

Supplementary Table 10. Summary information on UC for the 4 genome-wide significant SNPs associated with Sarcoidosis

SNP	A1	A2	EAF	Bata	SE	*P*-value
rs12296430	C	G	0.1932	-0.00791669	0.0165815	0.633049
rs12663194	A	C	0.3111	-0.00685223	0.013996	0.624429
rs2024825	C	T	0.8339	-0.0221947	0.0168816	0.188601
rs35202091	T	C	0.3109	0.00957679	0.0138566	0.489479
rs3760112	T	C	0.09882	-0.0688963	0.0215139	0.00136286

SNP: single nucleotide polymorphism; A1: effect allele; A2: baseline allele; EAF: effect allele frequency; SE: standard error. All statistical tests were two-sided. A *P*-value<5×10^{-8} was considered genome-wide significant and should be excluded to meet the assumption that requires instruments to be associated with the outcome only through exposure.

Supplementary Table 11. Summary information on PBC for the 86 genome-wide significant SNPs associated with IBD

SNP	A1	A2	EAF	Bata	SE	*P*-value
rs7657746	G	A	*N/A*	-0.0143224	0.0189373	4.49E-01
rs780094[b]	C	T	*N/A*	-0.0878294	0.0219722	6.41E-05
rs2974935	T	G	*N/A*	-0.0153651	0.0217876	4.81E-01
rs1990760	T	C	*N/A*	-0.0799991	0.0219391	2.66E-04
rs1420098	C	T	*N/A*	-0.00417007	0.0211905	8.44E-01
rs7547569	C	T	*N/A*	0.0861795	0.0457405	5.96E-02
rs9264942	C	T	*N/A*	-0.000236166	0.0228934	9.92E-01
rs3776414	G	T	*N/A*	0.0426516	0.0222792	5.56E-02
rs181826	A	C	*N/A*	0.116303	0.0225169	2.40E-07
rs11768997	A	G	*N/A*	0.00776614	0.0934196	9.34E-01
rs12722515	A	C	*N/A*	-0.0817738	0.0303893	7.13E-03
rs6058869	T	C	*N/A*	-0.00462077	0.0203796	8.21E-01
rs2153283	A	C	*N/A*	0.0261288	0.0265575	3.25E-01
rs1388585	A	G	*N/A*	-0.103348	0.0865042	2.32E-01
rs11677953	A	G	*N/A*	0.0558512	0.0218166	1.05E-02
rs13107612	T	C	*N/A*	-0.0681765	0.0236991	4.02E-03
rs2497318	T	C	*N/A*	0.0229023	0.0222374	3.03E-01
rs10758669	A	C	*N/A*	-0.0320772	0.022662	1.57E-01
rs4703855	T	C	*N/A*	-0.0272157	0.0238445	2.54E-01
rs9457247	T	C	*N/A*	0.0471592	0.0215914	2.90E-02
rs2395022	C	A	*N/A*	-0.148553	0.0564041	8.45E-03
rs11691685	G	A	*N/A*	-0.0302678	0.0377908	4.23E-01
rs13407913[b]	G	A	*N/A*	0.0715351	0.0223435	1.37E-03
rs7608910	G	A	*N/A*	0.0403862	0.0223608	7.09E-02
rs559928	C	T	*N/A*	0.101017	0.0285056	3.94E-04
rs79980175	C	A	*N/A*	0.0304887	0.0333427	3.61E-01
rs6111031	T	C	*N/A*	-0.0178831	0.0317653	5.73E-01
rs941823	C	T	*N/A*	-0.0340812	0.0252548	1.77E-01
rs62037363[b]	C	T	*N/A*	-0.000103036	0.0245638	9.97E-01
rs10800309	G	A	*N/A*	0.0256305	0.0228565	2.62E-01
rs974801	G	A	*N/A*	-0.00285844	0.022356	8.98E-01
rs3024493	A	C	*N/A*	0.133212	0.0283991	2.72E-06
rs2328546	C	T	*N/A*	0.00835173	0.027201	7.59E-01
rs2688608	T	G	*N/A*	0.0739873	0.0224807	9.98E-04
rs11185982	C	T	*N/A*	0.0424091	0.0309218	1.70E-01
rs1182188	C	T	*N/A*	0.0176448	0.0238702	4.60E-01
rs12318183	A	C	*N/A*	-0.066656	0.0228903	3.59E-03
rs6584281	G	A	*N/A*	-0.00286691	0.0208766	8.91E-01

rs3853824	C	T	*N/A*	0.0227164	0.0231198	3.26E-01
rs17293632	T	C	*N/A*	0.05234	0.0254438	3.97E-02
rs12718244	A	G	*N/A*	-0.016444	0.0215983	4.46E-01
rs17780256	C	A	*N/A*	-0.0117797	0.0280071	6.74E-01
rs78487399	C	G	*N/A*	0.00953743	0.0349048	7.85E-01
rs6740462	A	C	*N/A*	0.00814001	0.0259303	7.54E-01
rs13204742	T	G	*N/A*	0.0873776	0.0321671	6.60E-03
rs6708373	G	A	*N/A*	0.0437247	0.0221841	4.87E-02
rs9836291[b]	A	G	*N/A*	0.0220202	0.0237831	3.55E-01
rs2836883	A	G	*N/A*	0.0159197	0.026896	5.54E-01
rs10956252	G	C	*N/A*	0.0109544	0.02234	6.24E-01
rs6651252	C	T	*N/A*	-0.0293114	0.031747	3.56E-01
rs11230563	T	C	*N/A*	-0.0349185	0.0216253	1.06E-01
rs744166	G	A	*N/A*	0.0131055	0.0188652	4.87E-01
rs1292053	G	A	*N/A*	0.0726748	0.0216758	8.00E-04
rs7011507	A	G	*N/A*	0.0338432	0.0354403	3.40E-01
rs7848647	C	T	*N/A*	0.106979	0.0230036	3.31E-06
rs769177	T	C	*N/A*	0.079934	0.0700157	2.54E-01
rs6074022	T	C	*N/A*	-0.0957914	0.0240747	6.92E-05
rs2143178	C	T	*N/A*	-0.0193157	0.0289193	5.04E-01
rs12411259	A	G	*N/A*	-0.0533721	0.0249164	3.22E-02
rs6745185	G	T	*N/A*	0.0107852	0.0244459	6.59E-01
rs35256947	C	T	*N/A*	-0.0195453	0.0266636	4.64E-01
rs34804116	A	C	*N/A*	0.0118212	0.0334159	7.24E-01
rs272882	T	G	*N/A*	0.0109851	0.0271274	6.86E-01
rs7711427	C	A	*N/A*	0.00279048	0.0228632	9.03E-01
rs1363907	A	G	*N/A*	0.0752304	0.0218581	5.78E-04
rs1517352	C	A	*N/A*	-0.0953422	0.0218891	1.33E-05
rs10878302	A	T	*N/A*	0.00487217	0.0443393	9.13E-01
rs6561151	A	G	*N/A*	-0.00684871	0.024383	7.79E-01
rs8127691	C	T	*N/A*	-0.0700581	0.021273	9.90E-04
rs2297559	A	G	*N/A*	0.0263362	0.0234751	2.62E-01
rs11713774	C	T	*N/A*	0.0510151	0.0323906	1.15E-01
rs516246[b]	T	C	*N/A*	0.0305411	0.021373	1.53E-01
rs1847472	A	C	*N/A*	0.0180198	0.0223493	4.20E-01
rs9889296	A	G	*N/A*	-0.0446737	0.0251467	7.56E-02
rs55808324	A	G	*N/A*	0.0235854	0.0353592	5.05E-01
rs7523442	T	C	*N/A*	-0.0404559	0.0208602	5.25E-02
rs6466198	T	A	*N/A*	0.0227941	0.0228554	3.19E-01
rs2538470	G	A	*N/A*	-0.0486628	0.0231468	3.55E-02
rs34779708	G	T	*N/A*	0.0494725	0.0212999	2.02E-02

rs2050392	A	G	*N/A*	0.0745738	0.0229228	1.14E-03
rs2274351	C	T	*N/A*	-0.0383923	0.0219316	8.00E-02
rs9557207	G	A	*N/A*	-0.119893	0.027731	1.54E-05
rs17651741	A	G	*N/A*	0.114618	0.026487	1.51E-05
rs2847278	C	T	*N/A*	0.091212	0.028766	1.52E-03
rs1297258	T	C	*N/A*	0.0520673	0.0215502	1.57E-02
rs140143	G	T	*N/A*	-0.084875	0.0222213	1.34E-04

b SNPs associated with potential confounders. SNP: single nucleotide polymorphism; A1: effect allele; A2: baseline allele; EAF: effect allele frequency; SE: standard error. All statistical tests were two-sided. *N/A*, Not available. SNPs rs9273363 was not available in the outcome GWAS, thus, rs9273363 was removed in the MR analysis. A *P*-value$<5\times10^{-8}$ (rs3184504 and rs6933404) was considered genome-wide significant and should be excluded to meet the assumption that requires instruments to be associated with the outcome only through exposure.

Supplementary Table 12. Summary information on PBC for the 84 genome-wide significant SNPs associated with CD

SNP	A1	A2	EAF	Bata	SE	*P*-value
rs780094[b]	C	T	*N/A*	-0.0878294	0.0219722	6.41E-05
rs2974935	T	G	*N/A*	-0.0153651	0.0217876	4.81E-01
rs17622378	G	A	*N/A*	0.0209368	0.0221161	3.44E-01
rs56163845	G	A	*N/A*	-0.0227205	0.0231643	3.27E-01
rs9264942	C	T	*N/A*	-0.00023617	0.0228934	9.92E-01
rs3776414	G	T	*N/A*	0.0426516	0.0222792	5.56E-02
rs438475	A	G	*N/A*	-0.0994914	0.0316853	1.69E-03
rs181826	A	C	*N/A*	0.116303	0.0225169	2.40E-07
rs1456896	T	C	*N/A*	0.00242343	0.0231473	9.17E-01
rs11768997	A	G	*N/A*	0.00776614	0.0934196	9.34E-01
rs3801810	A	G	*N/A*	-0.0575246	0.0249231	2.10E-02
rs303429	T	C	*N/A*	0.0745738	0.0229228	1.14E-03
rs61839660	T	C	*N/A*	0.186692	0.0348451	8.43E-08
rs2153283	A	C	*N/A*	0.0261288	0.0265575	3.25E-01
rs2227551	T	G	*N/A*	0.0285583	0.0253253	2.59E-01
rs7969592	G	A	*N/A*	-0.00749435	0.021201	7.24E-01
rs10758669	A	C	*N/A*	-0.0320772	0.022662	1.57E-01
rs6827756	C	T	*N/A*	0.00032389	0.0283166	9.91E-01
rs4703855	T	C	*N/A*	-0.0272157	0.0238445	2.54E-01
rs9457247	T	C	*N/A*	0.0471592	0.0215914	2.90E-02
rs2395022	C	A	*N/A*	-0.148553	0.0564041	8.45E-03
rs77981966	T	C	*N/A*	0.0810328	0.0633781	2.01E-01
rs11691685	G	A	*N/A*	-0.0302678	0.0377908	4.23E-01
rs13407913[b]	G	A	*N/A*	0.0715351	0.0223435	1.37E-03
rs7608910	G	A	*N/A*	0.0403862	0.0223608	7.09E-02
rs10995271	C	G	*N/A*	0.0154772	0.0228155	4.98E-01
rs559928	C	T	*N/A*	0.101017	0.0285056	3.94E-04
rs7438704	G	A	*N/A*	-0.0261281	0.0217537	2.30E-01
rs79980175	C	A	*N/A*	0.0304887	0.0333427	3.61E-01
rs6111031	T	C	*N/A*	-0.0178831	0.0317653	5.73E-01
rs2847293	T	A	*N/A*	-0.0897159	0.0289221	1.92E-03
rs10800309	G	A	*N/A*	0.0256305	0.0228565	2.62E-01
rs13001325	T	C	*N/A*	-0.00951347	0.0214334	6.57E-01
rs12694846	G	A	*N/A*	-0.0245752	0.0260148	3.45E-01
rs9491892	G	T	*N/A*	0.116603	0.029044	5.95E-05
rs11185982	C	T	*N/A*	0.0424091	0.0309218	1.70E-01
rs34787213	T	C	*N/A*	-0.0413983	0.032111	1.97E-01
rs12949918	C	T	*N/A*	0.00267491	0.0206428	8.97E-01

rs3853824	C	T	*N/A*	0.0227164	0.0231198	3.26E-01
rs17293632	T	C	*N/A*	0.05234	0.0254438	3.97E-02
rs17391694[b]	T	C	*N/A*	0.0363267	0.0365178	3.20E-01
rs6740462	A	C	*N/A*	0.00814001	0.0259303	7.54E-01
rs3024505	A	G	*N/A*	0.133113	0.028255	2.46E-06
rs2641348	G	A	*N/A*	0.0601945	0.032431	6.34E-02
rs7517847	G	T	*N/A*	-0.0317047	0.0214929	1.40E-01
rs17129991	T	C	*N/A*	0.0045851	0.122949	9.70E-01
rs3197999[b]	A	G	*N/A*	0.0210369	0.024032	3.81E-01
rs76906269	G	A	*N/A*	0.0770825	0.0878741	3.80E-01
rs11159833	T	C	*N/A*	0.0238688	0.0359346	5.07E-01
rs7786444	T	C	*N/A*	-0.0322023	0.0359993	3.71E-01
rs10956252	G	C	*N/A*	0.0109544	0.02234	6.24E-01
rs6651252	C	T	*N/A*	-0.0293114	0.031747	3.56E-01
rs1292053	G	A	*N/A*	0.0726748	0.0216758	8.00E-04
rs7848647	C	T	*N/A*	0.106979	0.0230036	3.31E-06
rs3129871	C	A	*N/A*	0.0734955	0.0224519	1.06E-03
rs6074022	T	C	*N/A*	-0.0957914	0.0240747	6.92E-05
rs12411259	A	G	*N/A*	-0.0533721	0.0249164	3.22E-02
rs6738490	C	T	*N/A*	0.0451056	0.0224739	4.47E-02
rs34804116	A	C	*N/A*	0.0118212	0.0334159	7.24E-01
rs11167518	A	C	*N/A*	0.00496128	0.0391067	8.99E-01
rs7711427	C	A	*N/A*	0.00279048	0.0228632	9.03E-01
rs1363907	A	G	*N/A*	0.0752304	0.0218581	5.78E-04
rs1517352	C	A	*N/A*	-0.0953422	0.0218891	1.33E-05
rs10878302	A	T	*N/A*	0.00487217	0.0443393	9.13E-01
rs6561151	A	G	*N/A*	-0.00684871	0.024383	7.79E-01
rs8127691	C	T	*N/A*	-0.0700581	0.021273	9.90E-04
rs2413583	T	C	*N/A*	-0.0194816	0.0288081	4.99E-01
rs9494844	A	C	*N/A*	-0.0402763	0.0244763	9.99E-02
rs6679677	A	C	*N/A*	0.112829	0.0356831	1.57E-03
rs11713774	C	T	*N/A*	0.0510151	0.0323906	1.15E-01
rs516246[b]	T	C	*N/A*	0.0305411	0.021373	1.53E-01
rs6908425	C	T	*N/A*	0.0170635	0.0268218	5.25E-01
rs1847472	A	C	*N/A*	0.0180198	0.0223493	4.20E-01
rs9889296	A	G	*N/A*	-0.0446737	0.0251467	7.56E-02
rs10798069	G	T	*N/A*	-0.0294987	0.0212483	1.65E-01
rs6738394	G	A	*N/A*	-0.0499887	0.0215112	2.01E-02
rs2538470	G	A	*N/A*	-0.0486628	0.0231468	3.55E-02
rs34779708	G	T	*N/A*	0.0494725	0.0212999	2.02E-02
rs7085798	A	C	*N/A*	-0.0222026	0.0213856	2.99E-01

rs9554587	G	A	*N/A*	-0.119893	0.027731	1.54E-05
rs26528[b]	C	T	*N/A*	-0.00439697	0.0233062	8.50E-01
rs1297258	T	C	*N/A*	0.0520673	0.0215502	1.57E-02
rs140143	G	T	*N/A*	-0.084875	0.0222213	1.34E-04
rs727563	C	T	*N/A*	0.096481	0.028704	7.76E-04

b SNPs associated with potential confounders. SNP: single nucleotide polymorphism; A1: effect allele; A2: baseline allele; EAF: effect allele frequency; SE: standard error. All statistical tests were two-sided. *N/A*, Not available. SNPs rs212388 was not available in the outcome GWAS, Thus, rs212388 was removed in the MR analysis. A *P*-value<5×10^{-8} (rs3184504) was considered genome-wide significant and should be excluded to meet the assumption that requires instruments to be associated with the outcome only through exposure.

Supplementary Table 13. Summary information on PBC for the 53 genome-wide significant SNPs associated with UC

SNP	A1	A2	EAF	Bata	SE	*P*-value
rs1990760[b]	T	C	*N/A*	-0.0799991	0.0219391	0.000265913
rs10460566	A	G	*N/A*	-0.101145	0.0251562	5.80323E-05
rs111830527	A	G	*N/A*	-0.0265853	0.0505491	0.598937
rs7547569[b]	C	T	*N/A*	0.0861795	0.0457405	0.0595525
rs3776414	G	T	*N/A*	0.0426516	0.0222792	0.0555674
rs4947328	G	A	*N/A*	-0.0792811	0.0762438	0.298416
rs59418206	A	G	*N/A*	0.0438477	0.0199354	0.0278433
rs12720356[b]	C	A	*N/A*	-0.192677	0.0381564	4.42568E-07
rs76904798	T	C	*N/A*	0.00514155	0.0413699	0.901092
rs4456788	A	G	*N/A*	-0.0692619	0.021334	0.00116813
rs2497318	T	C	*N/A*	0.0229023	0.0222374	0.303057
rs10758669	A	C	*N/A*	-0.0320772	0.022662	0.156932
rs13136827	C	T	*N/A*	-0.133187	0.0466271	0.0042843
rs34659678	T	C	*N/A*	0.0194809	0.0568552	0.731868
rs2395022	C	A	*N/A*	-0.148553	0.0564041	0.0084454
rs7608910	G	A	*N/A*	0.0403862	0.0223608	0.070899
rs6111031	T	C	*N/A*	-0.0178831	0.0317653	0.573451
rs9611131	C	T	*N/A*	-0.0185007	0.031948	0.56253
rs483905	A	G	*N/A*	-0.0383232	0.0235492	0.103659
rs941823	C	T	*N/A*	-0.0340812	0.0252548	0.177178
rs10185424	G	T	*N/A*	0.017634	0.0212974	0.407677
rs3024493	A	C	*N/A*	0.133212	0.0283991	2.72258E-06
rs1182188[b]	C	T	*N/A*	0.0176448	0.0238702	0.459787
rs12318183	A	C	*N/A*	-0.066656	0.0228903	0.00359153
rs12718244	A	G	*N/A*	-0.016444	0.0215983	0.446444
rs17780256	C	A	*N/A*	-0.0117797	0.0280071	0.67405
rs11676348	T	C	*N/A*	0.0298525	0.0216003	0.166959
rs6426833	A	G	*N/A*	-0.0404559	0.0208602	0.0524542
rs76546301	A	G	*N/A*	0.0192295	0.0815106	0.813499
rs7404095	C	T	*N/A*	0.0151919	0.0249848	0.543158
rs9836291[b]	A	G	*N/A*	0.0220202	0.0237831	0.35451
rs61893460	A	G	*N/A*	0.0550768	0.0233769	0.018471
rs2836883	A	G	*N/A*	0.0159197	0.026896	0.553919
rs11230563	T	C	*N/A*	-0.0349185	0.0216253	0.106375
rs9891119	C	A	*N/A*	0.0324795	0.0231119	0.159928
rs10748783	A	C	*N/A*	-0.00261018	0.0208227	0.900245
rs272882	T	G	*N/A*	0.0109851	0.0271274	0.685518
rs7711427	C	A	*N/A*	0.00279048	0.0228632	0.902858

rs9941524	G	A	*N/A*	-0.0337091	0.0213249	0.113938
rs1517352	C	A	*N/A*	-0.0953422	0.0218891	1.32642E-05
rs4366152	C	T	*N/A*	0.1036	0.0231023	7.31206E-06
rs4712520	C	T	*N/A*	0.0103907	0.0286806	0.717135
rs4656958	G	A	*N/A*	0.0288838	0.0235446	0.21991
rs7738430	C	T	*N/A*	0.0851593	0.0688798	0.21633
rs55808324	A	G	*N/A*	0.0235854	0.0353592	0.504758
rs10910092	G	A	*N/A*	0.112722	0.0221405	3.55762E-07
rs35223180	T	G	*N/A*	-0.0305449	0.0283631	0.281514
rs13430791	A	G	*N/A*	0.0572797	0.0312652	0.0669422
rs4676410	A	G	*N/A*	-0.0123809	0.0273875	0.651222
rs6466198	T	A	*N/A*	0.0227941	0.0228554	0.31861
rs2274351	C	T	*N/A*	-0.0383923	0.0219316	0.080024
rs1297256	T	C	*N/A*	0.0551142	0.0216826	0.0110266
rs140143	G	T	*N/A*	-0.084875	0.0222213	0.000133703

b SNPs associated with potential confounders. SNP: single nucleotide polymorphism; A1: effect allele; A2: baseline allele; EAF: effect allele frequency; SE: standard error. All statistical tests were two-sided. *N/A*, Not available. A *P*-value$<5\times10^{-8}$ was considered genome-wide significant and should be excluded to meet the assumption that requires instruments to be associated with the outcome only through exposure.

Supplementary Table 14. Summary information on PBC SNPs used as genetic instruments for the Mendelian randomization analyses

SNP	A1	A2	EAF	Bata	SE	N	*P*-value	R^2	F
**47 SNPs for PBC**									
rs6679356	T	C	0.828	-0.43936	0.026143	24510	6.6146E-63	0.011392	282.4134719
rs10802191	A	T	0.1213	-0.20804	0.037467	24510	2.8146E-08	0.001256	30.82883815
rs945635	G	C	0.4742	-0.1222	0.021623	24510	1.5924E-08	0.001301	31.93439512
rs12123169	A	T	0.1849	0.217895	0.025404	24510	9.7521E-18	0.002993	73.56107097
rs3913893	G	C	0.3181	-0.13754	0.023477	24510	4.6794E-09	0.001398	34.31555483
rs867436	T	C	0.334	0.134167	0.022616	24510	2.9857E-09	0.001434	35.19041491
rs859767	G	A	0.4364	-0.13931	0.023065	24510	1.5432E-09	0.001486	36.4759083
rs34655300	T	C	0.4493	0.136669	0.022001	24510	5.2323E-10	0.001572	38.58546157
rs3771317	C	T	0.1441	0.289503	0.029772	24510	2.4022E-22	0.003843	94.54914246
rs2197017	G	C	0.3588	0.139128	0.022662	24510	8.2869E-10	0.001535	37.68848436
rs589446	T	G	0.3499	-0.35309	0.02244	24510	1.9638E-55	0.010001	247.5782871
rs6550965	A	C	0.4205	0.163094	0.021537	24510	3.6526E-14	0.002334	57.34430296
rs2293370	A	G	0.164	-0.29901	0.028939	24510	5.5399E-25	0.004337	106.747334
rs7674640	T	C	0.492	0.216421	0.02209	24510	1.5635E-22	0.003901	95.97978917
rs35467801	GT	G	0.2724	-0.22366	0.025155	24510	6.1532E-19	0.003215	79.04783257
rs2546890	G	A	0.5089	-0.14423	0.021614	24510	2.505E-11	0.001814	44.52618665
rs35127065	T	C	0.172	0.159067	0.028928	24510	3.8237E-08	0.001232	30.23451015
rs3131789	G	A	0.5775	0.180174	0.021912	24510	2.0017E-16	0.002751	67.60407434
rs7774434	C	T	0.4294	0.470454	0.021656	24510	3.681E-104	0.018892	471.9092056
rs928976	T	C	0.329	0.410549	0.022469	24510	1.5035E-74	0.013438	333.8313119
rs2327832	G	A	0.168	0.161159	0.025422	24510	2.3059E-10	0.001637	40.18536583
rs7805218	A	G	0.3539	0.128538	0.023431	24510	4.1173E-08	0.001226	30.09087854
rs12531711	G	A	0.1014	0.4202	0.0313452	24510	8.57433E-41	0.007279	179.6944096
rs60600003	G	T	0.0974	0.253264	0.035012	24510	4.7033E-13	0.00213	52.32119376
rs11390003	GA	G	0.2177	-0.14917	0.026786	24510	2.5607E-08	0.001264	31.01224345
rs7097397	A	G	0.3956	-0.14377	0.022706	24510	2.4196E-10	0.001633	40.091384
rs4936443	T	C	0.8101	0.367095	0.029743	24510	5.3926E-35	0.006177	152.3162069
rs7130339	A	G	0.5159	0.121645	0.022264	24510	4.6632E-08	0.001216	29.84962618
rs11601860	T	A	0.3668	-0.146	0.022776	24510	1.451E-10	0.001674	41.09044442
rs12419634	C	G	0.6213	0.128326	0.022268	24510	8.2769E-09	0.001353	33.20624347
rs35350651	AC	A	0.5229	-0.19125	0.021481	24510	5.5005E-19	0.003224	79.26623982
rs1800693	C	T	0.4254	0.179759	0.021699	24510	1.1907E-16	0.002792	68.62247974
rs9533122	G	A	0.5368	0.155125	0.021532	24510	5.8304E-13	0.002113	51.89904987
rs9591325	C	T	0.0547	-0.45188	0.050169	24510	2.1429E-19	0.003299	81.12367196
rs72699866	A	G	0.1859	-0.19516	0.029338	24510	2.8894E-11	0.001802	44.24683295
rs3784099	A	G	0.3439	-0.20302	0.02437	24510	8.3062E-17	0.002824	69.39444839
rs59643720	C	A	0.2356	0.316412	0.024452	24510	2.7328E-38	0.006785	167.4277609
rs1119132	G	A	0.8887	0.20264	0.032812	24510	6.581E-10	0.001554	38.1379592
rs9652601	A	G	0.3340	-0.23995	0.023792	24510	6.6927E-24	0.004133	101.7096821
rs4780355	C	T	0.3340	-0.19955	0.024019	24510	1.0083E-16	0.002808	69.02032473
rs79577483	G	A	0.1412	0.211743	0.031246	24510	1.23E-11	0.00187	45.91914863
rs11117432	A	G	0.2187	-0.27323	0.026862	24510	2.8158E-24	0.004203	103.4518537
rs8067378	G	A	0.5149	0.259633	0.021526	24510	1.7519E-33	0.0059	145.4674218
rs1808094	C	T	0.5249	-0.12774	0.021492	24510	2.7892E-09	0.001439	35.32269889

rs3745516	G	A	0.7704	-0.27432	0.023975	24510	2.6467E-30	0.005313	130.9091417
rs2304256	A	C	0.2624	-0.20525	0.02443	24510	4.4289E-17	0.002872	70.57982477
rs137687	A	G	0.4374	-0.21776	0.021872	24510	2.3741E-23	0.004028	99.11510903

SNP: single nucleotide polymorphism; A1: effect allele; A2: baseline allele; EAF: effect allele frequency; SE: standard error. N refers to the sample size of the initial GWAS from which the genetic variants were selected. All statistical tests were two-sided. A *P*-value<5×10^{-8} was considered genome-wide significant.

Supplementary Table 15. Potential confounders of PBC SNPs under the condition of *P*<5×10^{-8} in the PhenoScanner database

Exposure	Excluded SNP	Trait
**PBC**	rs60600003[c]	celiac disease
**PBC**	rs2327832[c]	celiac disease

c was removed in the MR analysis for Sarcoidosis during heterogeneity test via RadialMR. SNP: single nucleotide polymorphism. All statistical tests were two-sided. A *P*-value<5×10^{-8} with a potential confounder in the PhenoScanner database was considered genome-wide significant and removed.

Supplementary Table 16. Summary information on Sarcoidosis for the 44 genome-wide significant SNPs associated with PBC

SNP	A1	A2	EAF	Bata	SE	*P*-value
rs6679356	C	T	0.901397	-0.0969026	0.0333102	0.00362468
rs10802191	T	A	0.152966	-0.0485069	0.0285334	0.0891292
rs945635	C	G	0.416726	-0.00776254	0.0207503	0.708335
rs12123169	T	A	0.145679	0.0969017	0.028272	0.000609228
rs3913893	C	G	0.242199	0.0353948	0.0238747	0.1382
rs34655300	C	T	0.431088	0.0477764	0.0207531	0.0213275
rs859767	A	G	0.334226	-0.0178357	0.0218745	0.414862
rs3771317	T	C	0.113054	0.0100585	0.0324672	0.756709
rs2197017	G	C	0.573742	-0.0420312	0.0207447	0.0427524
rs6550965	C	A	0.512309	-0.00214122	0.0205566	0.917041
rs2293370	G	A	0.127226	-0.0135382	0.0305582	0.657744
rs589446	G	T	0.377643	0.0133589	0.0211302	0.527244
rs7674640	C	T	0.458156	0.055037	0.0204795	0.00720079
rs2546890	A	G	0.409501	0.00352487	0.0208163	0.865535
rs35127065	C	T	0.173263	-0.0325466	0.0271252	0.23019
rs3131789	A	G	0.55882	-0.0609631	0.0205268	0.00297872
rs2327832[c]	A	G	0.189093	0.0600261	0.0260366	0.0211412
rs60600003[c]	T	G	0.101995	0.0802967	0.0337953	0.0175029
rs12531711	A	G	0.15023	0.0662895	0.0285001	0.0200221
rs7097397	G	A	0.371723	0.00843566	0.0213132	0.692256
rs12419634	G	C	0.536602	0.023893	0.0205874	0.245818
rs4936443	C	T	0.833799	0.0215759	0.027386	0.430787
rs1800693	T	C	0.415942	0.0604078	0.0206717	0.00347512
rs9533122	A	G	0.613033	0.0122521	0.0210727	0.560959
rs9591325	T	C	0.0613089	-0.142914	0.0442567	0.00124134
rs3784099	G	A	0.305215	0.00618007	0.0223511	0.782164
rs72699866	G	A	0.139637	0.0262492	0.0296813	0.376497
rs59643720	A	C	0.231617	0.00907468	0.024199	0.707659
rs9652601	G	A	0.302734	-0.0438225	0.0224168	0.0505953
rs4780355	T	C	0.301511	-0.094111	0.0226196	3.17424E-05
rs1119132	A	G	0.907113	0.0693929	0.0353048	0.0493526
rs79577483	A	G	0.159942	-0.124497	0.0286273	1.36823E-05
rs11117432	G	A	0.280491	-0.0201814	0.0229026	0.378218
rs8067378	A	G	0.56008	-0.061633	0.0205447	0.00270035
rs2304256	C	A	0.253806	-0.0600389	0.0235644	0.0108385
rs3745516	A	G	0.817038	-0.00896605	0.0263899	0.734042
rs137687	G	A	0.390405	-0.00269387	0.0209816	0.897839

c SNPs associated with potential confounders. SNP: single nucleotide polymorphism; A1: effect allele; A2: baseline allele; EAF: effect allele frequency; SE: standard error. All statistical tests were two-sided. A *P*-value$<5\times10^{-8}$ was considered genome-wide significant and should be excluded to meet the assumption that requires instruments to be associated with the outcome only through exposure.

Supplementary Table 17. Summary information of removed pleiotropic SNPs associated with IBD, CD and UC and Sarcoidosis

Exposure traits	Outcome traits	PhenoScanner database	Incompatible SNPs	Palindromic SNPs	MR-PRESSO outlier test
**IBD**	**Sarcoidosis**	rs13407913 rs9836291 rs9273363 rs1182188 rs3184504 rs62037363 rs1267499 rs2488397 rs7657746 rs9264942 rs6933404 rs780094	rs11768997 rs140143	*N/A*	rs367569 rs6588248 rs67643815 rs7547569 rs7608910 rs769177
**CD**		rs17391694 rs26528 rs3197999 rs3184504 rs727563 rs1267501 rs6702421 rs9264942 rs3129871 rs212388 rs780094 rs13407913	rs11768997 rs140143	rs1927681	rs1646019 rs7608910
**UC**		rs9836291 rs16841904 rs13136827 rs9271858 rs2516440 rs9271255 rs6920220 rs1182188 rs7547569 rs1990760 rs1517352 rs4947328 rs10748783 rs12132349 rs12720356	rs140143	rs10870077 rs1927681	rs7608910 rs56167332 rs7738430

SNP: single nucleotide polymorphism; MR-PRESSO, MR pleiotropy residual sum and outlier; IBD, Inflammatory bowel disease; UC, Ulcerative colitis; CD, Crohn's disease.

Supplementary Table 18. Summary information of removed pleiotropic SNPs associated with Sarcoidosis and IBD, CD and UC

Exposure traits	Outcome traits	PhenoScanner database	Incompatible SNPs	Palindromic SNPs	MR-PRESSO outlier test
**Sarcoidosis**	**IBD**	*N/A*	*N/A*	rs4766578	rs11645302 rs1431403 rs35202091 rs3760112
	**CD**	*N/A*	*N/A*	rs4766578	rs35202091 rs11645302 rs12663194
	**UC**	*N/A*	*N/A*	rs4766578	rs11645302 rs34536443

SNP: single nucleotide polymorphism; MR-PRESSO, MR pleiotropy residual sum and outlier; IBD, Inflammatory bowel disease; UC, Ulcerative colitis; CD, Crohn's disease.

Supplementary Table 19. Summary information of removed pleiotropic SNPs associated with IBD, CD and UC and PBC

Exposure traits	Outcome traits	PhenoScanner database	Incompatible SNPs	Palindromic SNPs	MR-PRESSO outlier test
**IBD**	**PBC**	rs3184504 rs780094<br>rs9836291 rs13407913<br>rs62037363 rs516246	rs11768997	rs10878302<br>rs10956252<br>rs6466198<br>rs78487399	rs12318183 rs140143<br>rs1517352 rs181826<br>rs17651741 rs7848647<br>rs6074022 rs7547569<br>rs9557207
**CD**		rs780094 rs26528<br>rs516246 rs3197999<br>rs3184504 rs17391694<br>rs212388 rs13407913	rs11768997	rs10878302<br>rs10956252<br>rs10995271<br>rs2847293	rs140143 rs1517352<br>rs181826 rs9554587<br>rs3024505 rs6074022<br>rs438475 rs61839660<br>rs6679677 rs7848647
**UC**		rs9836291 rs1182188<br>rs7547569 rs1990760<br>rs12720356	*N/A*	rs6466198	rs10460566 rs140143<br>rs10910092 rs1990760<br>rs12318183 rs1517352<br>rs3024493 rs4366152

SNP: single nucleotide polymorphism; MR-PRESSO, MR pleiotropy residual sum and outlier; IBD, Inflammatory bowel disease; UC, Ulcerative colitis; CD, Crohn's disease; PBC, Primary biliary cholangitis.

Supplementary Table 20. Heterogeneity and pleiotropy analysis of Sarcoidosis with IBD, CD and UC using different analytical methods

Exposure traits	Outcome traits	MR methods	Cochran's Q statistic	Heterogeneity *P*-value	Pleiotropy *P*-value	MR-PRESSO global outlier test	
						RSSOBs	*P*-value
**Sarcoidosis**	**IBD**	Inverse variance weighted	3.800	0.051	*N/A*	54.575	0.007
	**CD**	Inverse variance weighted	0.166	0.683	*N/A*	53.678	<0.001
	**UC**	Inverse variance weighted	8.256	0.083	0.591	29.085	0.007

SNP: single nucleotide polymorphism; MR-PRESSO, MR pleiotropy residual sum and outlier; IBD, Inflammatory bowel disease; UC, Ulcerative colitis; CD, Crohn's disease.

Supplementary Table 21. Summary information of removed pleiotropic SNPs associated with PBC and Sarcoidosis

Exposure traits	Outcome traits	PhenoScanner database	Incompatible SNPs	Palindromic SNPs	MR-PRESSO outlier test
**PBC**	**Sarcoidosis**	rs60600003<br>rs2327832	*N/A*	rs10802191 rs945635<br>rs12123169 rs12419634<br>rs2197017 rs3913893	rs3131789<br>rs4780355<br>rs8067378<br>rs79577483

SNP: single nucleotide polymorphism; MR-PRESSO, MR pleiotropy residual sum and outlier; PBC, Primary biliary cholangitis.
