# Supplementary material for: Causal association of inflammatory bowel disease with sarcoidosis and the mediating role of primary biliary cholangitis
Source: Front Immunol. 2024 Sep 3;15:1448724. doi: 10.3389/fimmu.2024.1448724 (PMC11406174; doi:10.3389/fimmu.2024.1448724)
Supplement: Supplementary file 2 [file DataSheet2.pdf]

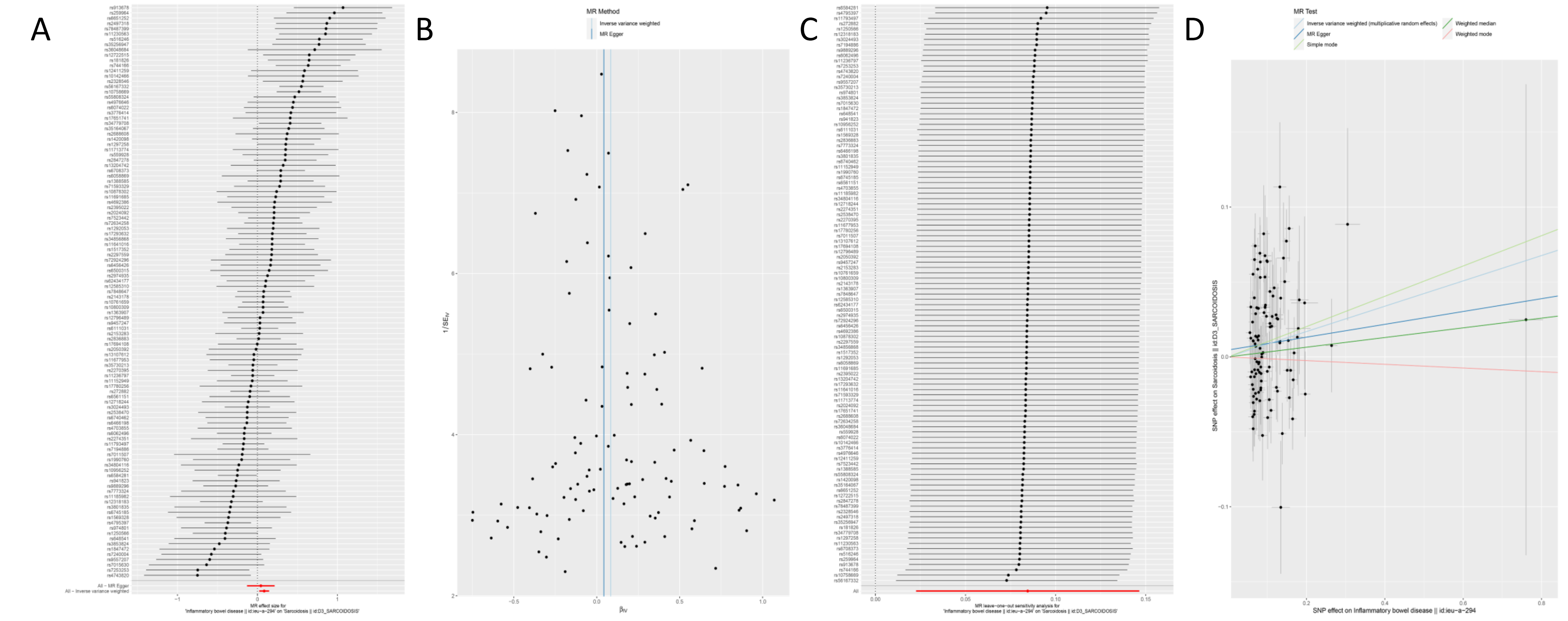

Supplementary Figure 1 **A** Forest plot for the causal effect of each SNP on sarcoidosis risk. **B** Funnel plot for the overall heterogeneity in the effect of IBD on sarcoidosis risk. **C** Leave-one-out analysis suggested that no single instrument was strongly driving the overall effect of IBD on sarcoidosis. **D** Scatter plot for the causal effect of IBD on sarcoidosis risk. IBD, Inflammatory bowel disease.

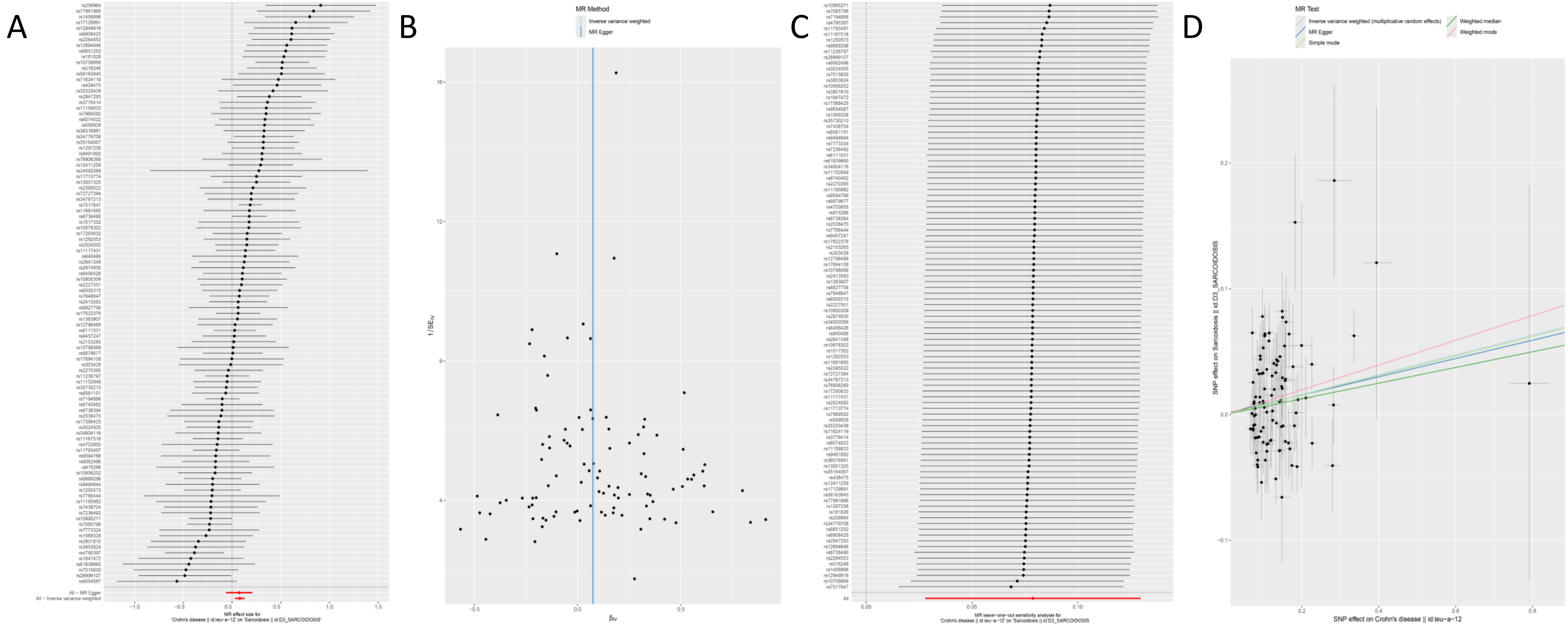

Supplementary Figure 2 **A** Forest plot for the causal effect of each SNP on sarcoidosis risk. **B** Funnel plot for the overall heterogeneity in the effect of CD on sarcoidosis risk. **C** Leave-one-out analysis suggested that no single instrument was strongly driving the overall effect of CD on sarcoidosis. **D** Scatter plot for the causal effect of CD on sarcoidosis risk. CD, Crohn's disease.

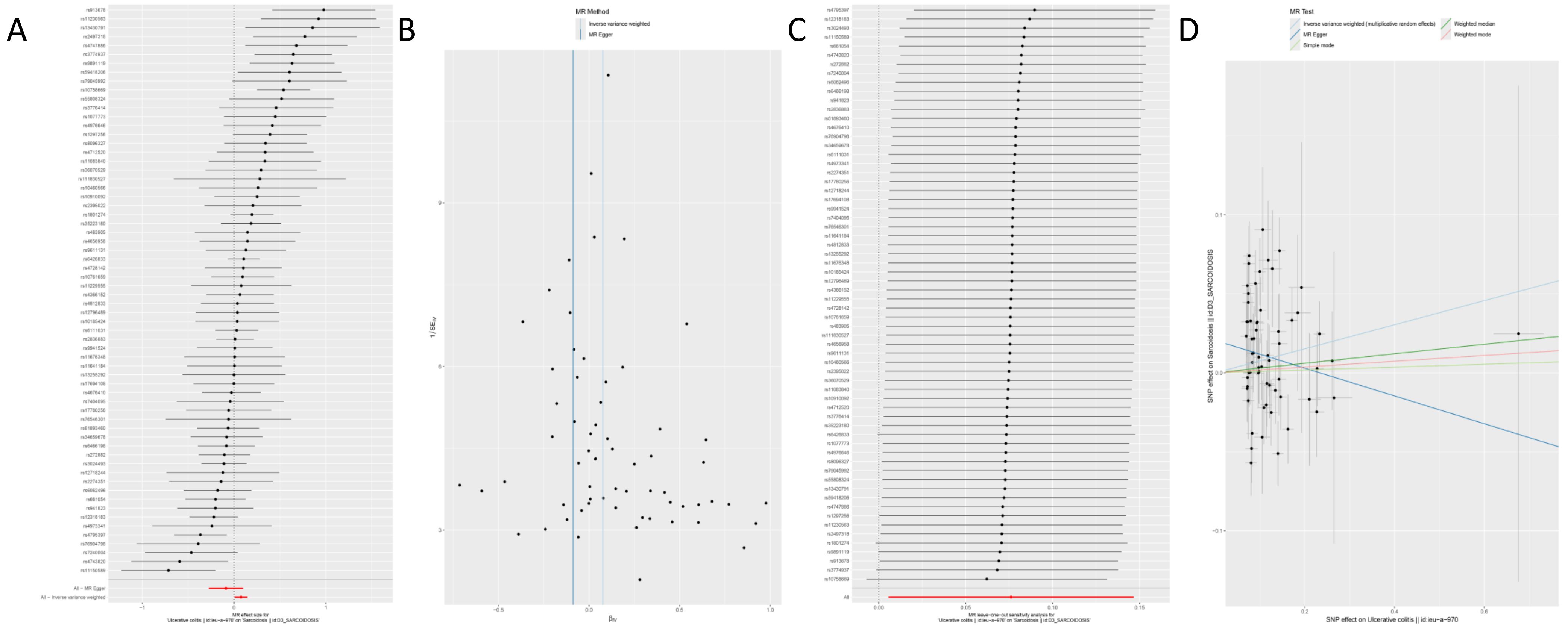

Supplementary Figure 3 **A** Forest plot for the causal effect of each SNP on sarcoidosis risk. **B** Funnel plot for the overall heterogeneity in the effect of UC on sarcoidosis risk. **C** Leave-one-out analysis suggested that no single instrument was strongly driving the overall effect of UC on sarcoidosis. **D** Scatter plot for the causal effect of UC on sarcoidosis risk. UC, Ulcerative colitis.

Exposure	Outcome	Methods	OR	OR95CI	pval
sarcoidosis	IBD	Inverse variance weighted	1.013	(0.796 , 1.289)	0.919
sarcoidosis	CD	Inverse variance weighted	1.306	(1.110 , 1.537)	1.290×10^{-3}
sarcoidosis	UC	Inverse variance weighted	1.104	(0.970 , 1.257)	0.132
		MR egger	1.365	(0.673 , 2.769)	0.452
		Weighted median	1.078	(0.961 , 1.210)	0.201
		Simple mode	1.076	(0.937 , 1.236)	0.359
		Weighted mode	1.086	(0.950 , 1.241)	0.294

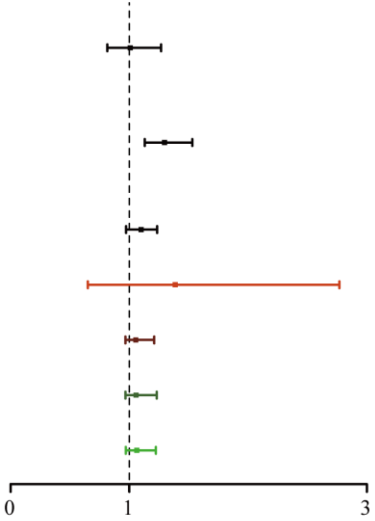

Supplementary Figure 4 Causal estimates given as odds ratios (OR) and 95%confidence intervals for the effect of sarcoidosis on IBD, CD and UC. IBD, Inflammatory bowel disease; CD, Crohn’s disease; UC, Ulcerative colitis.

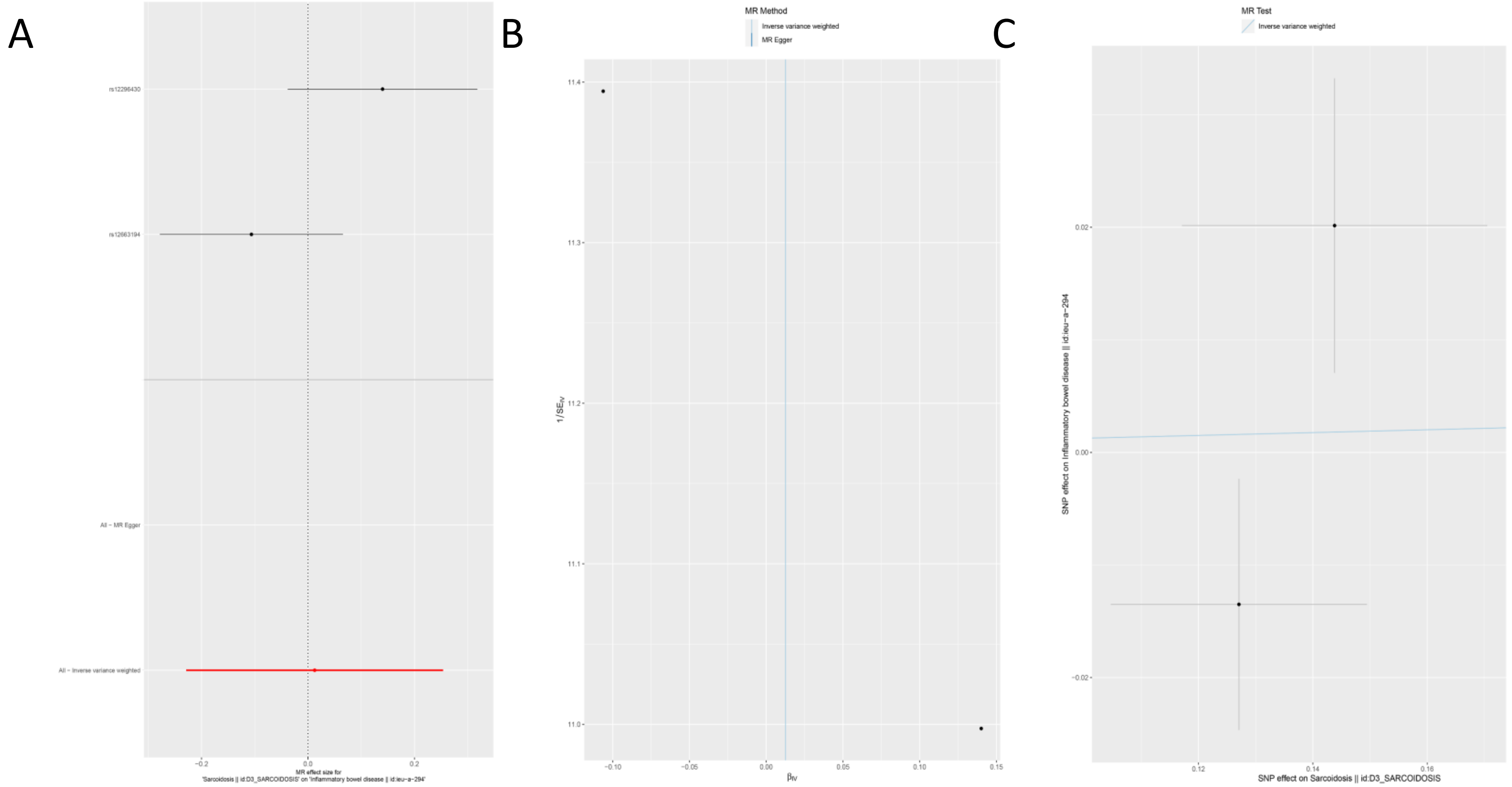

Supplementary Figure 5 **A** Forest plot for the causal effect of each SNP on IBD risk. **B** Funnel plot for the overall heterogeneity in the effect of sarcoidosis on IBD risk. **C** Scatter plot for the causal effect of sarcoidosis on IBD risk. IBD, Inflammatory bowel disease.

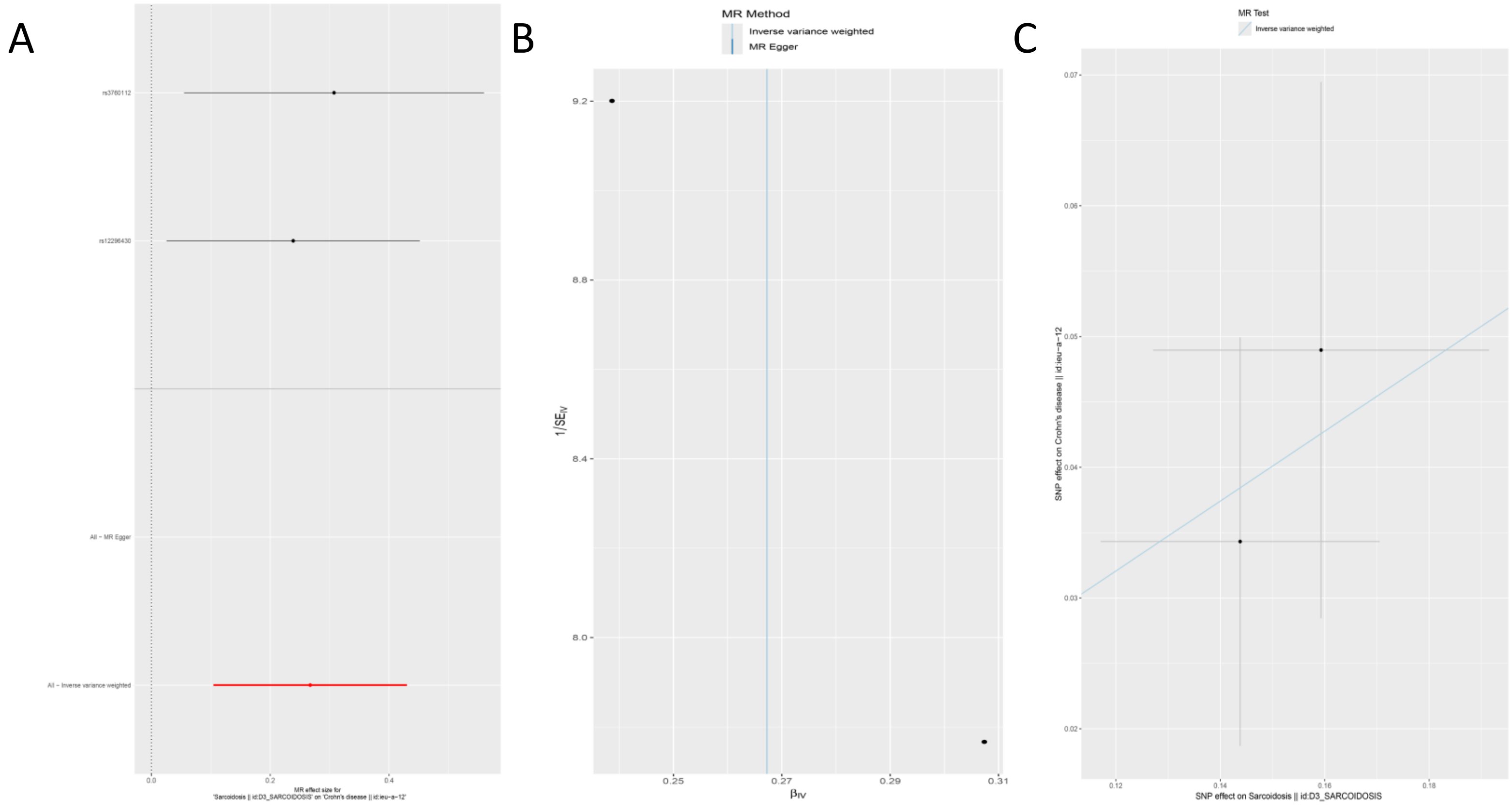

Supplementary Figure 6 **A** Forest plot for the causal effect of each SNP on sarcoidosis risk. **B** Funnel plot for the overall heterogeneity in the effect of sarcoidosis on CD risk. **C** Scatter plot for the causal effect of sarcoidosis on CD risk. CD, Crohn's disease.

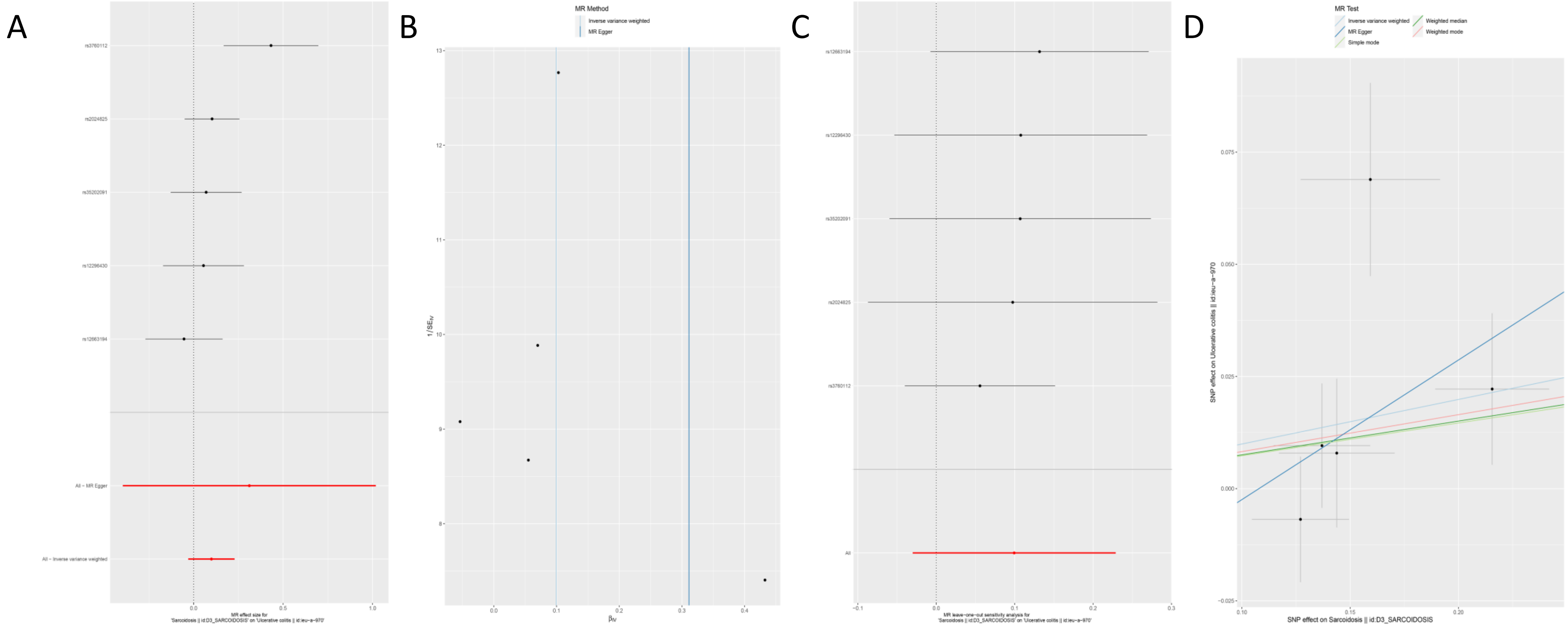

Supplementary Figure 7 **A** Forest plot for the causal effect of each SNP on sarcoidosis risk. **B** Funnel plot for the overall heterogeneity in the effect of sarcoidosis on UC risk. **C** Scatter plot for the causal effect of sarcoidosis on UC risk. UC, Ulcerative colitis.

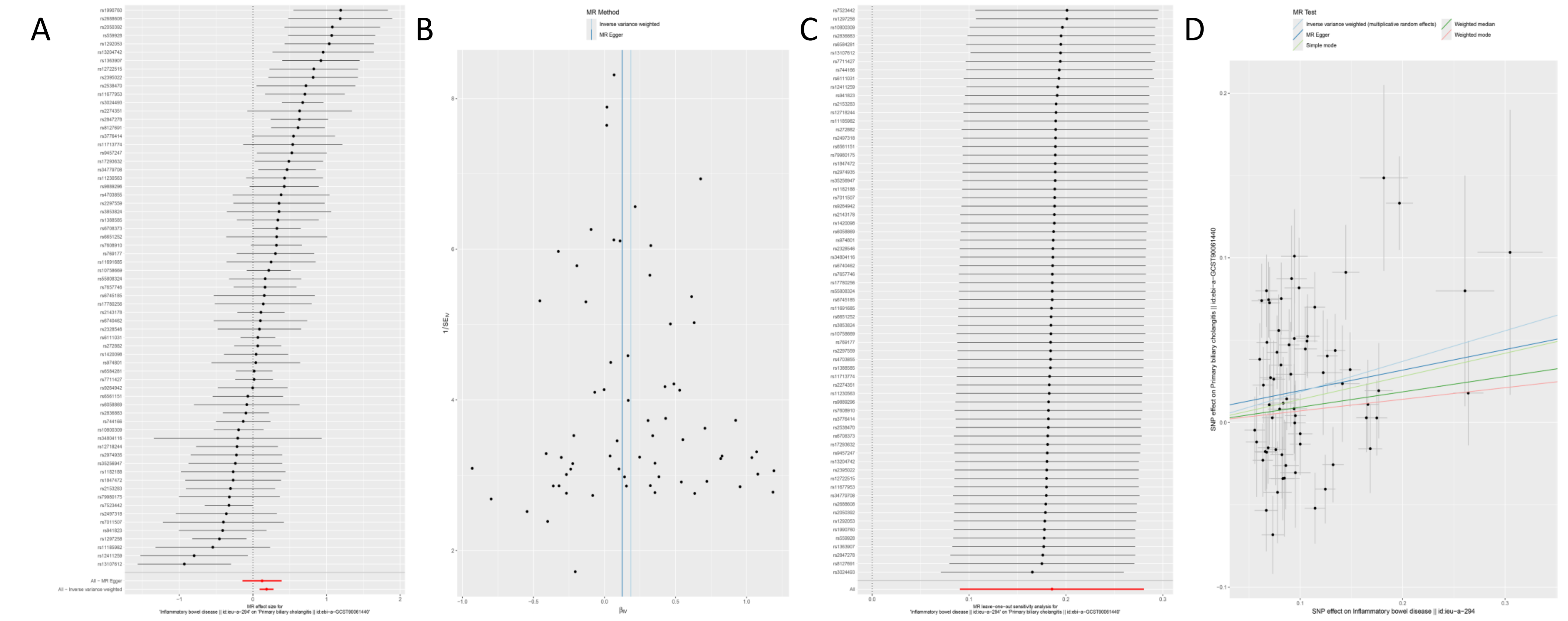

Supplementary Figure 8 **A** Forest plot for the causal effect of each SNP on PBC risk. **B** Funnel plot for the overall heterogeneity in the effect of IBD on PBC risk. **C** The leave-one-SNP out analysis was conducted to assess the influence of individual variants of IBD on PBC. **D** Scatter plot for the causal effect of IBD on PBC risk. IBD, Inflammatory bowel disease; PBC, Primary biliary cholangitis.

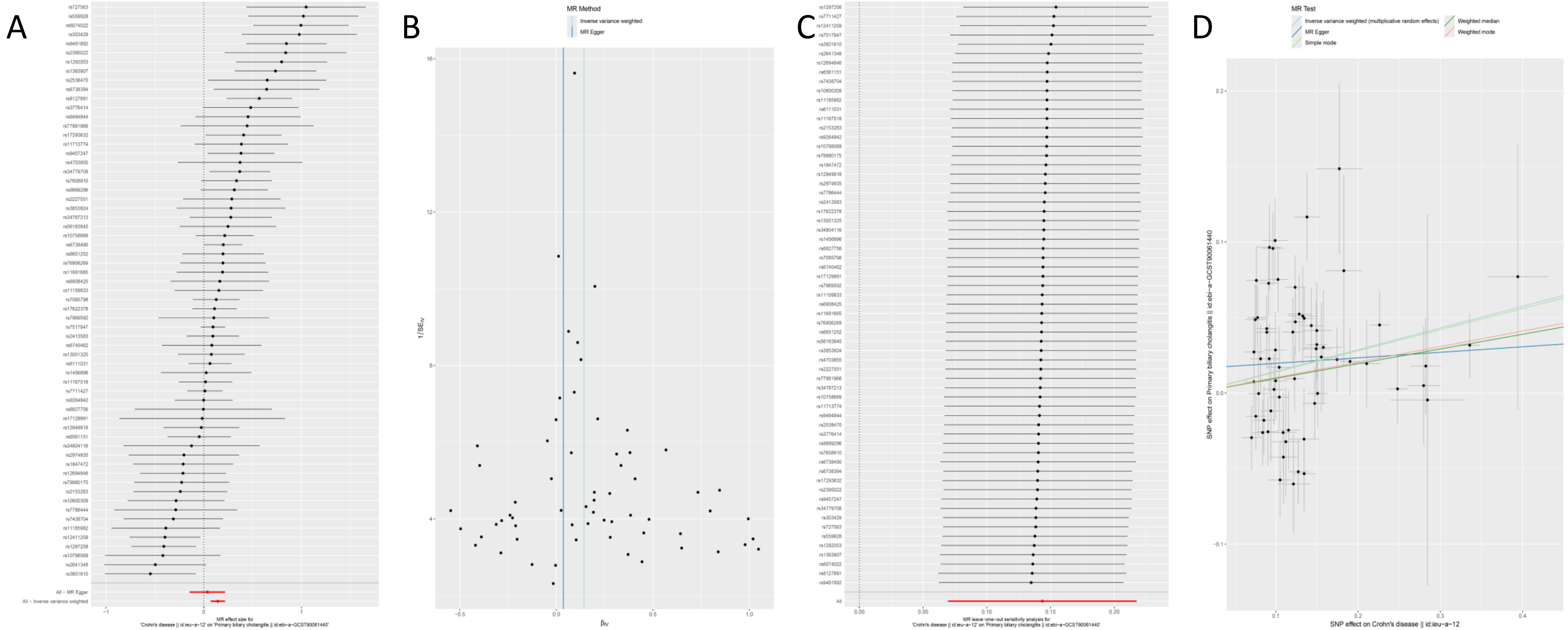

Supplementary Figure 9 **A** Forest plot for the causal effect of each SNP on PBC risk. **B** Funnel plot for the overall heterogeneity in the effect of CD on PBC risk. **C** The leave-one-SNP out analysis was conducted to assess the influence of individual variants of CD on PBC. **D** Scatter plot for the causal effect of CD on PBC risk. CD, Crohn's disease; PBC, Primary biliary cholangitis.

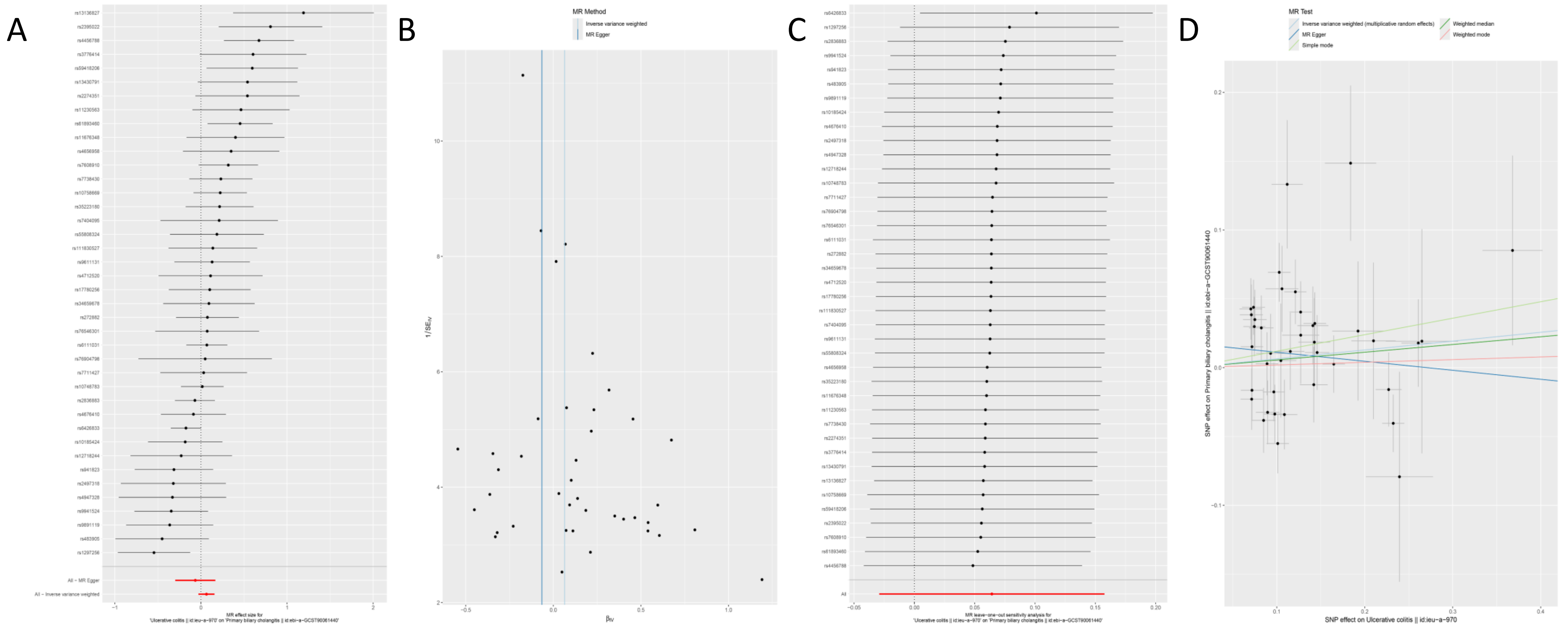

Supplementary Figure 10 **A** Forest plot for the causal effect of each SNP on PBC risk. **B** Funnel plot for the overall heterogeneity in the effect of UC on PBC risk. **C** The leave-one-SNP out analysis was conducted to assess the influence of individual variants of UC on PBC. **D** Scatter plot for the causal effect of UC on PBC risk. UC, Ulcerative colitis; PBC, Primary biliary cholangitis.

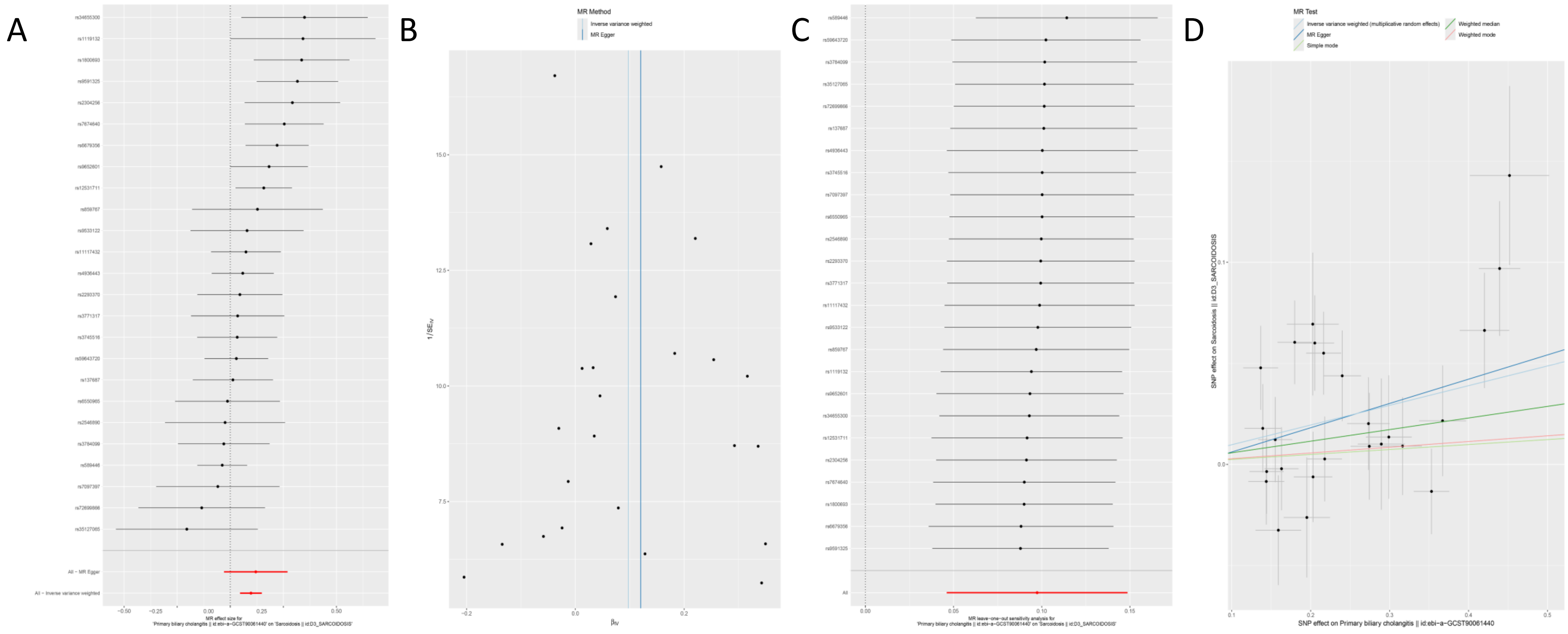

Supplementary Figure 11 **A** Forest plot for the causal effect of each SNP on sarcoidosis risk. **B** Funnel plot for the overall heterogeneity in the effect of PBC on sarcoidosis risk. **C** The leave-one-SNP out analysis was conducted to assess the influence of individual variants of PBC on sarcoidosis. **D** Scatter plot for the causal effect of PBC on sarcoidosis risk. PBC, Primary biliary cholangitis.
