# Supplementary material for: Causal association of inflammatory bowel disease with sarcoidosis and the mediating role of primary biliary cholangitis
Source: Front Immunol. 2024 Sep 3;15:1448724. doi: 10.3389/fimmu.2024.1448724 (PMC11406174; doi:10.3389/fimmu.2024.1448724)
Supplement: Supplementary file 3 [file DataSheet3.pdf]

STROBE-MR checklist of recommended items to address in reports of Mendelian randomization studies[1 2]

Item No.	Section	Checklist item	Page No.	Relevant text from manuscript
1	**TITLE and ABSTRACT**	Indicate Mendelian randomization (MR) as the study's design in the title and/or the abstract if that is a main purpose of the study	Page 1-2	Title:Casual association of inflammatory bowel disease with sarcoidosis and the mediating role of primary biliary cholangitis<br>Abstract:We adopted a Mendelian Randomization (MR) approach to investigate the causal relationship between IBD with genetic susceptibility to sarcoidosis, as well as to explore the potential mediating role of PBC by using a two-step MR approach.
	**INTRODUCTION**			
2	**Background**	Explain the scientific background and rationale for the reported study. What is the exposure? Is a potential causal relationship between exposure and outcome plausible? Justify why MR is a helpful method to address the study question	Page 3-4	Previous observational epidemiological studies have identified a potential association between Inflammatory bowel disease (IBD) and sarcoidosis. Nonetheless, the precise biological mechanisms underlying this association remain unclear. whether the causal effect of IBD on sarcoidosis is mediated by the role of PBC remains unclear. This study aims to investigate the causal association between IBD and sarcoidosis, as well as to explore the potential mediating role of PBC.
3	**Objectives**	State specific objectives clearly, including pre-specified causal hypotheses (if any). State that MR is a method that, under specific assumptions, intends to estimate causal effects	Page 4	Mendelian randomization (MR) is a method frequently employed for exploring causal links between risk factors and outcomes by utilizing single nucleotide polymorphisms (SNPs) as instrumental variables. We employed a two-sample bidirectional MR analysis to assess the potential causal relationship of IBD, including CD and UC, with the risk for sarcoidosis, and indicated that whether PBC could act as a mediator in this connection.
	**METHODS**			
4	**Study design and data sources**	Present key elements of the study design early in the article. Consider including a table listing sources of data for all phases of the study. For each data source contributing to the analysis, describe the following:	Page 7-9	The instrumental variables (IVs), for IBD encompassing CD and UC subtypes, were extracted from a GWAS the International IBD Genetics Consortium (IIBDGC） by Liu et al including 31,665 cases and 33,977 controls of European ancestry, including 13,768 UC and 17,897 CD cases. The genetic association data of sarcoidosis was obtained from a GWAS that consists of 4,854 cases and 446,523 controls (https://r11.finngen.fi/pheno/D3_SARCOIDOSIS) in the European population. And we obtained genetic association data of PBC from a GWAS that consists of 8,021 European cases and 16,489 European controls. The detailed information of the dataset included in the present study is shown in Supplementary Table 1.
	a)	Setting: Describe the study design and the underlying population, if possible. Describe the setting, locations, and relevant dates,	Not mentioned	

			including periods of recruitment, exposure, follow-up, and data collection, when available.		
		b)	Participants: Give the eligibility criteria, and the sources and methods of selection of participants. Report the sample size, and whether any power or sample size calculations were carried out prior to the main analysis	supplematary data:supplematary table 1	supplematary data: supplematary Table 1 reports information such as the sources and sample size.
		c)	Describe measurement, quality control and selection of genetic variants	Page 5-6	Selection of the genetic instrumental variables. Identified SNPs at each significance threshold ($P<5\times10^{-8}$) were clumped for independence using PLINK clumping in the TwoSampleMR tool. Since there were too few SNPs with P-values less than 5×10^{-8} for sarcoidosis, we extended the threshold to 5×10^{-6} to select eligible genetic instrumental variables which had been applied to previous MR research. A strictly cut-off of $R^2<0.001$ and a window of 10,000 kb were used for clumping with the 1000 Genomes European data as the reference panel. The proportions of trait variance explained by the identified SNPs were calculated using the following formulas: $R^2=2\times\beta^2\times MAF\times(1-MAF)/(2\times MAF\times(1-MAF)\times\beta^2+2\times MAF\times(1-MAF)\times N\times se(\beta)^2)$. In addition, we evaluated instrument strength using the F statistic, where $F=(R^2\times(N-k-1))/k(1-R^2)$, to test the significant association of the genetic instruments with the exposure, and SNPs with F <10 were excluded, as an F ≥ 10 indicates a relatively low risk of weak instrument bias in MR analysis.
		d)	For each exposure, outcome, and other relevant variables, describe methods of assessment and diagnostic criteria for diseases	Supplematary data:supplematary tables	Supplematary data:supplematary table 1 to supplematary table 21.
		e)	Provide details of ethics committee approval and participant informed consent, if relevant	Page 12	All GWAS summary data are publicly available and therefore no additional ethical approval or informed consent was required.
5	**Assumptions**		Explicitly state the three core IV assumptions for the main analysis (relevance, independence and exclusion restriction) as well assumptions for any additional or sensitivity analysis	Page 4-7	We used a two-sample MR design: a genetic instrumental variable analysis based on summary-level data with SNPs as instruments for the risk factor. For causal estimates from MR studies to be valid, three assumptions must be adhere to: (1) the genetic variants are highly associated with the exposure, (2) the genetic variants are not associated with any potential confounder of the exposure-outcome association, and (3) the variants exclusively affects the outcome through the exposure.<br>The core assumptions are reflected in the analysis method. The methods of sensitivity analysis and horizontal pleiotropy testing are also described: weighted median, MR-Egger and MR-PRESSO, and the use of F-statistics to assess statistical power and Q-statistics to assess heterogeneity are reported.
6	**Statistical methods: main analysis**		Describe statistical methods and statistics used		

		a) Describe how quantitative variables were handled in the analyses (i.e., scale, units, model)	Page 7	If Cochran's Q test suggested significant heterogeneity (*P*<0.05), we turned from the fixed inverse variance weighted model to the random effects model.
		b) Describe how genetic variants were handled in the analyses and, if applicable, how their weights were selected	Not mentioned	
		c) Describe the MR estimator (e.g. two-stage least squares, Wald ratio) and related statistics. Detail the included covariates and, in case of two-sample MR, whether the same covariate set was used for adjustment in the two samples	Page 6-7	The inverse variance weighted (IVW) was performed as the main approach in our MR analysis, We also performed sensitivity analyses using MR-PRESSO and MR-Egger methods.
		d) Explain how missing data were addressed	Not mentioned	
		e) If applicable, indicate how multiple testing was addressed	Not mentioned	
7	**Assessment of assumptions**	Describe any methods or prior knowledge used to assess the assumptions or justify their validity	Page 6	In addition, we evaluated instrument strength using the F statistic, where $F=(R^2×(N-k-1))/k(1-R^2)$, to test the significant association of the genetic instruments with the exposure, and SNPs with F <10 were excluded, as an $F \geq 10$ indicates a relatively low risk of weak instrument bias in MR analysis. To avoid potential confounding, we investigated each instrument SNP in the PhenoScanner GWAS database to assess any previous associations ($P<5×10^{-8}$) with plausible confounders.
8	**Sensitivity analyses and additional analyses**	Describe any sensitivity analyses or additional analyses performed (e.g. comparison of effect estimates from different approaches, independent replication, bias analytic techniques, validation of instruments, simulations)	Page 6-7	the MR Egger method was performed to estimate the causal effect, with the capability to identify and account for any directional pleiotropy. The MR Pleiotropy RESidual Sum and Outlier (MR-PRESSO) method was used to evaluate horizontal pleiotropy. the MR-Egger regression intercept term was used to assess the possible presence of horizontal pleiotropy, where deviation from zero (*P*<0.05) indicates directional pleiotropy.
9	**Software and pre-registration**			
		a) Name statistical software and package(s), including version and settings used	Page 6	Outlier pleiotropic SNPs via the heterogeneity test (modified Q statistics) using RadialMR (Version 1.0) with the P value threshold of 0.05. All statistical analyses were performed in R 4.3.1 with the package TwoSampleMR (version 0.5.7) and MRPRESSO (Version 1.0).
		b) State whether the study protocol and details were pre-registered (as well as when and where)	Not mentioned	

RESULTS

10	**Descriptive data**			
	a)	Report the numbers of individuals at each stage of included studies and reasons for exclusion. Consider use of a flow diagram	supplematary data:supplematary table 1 to table 21. as well as figure-4.	The numbers of individuals included study is already provided in the supplematary data:supplematary tables 1 to 21. as well as figure-4.
	b)	Report summary statistics for phenotypic exposure(s), outcome(s), and other relevant variables (e.g. means, SDs, proportions)	Not mentioned	
	c)	If the data sources include meta-analyses of previous studies, provide the assessments of heterogeneity across these studies	Not mentioned	
	d)	For two-sample MR:		
		i. Provide justification of the similarity of the genetic variant-exposure associations between the exposure and outcome samples	Page 5	Genome-wide significant single-nucleotide polymorphisms (SNPs) (P < 5e-08) were extracted from the GWAS summary data, and those with a longer physical distance (> 10,000 kb) and less possibility of linkage disequilibrium (R^2 < 0.001) were retained.
		ii. Provide information on the number of individuals who overlap between the exposure and outcome studies	Page 5	All summary statistics used were GWAS analyses and no sample overlap was observed
11	**Main results**			
	a)	Report the associations between genetic variant and exposure, and between genetic variant and outcome, preferably on an interpretable scale	supplematary data:supplematary table 2, 7, and 14	supplematary data:supplematary table 2, 7, and 14
	b)	Report MR estimates of the relationship between exposure and outcome, and the measures of uncertainty from the MR analysis, on an interpretable scale, such as odds ratio or relative risk per SD difference	supplematary data: supplematary table 17 to table 21. table 1-3. Figure 1-3 and supplematary Figure 4	supplematary data: supplematary table 17 to table 21. table 1-3. Figure 1-3 and supplematary Figure 4
	c)	If relevant, consider translating estimates of relative risk into absolute risk for a meaningful time period	Not mentioned	
	d)	Consider plots to visualize results (e.g. forest plot, scatterplot of associations between	Figure 1-3 and supplematary Figure 1-3 and	Figure 1-3 and supplematary Figure 1-3 and supplematary Figure 5-11

			genetic variants and outcome versus between genetic variants and exposure)	supplematary Figure 5-11	
12	**Assessment of assumptions**				
		a)	Report the assessment of the validity of the assumptions	supplematary data:supplematary table 2, 7, 14, and 20. As well as table 1, 2, and 3.	supplematary data: supplematary table 2, 7, 14 reported the F-statistics for SNP, supplematary data: supplematary table 20 as well as table1, 2, and 3 reported Q-statistics for assessment of heterogeneity.
		b)	Report any additional statistics (e.g., assessments of heterogeneity across genetic variants, such as I^2, Q statistic or E-value)	supplematary data:supplematary table 2, 7, 14, and 20. As well as table 1, 2, and 3.	supplematary data: supplematary table 2, 7, 14 reported the F-statistics for SNP, supplematary data: supplematary table 20 as well as table1, 2, and 3 reported Q-statistics for assessment of heterogeneity.
13	**Sensitivity analyses and additional analyses**				
		a)	Report any sensitivity analyses to assess the robustness of the main results to violations of the assumptions	supplematary data: supplematary table 17 to table 21. As well as table 1-3	MR-Egger, WM and IVW-random-effects model methods were provided as sensitivity analyses. When significant pleiotropy was present, we used the MR-PRESSO method to remove outlier SNPs and calculate corrected ORs and CIs.
		b)	Report results from other sensitivity analyses or additional analyses	supplematary data: supplematary table 17 to table 21. As well as table 1-3	MR-Egger, WM and IVW-random-effects model methods were provided as sensitivity analyses. When significant pleiotropy was present, we used the MR-PRESSO method to remove outlier SNPs and calculate corrected ORs and CIs.
		c)	Report any assessment of direction of causal relationship (e.g., bidirectional MR)	Figure 1, 2, and 3 and supplematary Figure 4	Figure 1, 2, and 3 and supplematary Figure 4 show the results of bidirectional MR
		d)	When relevant, report and compare with estimates from non-MR analyses	Not mentioned	
		e)	Consider additional plots to visualize results (e.g., leave-one-out analyses)	Not mentioned	
	**DISCUSSION**				

14	**Key results**	Summarize key results with reference to study objectives	Page 10	In this MR analysis, we found a causal relationship between IBD and its two subtypes with sarcoidosis. Furthermore, we conducted a mediation analysis and demonstrated for the first time that the effect of IBD on sarcoidosis risk was partially mediated by PBC.
15	**Limitations**	Discuss limitations of the study, taking into account the validity of the IV assumptions, other sources of potential bias, and imprecision. Discuss both direction and magnitude of any potential bias and any efforts to address them	Page 12	Admittedly, there are limitations and restrictions in the current investigation. One of the constraints in this study is that we were compelled to rely solely on GWASs conducted in persons of European ancestry to estimate the causal effects due to the absence of extensive GWASs studies conducted in non-European ancestries. Thus, caution should be exercised when generalizing our findings to other ethnic groups. However, population stratification was not a potential bias in our study since European ancestry was predominant in all datasets. Then, regrettably, we must acknowledge the limitation linked to the use of the IEU GWAS database, which does not allow for subgroup analysis based on gender as conditions of IMIDs that are more commonly found in females than in males. Therefore, we were unable to provide gender-adjusted ORs. Finally, the underlying mechanisms to determine the association of the causal pathway are needed.
16	**Interpretation**			
	a)	Meaning: Give a cautious overall interpretation of results in the context of their limitations and in comparison with other studies	Page 10-12	In this manuscript, the content of this item is discussed a lot, and the MR results are reasonably interpreted by comparing them with several published studies.
	b)	Mechanism: Discuss underlying biological mechanisms that could drive a potential causal relationship between the investigated exposure and the outcome, and whether the gene-environment equivalence assumption is reasonable. Use causal language carefully, clarifying that IV estimates may provide causal effects only under certain assumptions	Page 10-12	This study demonstrated that IBD and its subtypes had an impact on sarcoidosis, which was partially mediated by PBC.
	c)	Clinical relevance: Discuss whether the results have clinical or public policy relevance, and to what extent they inform effect sizes of possible interventions	Page 15	This discovery offered techniques for more effective prevention and intervention of sarcoidosis.
17	**Generalizability**	Discuss the generalizability of the study results (a) to other populations, (b) across other exposure periods/timings, and (c) across other levels of exposure	Page 15	A constraint of the current investigation is that due to the absence of extensive GWA studies conducted in non-European ancestries, we were compelled to rely solely on GWAS conducted in persons of European ancestry to estimate the causal effects.
	**OTHER INFORMATION**			

18	**Funding**	Describe sources of funding and the role of funders in the present study and, if applicable, sources of funding for the databases and original study or studies on which the present study is based	Not mentioned	
19	**Data and data sharing**	Provide the data used to perform all analyses or report where and how the data can be accessed, and reference these sources in the article. Provide the statistical code needed to reproduce the results in the article, or report whether the code is publicly accessible and if so, where	supplematary data: supplematary table 1, as well as Page 12	supplematary table 1 provide the source data URL for download. And page 16 indicated that the further inquiries can be directed to the corresponding author.
20	**Conflicts of Interest**	All authors should declare all potential conflicts of interest	Page 13	All authors declare that they have no competing interests.

This checklist is copyrighted by the Equator Network under the Creative Commons Attribution 3.0 Unported (CC BY 3.0) license.

1. Skrivankova VW, Richmond RC, Woolf BAR, Yarmolinsky J, Davies NM, Swanson SA, et al. Strengthening the Reporting of Observational Studies in Epidemiology using Mendelian Randomization (STROBE-MR) Statement. JAMA. 2021;under review.

2. Skrivankova VW, Richmond RC, Woolf BAR, Davies NM, Swanson SA, VanderWeele TJ, et al. Strengthening the Reporting of Observational Studies in Epidemiology using Mendelian Randomisation (STROBE-MR): Explanation and Elaboration. BMJ. 2021;375:n2233.
